# Phosphorylation of UHRF2 affects malignant phenotypes of HCC and HBV replication by blocking DHX9 ubiquitylation

**DOI:** 10.1038/s41420-023-01323-2

**Published:** 2023-01-24

**Authors:** Kejia Wu, Yiqi Zhang, Yuxin Liu, Qingxiu Li, Yong Chen, Juan Chen, Changzhu Duan

**Affiliations:** 1 Department of Cell Biology and Medical Genetics, Molecular Medicine and Cancer Research Center, Chongqing Medical University, Chongqing, 400016 China; 2 Department of Hepatobillary Surgery, The First Affiliated Hospital, Chongqing Medical University, Chongqing, 400016 China; 3 Key Laboratory of Molecular Biology for Infectious Diseases (Ministry of Education), Institute for Viral Hepatitis, Department of Infectious Diseases, The Second Affiliated Hospital, Chongqing Medical University, Chongqing, 400016 China

**Keywords:** Oncogenesis, Tumour virus infections, Hepatocellular carcinoma

## Abstract

Hepatitis B virus (HBV) infection is one of main contributors to poor prognosis and rapid progression of hepatocellular cancer (HCC). We previously identified the important role of the phosphorylation of ubiquitin-like with PHD and ring finger domains (UHRF2) in HBV-associated HCC. In this study we identify upregulated UHRF2 protein levels in HBV-associated HCC cells and tissues. UHRF2 overexpression promotes the viability, proliferation, migration and invasiveness of HBV-positive HCC cell lines, and enhances HBV DNA replication. To obtain a comprehensive understanding of the interaction networks of UHRF2 and their underlying mechanism, this study suggests that UHRF2 facilitates the ubiquitin-proteasome-mediated proteolysis of DExD/H (Asp-Glu-Ala-His) -box helicase enzyme 9 (DHX9). However, phosphorylation of UHRF2 by HBx at S643 inhibits E3 ubiquitin ligase activity of UHRF2 and improves DHX9 protein stability. Furthermore, results suggest that HBx promotes phosphorylation of UHRF2 by the ETS1-CDK2 axis through the downregulation of miR-222-3p in HBV-associated HCC specimens and cells. Our findings suggest that HBx-induced phosphorylation of UHRF2 S643 acts as a “switch” in HBV-associated HCC oncogenesis, activating the positive feedback between phosphorylated UHRF2 and HBV, provide evidence that UHRF2 is a new regulator and a potential prognostic indicator of poor prognosis for HBV-associated HCC.

## Introduction

HBV virus is a semi-closed circular DNA envelope that causes both acute and chronic hepatitis. The Hepatitis B virus DNA consists of four regions, including C, P, S, and X, which encode HBcAg (HBc), Hepatitis B virus DNA polymerase (HBp), HBsAg (HBs), and Hepatitis B virus X protein (HBx) [1]. HBx came into the attention of researchers as an important small regulatory protein. It promotes HBV replication by regulating the transcriptional process mainly through binding HBV covalently closed circular DNA (cccDNA) [2]. Moreover, HBx contributes to the development of HCC by regulating the aberrant expression of host proto-oncogenes or oncogenes, influencing intracellular signaling, accelerating cell proliferation and driving the cell migration and invasion pathways of HCC [3–6]. One of the most common post-intervention outcomes of chronic HBV infection is hepatocellular carcinoma [7, 8]. Hepatocellular carcinoma is one of the top two causes of cancer-related tumor death [9]. In East Asia, chronic HBV infection is one of the major causes of HCC, and the mechanism of HBV-positive HCC development is not completely understood and deserves further investigation [10].

Ubiquitin-like with PHD and ring finger domains (UHRF2) is a ubiquitin-protein E3 ligase that plays multiple roles in cancer development [11–16]. A high level of UHRF2 is considered to be associated with a poor prognosis in many cancers, and its auto-ubiquitylation properties result in generally low and unstable protein levels in cells and tissues [17]. After identifying the phosphorylation site of UHRF2 on S643, a significant enhancement in UHRF2 protein stability was observed in our last study. Previous studies suggest that HBx promotes UHRF2 phosphorylation and inhibits its auto-ubiquitylation in HBV-positive HCC. Therefore, this study focuses on the regulation of UHRF2 phosphorylation levels in specific cancer processes. However, the specific process by which HBx regulates UHRF2 phosphorylation and the mechanism of phosphorylated UHRF2 promotes HBV-associated HCC is not clear.

In this study, we explore the positive feedback between phosphorylated UHRF2 and HBV triggered by UHRF2. Furthermore, the results demonstrate that UHRF2 phosphorylation inhibits DHX9 ubiquitylation by upregulating DHX9 protein levels on HBV replication with a facilitation effect on HBV-associated HCC. HBx regulates UHRF2 phosphorylation by affecting miR-222-3p levels. The results shed light on the role of UHRF2 in related disease processes and identify the phosphorylation-to-ubiquitylation “switch” UHRF2 as an important link in the disease process of HBV and HBV-associated HCC that drives disease procession.

## Results

### High expression of UHRF2 in HBV-positive HCC is associated with a poor prognosis

UHRF2 is a ubiquitin-protein ligase E3, which plays a critical role in the development of HBV-associated HCC [16–18]. The expression of UHRF2 in HBV-positive HCC tumors and non-tumor tissues was investigated to determine its biological role.

**Fig. 1:**
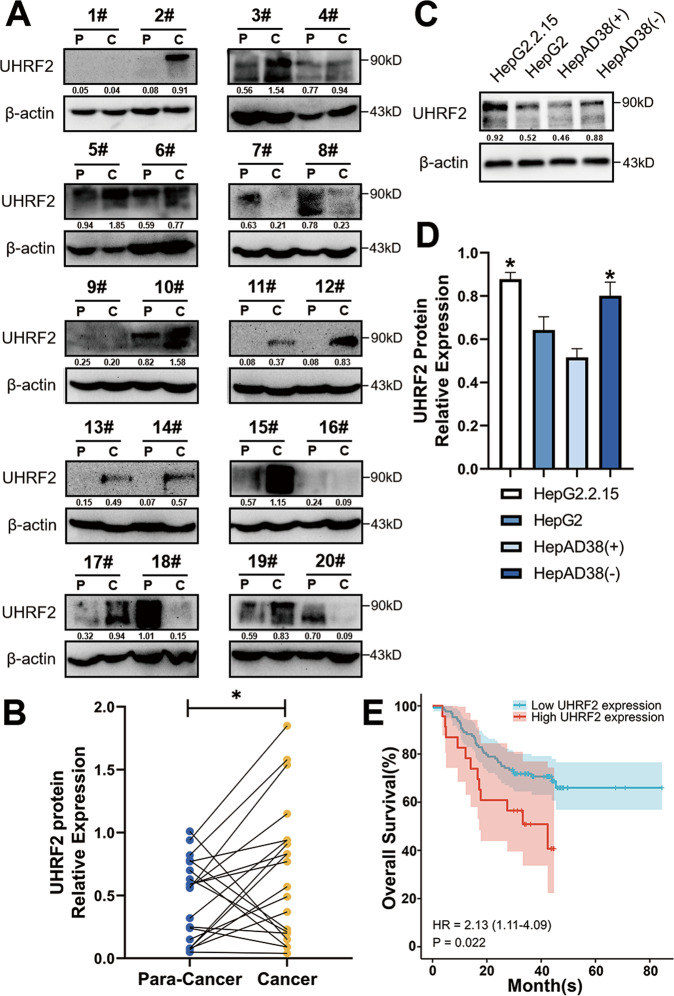
High expression of UHRF2 in HBV-positive HCC associated to poor prognosis of HCC. **A** UHRF2 expression was amplified in HBV-positive HCC tissues. Expression of UHRF2 protein in HBV-positive HCC tumor specimens (C) and matched cancer adjacent tissues (P). Protein levels was detected by western blot. **B** UHRF2 protein level was upregulated in HBV-associated HCC. UHRF2 protein levels were calculated by the gray values of indicated bands and values normalized to β-actin. statistical comparisons were using paired *t*-test. *, *p* < 0.05. **C** UHRF2 expression was increased in HBV-positive hepatoma cell lines. Expression of UHRF2 protein in Hepatoma cell lines. Protein levels was detected by western blot, Image J gray-scale scanning was used to obtain the gray values of UHRF2 bands and values normalized to β-actin. **D** Statistical comparisons were using Student’s *t* test between HBV-positive HCC cell lines and HBV-negative HCC cell lines. statistical comparisons were using Student’s *t* test. *, *p* < 0.05. **E** High UHRF2 expression in HBV-positive HCC associated to poor prognosis of HCC. A Kaplan–Meier overall survival curve (top panel) and recurrence-free survival curve (bottom panel) according to UHRF2 expression in CPTAC database.

TCGA database analysis showed that UHRF2 mRNA levels were significantly upregulated in HCC, especially in HBV-associated HCC (Supplementary Fig. 1A, B). We collected paired hepatocellular carcinoma tissues with corresponding paracancerous tissues from 20 HBV-positive HCC patients during 2020–2021 and analyzed the levels of UHRF2 in these tissues by Western blot assay. Of the 20 HBV-positive HCC patients examined, UHRF2 expression appears to be higher at the HCC site than at the periphery in about 10 patients. However, UHRF2 expression in the other patients was higher at the periphery or similar in both tissues (Fig. 1A, B). Supplementary Table 1 displays the baseline parameters. UHRF2 levels were noticeably higher in HCC tissues. Furthermore, the UHRF2 protein levels were higher in HBV-positive HCC cell lines than in HBV-negative ones (Fig. 1C, D). The CPTAC (Clinical Proteomic Tumor Analysis Consortium) database was used to analyze the prognosis of HBV-positive patients with different UHRF2 protein levels. Kaplan–Meier survival analysis demonstrated higher overall survival rate in the UHRF2 high expression than in the UHRF2 low expression group (Fig. 1E), which indicate that high expression of UHRF2 is associated with poor prognosis in patients with HBV-associated HCC.

### UHRF2 promotes HBV virus replication in hepatoma cell lines

**Fig. 2:**
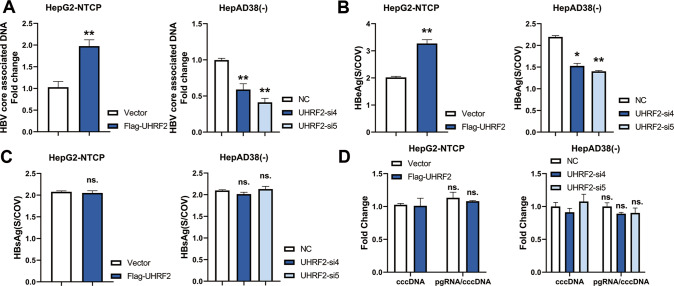
UHRF2 initiates HBV virus replication. HepG2-NTCP cells were transfected with Flag-UHRF2 plasmid or its vector control 48 h post-infection of 10^3^ HBV genome equivalent/cell of HBV. HepAD38(-) cells were transfected with UHRF2 siRNAs and their vector control. Both UHRF2 overexpression plasmid and siRNAs efficiencies are validated. HepG2-NTCP cells and HepAD38(-) cells were harvested 72 h post-transfected. HepG2-NTCP, HepG2 cells with sodium taurocholate cotransporting polypeptide (NTCP). HepAD38(-), HepAD38 cells cultured for more than 7 days in the absence of tetracycline. **A** UHRF2 upregulated cellular HBV DNA level of HBV-positive cell lines. HBV core associated DNA in HepG2-NTCP (left panel) and HepAD38(-) (right panel) cytoplasmic fractions were measured by quantitive PCR. Data are shown as the fold induction compared to vector controls. (*n* = 3). **B** UHRF2 upregulated cell supernatant HBeAg level. HBeAg in the HepG2-NTCP (left panel) and HepAD38(-) (right panel) cell supernatant was detected by ELISA. S/COV, ratio of sample OD value to cut-off OD value. (*n* = 3). **C** UHRF2 expression had no effect on HBV cccDNA and HBV RNA level. Level of HepG2-NTCP (left panel) and HepAD38(-) (right panel) cellular HBV cccDNA and preC-pgRNA were detected by quantitive PCR. The ratio of preC- pgRNA to cccDNA were analyzed. Data are shown as the fold induction compared to indicated vector controls. (*n* = 3). **D** UHRF2 expression had no effect on HBsAg in cell supernatant. HBsAg in the HepG2-NTCP (left panel) and HepAD38(-) (right panel) cell supernatants were detected by ELISA. S/COV, ratio of sample OD value to cut-off OD value. (*n* = 3). All statistical comparisons were using Student’s *t* test. ns. not significant. *, *p* < 0.05, **, *p* < 0.01.

To study the activity of UHRF2 on Hepatitis B virus replication, UHRF2 was overexpressed in HBV-infected HepG2-NTCP, while UHRF2 was knocked down in HepAD38(-) cells. These two cell lines can produce mature HBV virions. UHRF2 overexpression or siRNA efficiency were validated by qRT-PCR and western blot (Supplementary Fig. 2A). UHRF2 expression in both cell lines was positively correlated with intracellular HBV-associated core DNA levels of hepatoma cells (Fig. 2A) and HBeAg (Fig. 2B) levels in cell supernatant, which indicates the replication ability of HBV. However, UHRF2 had no significant effect on intracellular HBV preC-pgRNA (pre-core RNA or pregenomic RNA) to cccDNA ratio (Fig. 2C) and HBsAg level in cell supernatant (Fig. 2D). This suggested that UHRF2 did not affect expression of cccDNA and HBV transcription. Taken together, the results suggest that UHRF2 has potential to affect the replication of HBV.

### HBx and UHRF2 enhances proliferation, migration, invasion, and tumorigenesis of hepatoma cells

**Fig. 3:**
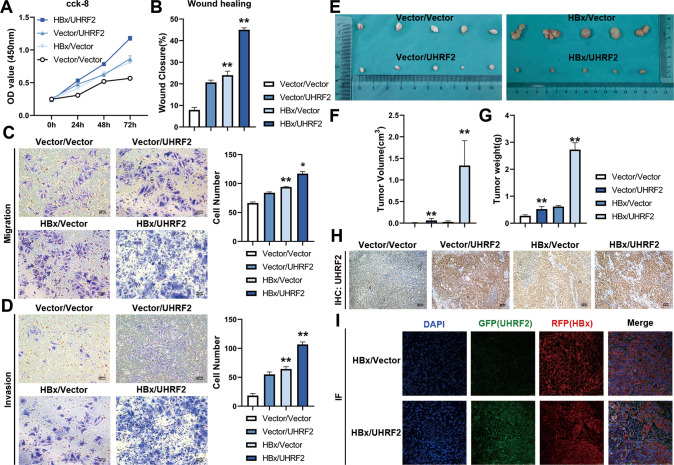
HBx and UHRF2 enhances proliferation, migration, invasion, and tumorigenesis of hepatoma cells. **A–D** Huh7 cells were co-infected with UHRF2 and HBx overexpression lentivirus and stably overexpressed Huh7 cell lines were established. We cultured infected cells in puromycin (2 ng/mL) and blasticidin (1 ng/mL) containing medium for 2 weeks for following assays. Cell Counting Kit-8 assays (**A**), wound healing assays (**B**), transwell migration (**C**) and invasion (**D**) were performed to measure cell viability of HepG2.2.15 (left panel) and HepAD38(-) (right panel). **E**–**G** Subcutaneous xenografts from the indicate cell clones excised from nude mice (*n* = 5) (**E**). Indicated tumor volume (**F**) and wight (**G**) were measured. Tumor volume was calculated as 1/2 × A × B^2^ (A, long diameter; **B,** short diameter). **H**, **I** Protein expression in subcutaneous xenograft tumors were confirmed. The efficiency of UHRF2 overexpressed in tumors was verified by analyzing the level of protein by immunohistochemistry (**H**). Immunofluorescence substantiated the level of protein in HBx/UHRF2 co-overexpressed tumors (**I**). For **A**–**D**, Statistical comparisons were using Welch’s two sample *t*-test. *, *p* < 0.05, **, *p* < 0.01. For **F** and **G**, Statistical comparisons were using Student’s *t* test. *, *p* < 0.05, **, *p* < 0.01.

Previous studies have indicated that HBx promotes HCC progression and is closely related to UHRF2 levels in HBV-positive HCC [17]. As UHRF2 may play a role in HBV-associated HCC by investigating its possible biological functions, Huh7 cells were transduced with lentiviral to overexpress UHRF2 and ectopic HBx. The qRT-PCR results revealed that the UHRF2 overexpressed Huh7 cell lines were successfully established (Supplementary Fig. 2C), and the cck-8 assay revealed that UHRF2 overexpression promoted Huh7 cell proliferation. HBx and UHRF2 co-overexpression greatly enhanced this effect (Fig. 3A). High UHRF2 levels promoted the migration and invasion of HBx positive or negative Huh7 cells in wound healing assays (Fig. 3B) and transwell assays with or without Matrigel to assess cell migration and invasion capacity (Fig. 3C, D).

To assess the in vivo tumorigenic capacity of UHRF2, subcutaneous xenografted nude mice models were established (Fig. 3E). UHRF2-overexpressed cells, UHRF2/HBx co-overexpressed cells and two negative control cell lines were injected into the right armpit of nude mice (5 mice in each group). The volume and weight of the xenograft tumors were significantly improved in Huh7 cells infected with UHRF2 lentivirus compared to the control group (Fig. 3F, G). UHRF2 and ectopic HBx expression were also measured in the xenografted tumor by immunohistochemistry (Fig. 3H) and immunofluorescence (Fig. 3I). UHRF2 overexpression also significantly increased the volume and weight of xenograft tumors in HBx-positive Huh7 cells. The results showed significantly greater tumor growth in UHRF2-overexpressed cells, and the co-overexpression of HBx and UHRF2 further contributed to this effect.

### UHRF2 interacts with DHX9 and downregulates DHX9 protein levels

**Fig. 4:**
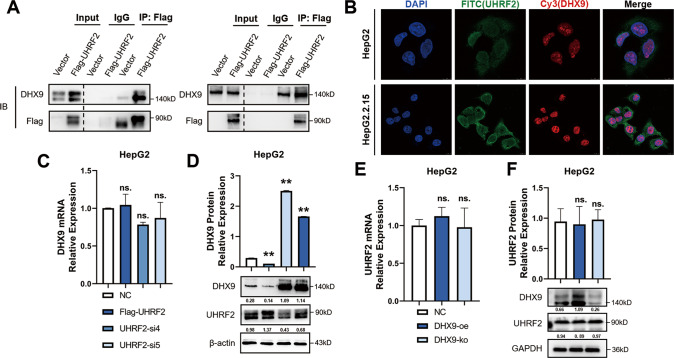
UHRF2 interacts with DHX9 and downregulates DHX9 protein level. **A** UHRF2 interacted with DHX9. Co-IP assay (top panel) and Reverse co-IP (bottom panel) were performed and proteins were detected by western blot. **B** UHRF2 co-localized with DHX9. Immunofluorescence staining indicated the localization of UHRF2 and DHX9 in HepG2 (top panel) and HepG2.2.15 (bottom panel). **C** UHRF2 overexpression and knockdown have no effect on DHX9 mRNA level (*n* = 3). DHX9 mRNA levels were analyzed by qRT-PCR and values normalized to GAPDH (△△CT). **D** UHRF2 overexpression suppressed DHX9 protein expression and knockdown promoted. DHX9 protein levels are detected by western blot (bottom panel), Image J gray-scale scanning was used to obtain the gray values. DHX9 protein relative expressions (top panel, *n* = 3) were calculated by the gray values of indicated bands and values normalized to β-actin. **E** DHX9 overexpression or knockout have no effect on UHRF2 mRNA level (*n* = 3). UHRF2 mRNA levels were analyzed by qRT-PCR and values were normalized to GAPDH (△△CT). oe overexpression, ko knockout. **F** DHX9 overexpression or knockout have no effect on UHRF2 protein level. DHX9 protein levels are detected by western blot (bottom panel), Image J gray-scale scanning was used to obtain the gray values. DHX9 protein relative expressions (top panel, *n* = 3) were calculated by the gray values of indicated bands and values normalized to β-actin. For **C**–**F**, statistical comparisons were using Student’s *t* test between groups. ns. not significant. **, *p* < 0.01.

Since UHRF2 protein levels are upregulated in HBV-associated HCC as well as in HBV-positive hepatoma cells, we identified the UHRF2 substrates which may modulate the process of HBV-associated liver diseases from hepatitis to HBV-associated HCC. DHX9 is generally considered a protein that contributes to HBV DNA replication and regulates the anti-HBV effect of apolipoprotein B mRNA editing enzyme catalytic subunit 3B (APOBEC3B), which is recognized as an important host protein that regulates the disease process of HBV [19, 20]. Our previous mass spectrometry analysis results of co-immunoprecipitating proteins of UHRF2 identified DHX9 for the following study [21]. The interaction between UHRF2 and DHX9 protein was validated by co-immunoprecipitation (co-IP) in HepG2 cells (Fig. 4A) and co-localization by indirect immunofluorescence in both HBV-negative HepG2 cells and HBV-positive HepG2.2.15 (Fig. 4B). Consequently, to determine the effects of UHRF2 on HBV-associated HCC development and HBV replication, DHX9 expression was detected after overexpressing or knocking down UHRF2 in HepG2 cells. The results show that UHRF2 has no effect on the transcriptional regulation of DHX9 (Fig. 4C). However, UHRF2 knockdown increased DHX9 protein levels in HepG2 cells, whereas overexpression of UHRF2 reduced DHX9 protein levels (Fig. 4D). UHRF2 reduces DHX9 expression at the post-transcriptional level.

In addition, we verified that DHX9 expression did not have effect on the mRNA (Fig. 4E) or protein levels (Fig. 4F) of UHRF2. We detected UHRF2 mRNA and protein levels in DHX9 overexpression or knockout HepG2 cells. Results showed that UHRF2 was not regulated by DHX9 expression, further suggests that UHRF2 is the upstream regulator of DHX9.

### UHRF2 promotes DHX9 degradation via the ubiquitin-proteasome pathway in HBV-negative hepatoma cells

**Fig. 5:**
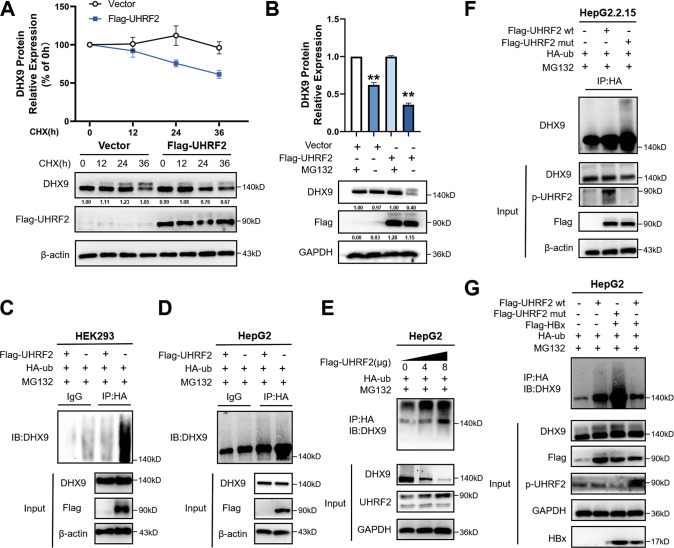
UHRF2 promotes DHX9 ubiquitylation while S643 phosphorylated UHRF2 inhibits DHX9 degradation. **A** UHRF2 suppressed half-life of DHX9 protein. Post-transfected of Flag-vector or Flag-UHRF2 plasmid, cycloheximide (CHX; 100 μg/mL) was added for indicated times. The gray values of DHX9 bands were analyzed by Image J gray-scale scanning and values normalized to β-actin. The protein decay rate of DHX9 is calculated as (gray value at indicated time/gray value at 0 h) * 100% (*n* = 3). **B** UHRF2 overexpression promoted DHX9 proteasome degradation. Cells were pre-incubated with MG-132 (20 μmol/L) for 12 h and whole cell lysates were extracted for western blot. The gray values of DHX9 bands were analyzed by Image J gray-scale scanning. The relative expression of DHX9 protein is calculated as gray value of group vector with MG132/gray value of indicated group (*n* = 3). **C–D** UHRF2 promotes DHX9 protein ubiquitylation as E3 ubiquitin ligase. HEK293 (**C**) and HepG2 (**D**) cells were co-transfected with indicated plasmid and pre-incubated with MG-132 (20 μmol/L) for 12 h. 72 h post-transfection, Cell lysates were immunoprecipitated by anti-HA and immunoblotted by anti-DHX9. Both whole cell lysates and immunoprecipitated proteins were analyzed by western blot. β-actin was used to indicate the amount of loading proteins for input. **E** UHRF2 promotes ubiquitylation of DHX9 in a dosage-dependent manner. HepG2 cells were co-transfected with 0, 4, or 8 μg of Flag-UHRF2 and HA-ub. 60 h post-transfection, cells were treated with MG-132 (20 μmol/L) for 12 h. 72 h post-transfection, Cell lysates were immunoprecipitated by anti-HA and immunoblotted by anti-DHX9. Both whole cell lysates and immunoprecipitated proteins were analyzed by western blot. β-actin was used to indicate the amount of loading proteins for input. Flag-UHRF2 wt, Flag tagged UHRF2 wild type plasmid; Flag-UHRF2 mut, Flag tagged UHRF2 plasmid with serine 643 mutant to alanine. **F** HBV inhibited E3 ubiquitin ligase activity of UHRF2. UHRF2 S643 mutant increased DHX9 protein ubiquitylation while UHRF2 wild type inhibited in HBV-positive hepatoma cells. HepG2.2.15 cells were co-transfected with indicated plasmid and pre-incubated with MG-132 (20 μmol/L) for 12 h. DHX9 ubiquitylation were detected as described by **C** and **D**. p-UHRF2, antibody of phosphorylated UHRF2 at serine 643. **G** HBx inhibited E3 ubiquitin ligase activity of UHRF2 in HBV-negative cell lines. HepG2 cell were co-transfected with indicated plasmids and treated and detected as described by **C** and **D**. For **A** and **B**, Statistical comparisons were using Student’s *t* test. **, *p* < 0.01.

The underlying mechanism via which UHRF2 regulates DHX9 protein levels was next investigated. Cycloheximide (CHX, a translation inhibitor) was employed to block protein synthesis and detect whether UHRF2 could shorten DHX9 protein half-time. The results revealed that ectopic over-expression of UHRF2 attenuated the degradation of DHX9 in HepG2 cells (Fig. 5A), suggesting that UHRF2 downregulates DHX9 protein by promoting protein degradation.

Therefore, we explored whether E3 ubiquitin-protein ligase UHRF2 regulates DHX9 degradation through the ubiquitin-proteasome pathway. MG132 was used to detect whether UHRF2 is degraded via the proteasome pathway. The proteasome inhibitor MG132 effectively abolished UHRF2 overexpression-induced DHX9 degradation (Fig. 5B). Subsequently, immunoprecipitation and western blot analysis were performed in HEK293 cells and HepG2 cells, which co-transfected with UHRF2 (Flag-UHRF2) and ubiquitin (HA-ub) plasmid. In these two HBV-negative cell lines, UHRF2 was observed to promote DHX9 ubiquitylation (Fig. 5C, D), suggesting that DHX9 can be ubiquitylated by UHRF2 in vivo. In addition, UHRF2 was found to promote ubiquitylation of DHX9 in a dosage-dependent manner (Fig. 5E). Overall, we conclude that UHRF2 modulates the DHX9 protein levels via the ubiquitin-proteasome pathway in HBV-negative hepatoma cells.

### HBx induced phosphorylation of UHRF2 S643 inhibits degradation of DHX9 by blocking DHX9 ubiquitylation in HBV-Positive hepatoma cells

However, DHX9 has been reported as a facilitator of HCC with HBV in previous studies. This is not consistent with our previous findings. We then shifted our focus to the effect of HBV infection on the E3 ubiquitin ligase UHRF2. Evidence shows that HBx protein interferes with the ubiquitin-proteasome system (UPS) mediates host protein degradation, and promotes viral replication or proliferation in hepatocellular carcinoma cells [4–6]. In a previous study, HBx was found to inhibit ubiquitin-protein ligase E3 activity of the RING domain by phosphorylating UHRF2 at serine 643. Thus, we speculated HBV may inhibit the ubiquitin-proteasome degradation of DHX9 by phosphorylating UHRF2 at serine 643, and upregulating DHX9 protein levels to promotes HBV replication and HCC development. To elucidate that HBx-induced phosphorylation of UHRF2 can inhibit the degradation of DHX9 protein, we designed Flag-tagged UHRF2 plasmids with serine-alanine mutations at position 643 (S643A). Immunoprecipitation and western blot analysis were performed to observe the ubiquitylation levels of DHX9 in HBV-positive cell line HepG2.2.15 treated by Flag-UHRF2 wild type (Flag-UHRF2 wt), and Flag-UHRF2 S643A mutant (Flag-UHRF2 mut) (Fig. 5F). Interestingly, in transfected HepG2.2.15 cells, only Flag-UHRF2 S643A mutant plasmid influenced the ubiquitylation of DHX9. Indicating that the ubiquitylation of DHX9 by UHRF2 is mainly dependent on UHRF2 S643. The same conclusion was obtained by western blot analysis.

We next hypothesized that phosphorylation of UHRF2 S643 induced by HBx made UHRF2 to lose most of its E3 ubiquitin ligase activity. The regulatory role of HBx-induced phosphorylation of UHRF2 S643 on DHX9 ubiquitylation was confirmed (Fig. 5G). Immunoprecipitation analysis showed that transfection with Flag-UHRF2 S643A effectively reversed DHX9 ubiquitylation in HBV-negative HepG2 cells after ectopic HBx overexpression. The same conclusion was obtained by western blot. The upregulation of serine phosphorylation levels at UHRF2 position 643 caused by HBV inhibits the degradation of DHX9 by suppressing ubiquitin-protein ligase E3 activity of UHRF2. In summary, expression of ectopic HBx in HBV-negative HepG2 cells reversed the ubiquitination of DHX9 by UHRF2, confirming our conjecture about HBx inhibits degradation of DHX9 by phosphorylating UHRF2 S643.

### UHRF2 affects HBV replication and HCC proliferation, migration and invasion mainly through regulating of DHX9 protein level

**Fig. 6:**
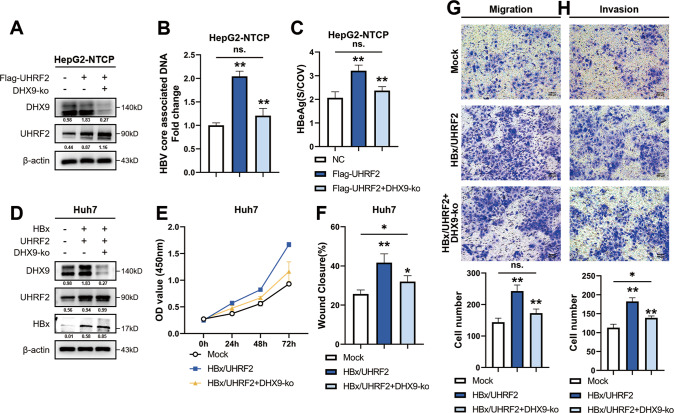
DHX9 knockout partially rescued promotion of HBV replication and HCC proliferation, migration, invasion caused by UHRF2 overexpression. HepG2-NTCP cells were transfected with Flag-UHRF2 plasmids or Flag-UHRF2 and DHX9 Crisper-Cas9 knockout plasmid 48 h post-infection of 10^3^ HBV genome equivalent/cell of HBV. Both UHRF2 overexpression plasmid and siRNAs efficiencies are validated. Both cells were harvested 72 h post-transfected. **A** DHX9 rescues the changes in HBV DNA levels in HBV-positive cells caused by UHRF2. HBV core associated DNA in HepG2-NTCP cytoplasmic fractions were measured by quantitive PCR. Data are shown as the fold induction compared to nonsense control group (NC). (*n* = 3). **B** Expression of DHX9 rescues the changes in HBeAg levels of HBV-positive cells supernatant caused by UHRF2. HepG2-NTCP supernatant was detected by ELISA. S/COV, ratio of sample OD value to cut-off OD value. (*n* = 3). **C** Protein expression levels of HepG2-NTCP cells after transfection were determined by Western blot. Image J gray-scale scanning was used to obtain the gray values, and the relative expression of each protein were listed below the bands which normalized to β-actin. Huh7 cells were co-infected by HBx and UHRF2 overexpression lentivirus and stably overexpression UHRF2 and ectopic HBx. DHX9 Crisper-Cas9 knockout plasmid was transfected to these Huh7 cells to block DHX9 protein expression. Cells were harvested 48 h post-transfected. **D** Protein levels of indicated protein in treated Huh7 cells were determined by Western Blot. **E–G** Image J gray-scale scanning was used to obtain the gray values, and the relative expression of each protein were listed below the bands which normalized to β-actin. Cell Counting Kit-8 assays (**E**), wound healing assays (**F**), Transwell migration (**G**) and invasion (**H**) were performed to measure cell viability of Huh7. All statistical comparisons were using Welch’s two sample *t*-test. ns. not significant; *, *p* < 0.05; **, *p* < 0.01.

We next investigated whether UHRF2 affects HBV replication and the malignant phenotype of HCC by regulating DHX9. First, we verified in HBV-infected HepG2-NTCP cell lines whether downregulation of DHX9 could revert the promotion of HBV replication capacity caused by UHRF2 upregulation. We knocked out DHX9 by Crisper-Cas9 in UHRF2 overexpressed HepG2-NTCP cells (Fig. 6A). Cellular HBV DNA levels (Fig. 6B) were measured after 48 h of treatment together with HBeAg levels in the supernatant (Fig. 6C). Both results showed that most of the HBV replication promotions caused from UHRF2 However, based on the findings in Fig. 5, we found that the UHRF2-DHX9 regulation is an HBV-manipulated mechanism which results in relative upregulation of DHX9 levels through inhibition of degradation. We suggest that the reversal of HBV replication facilitation via DHX9 may be partially independent of the upregulation of UHRF2. We secondly knocked out the DHX9 gene in Huh7 cells that stably overexpress UHRF2 and ectopic HBx and observed changes in cell functions (Fig. 6D). cck-8 assay (Fig. 6E) showed that DHX9 knockout partially rescued the cell proliferation promotion caused by UHRF2 upregulation. Wound healing assay (Fig. 6F), Transwell assay with or without Matrigel (Fig. 6G, H) also showed that the cell migration and invasion promotion caused by HBx-UHRF2 upregulation was also partially reversed by DHX9 knockout. These results preliminarily indicate that UHRF2 improved the malignant phenotype of HBV-positive hepatocellular carcinoma cells in part through DHX9. We propose that it is potentially because DHX9 affects HCC development mostly by promoting HBV replication, while UHRF2 also influences HCC development through other classical oncogenic signaling pathways [15, 21]. Therefore, it is excluded that upregulation of UHRF2 simultaneously promotes the development of hepatocellular carcinoma independently of DHX9.

### HBx phosphorylates UHRF2 via the miR-222-3p-ETS1-CDK2 axis

**Fig. 7:**
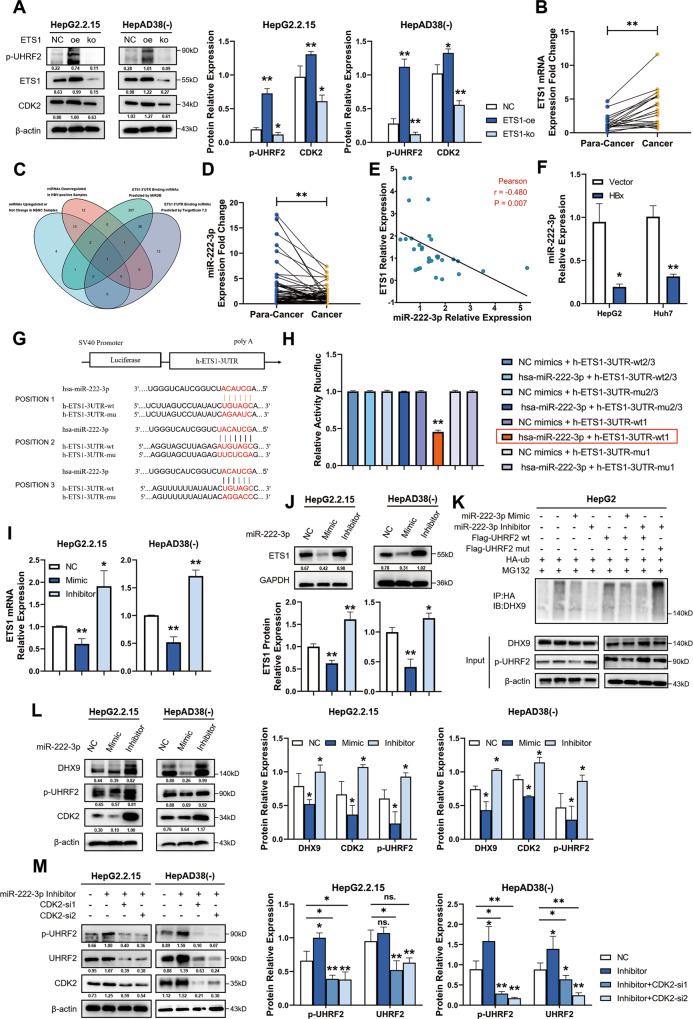
HBx phosphorylates UHRF2 via miR-222-3p-ETS1-CDK2 axis. **A** ETS1 promotes phosphorylation of UHRF2 S643 by upregulating CDK2 protein level. ETS1 overexpression or knockout modulated phosphorylation level of UHRF2 S643 and CDK2 protein level. Protein of HepG2.2.15 (left panel) and HepAD38(-) (right panel) were detected by western blot, Image J gray-scale scanning was used to obtain the gray values of target protein bands and values normalized to β-actin (*n* = 3). p-UHRF2, antibody of phosphorylated UHRF2 at serine 643. **B** ETS1 mRNA level was suppressed in HBV-associated HCC. The mRNA expression of ETS1 in 20 pairs of HBV-positive HCC and corresponding adjacent liver tissues was determined by qRT-PCR and values were normalized to GAPDH. **C** Venn diagram depicts the overlap of different groups of miRNAs. The intersection of miRNAs whose expression downregulated in HBV-positive HCC tissues to NBNC-HCC tissues from GSE147892 with the genes identified as ETS1 3’UTR binding miRNAs predicted by TargetScan (ver7.2) and miRDB. **D** MiR-222-3p level was amplified in HBV-associated HCC. The expression of miR-222-3p in 60 pairs of HBV-positive HCC and corresponding adjacent liver tissues was determined by qRT-PCR and values were normalized to U6. **E** ETS1 mRNA was negatively correlated with miR-222-3p. The RNA expression of ETS1 and miR-222-3p in 20 HBV-positive cancer tissues were analyzed by qRT-PCR and values were normalized. Then, correlation between miR-222-3p and ETS1 expression was analyzed by Spearman’ s correlation analysis. **F** Ectopic HBx overexpression downregulated miR-222-3p level. 48 h post-transfected, miR-222-3p level was detected by qRT-PCR and values were normalized to U6 (*n* = 3). **G**, **H** miR-222-3p directly bind to ETS1 promotor and suppressed ETS1 mRNA level. A duel luciferase activity of the reporters containing the ETS1 3’UTR wild-type 1 but not ETS1 mutant 1. A schematic diagram of miR-222-3p binds to ETS1 3’UTR and corresponding mutations sites are presented in **G**. Activity of Rluc/fluc were detected and normalized (**H**) (*n* = 3). wt wild-type position, mu mutation position. **I**, **J** Ectopic expression of miR-222-3p or antagonism (inhibitor) of cellular miR-222-3p affected ETS1 expression. 48 h post-transfected, HepG2.2.15 (left panel) and HepAD38(-) (right panel) cells was analyzed by qRT-PCR (**I**) and western blot (**J**). mRNA levels were normalized to GAPDH (*n* = 3), Image J gray-scale scanning was used to obtain the gray values of UHRF2 bands and values normalized to β-actin (*n* = 3). **K** MiR-222-3p regulated DHX9 protein ubiquitylation with HBx. HepG2 cells were co-transfected with ectopic miR-222-3p mimic or antagonism of miR-222-3p and pre-incubated with MG-132 (20 μmol/L) for 12 h. 72 h post-transfectection, Cell lysates were immunoprecipitated by anti-HA and immunoblotted by anti-DHX9. Both whole cell lysates and immunoprecipitation proteins were analyzed by western blot. β-actin was used to indicate the amount of input proteins. **L** Ectopic expression of miR-222-3p or antagonism of cellular miR-222-3p affected UHRF2 S643 phosphorylation, regulated DHX9 and CDK2 protein level. Protein of HepG2.2.15 (left panel) and HepAD38(-) (right panel) were detected by western blot. Image J gray-scale scanning was used to obtain the gray values of UHRF2 bands and values normalized to β-actin (*n* = 3). **M** Rescue of upregulation of CDK2 protein expression caused by miR-222-3p inhibitor partially reversed UHRF2 S643 phosphorylation and UHRF2 degradation. Indicated protein levels of HepG2.2.15 (left panel) and HepAD38(-) (right panel) were detected by western blot. Image J gray-scale scanning was used to obtain the gray values of UHRF2 bands and values normalized to β-actin (*n* = 3). All the statistical comparisons were using Student’s *t* test. *, *p* < 0.05, **, *p* < 0.01.

After clarified that HBx-induced phosphorylation of UHRF2 at serine 643 has an important impact on both HBV and HCC disease processes, we wondered how HBx regulates phosphorylation of UHRF2. Recent studies have revealed that HBV promotes aberrant expression of endogenous host genes through HBx-induced downregulation of multiple miRNAs [22]. A previous study shows that HBx can promote ETS proto-oncogene 1 (ETS1) expression upregulation [23], and our previous research confirmed that ETS1 promotes phosphorylation of UHRF2 S643 by upregulating the CDK2 protein levels [16]. We analyzed the level of CDK2 protein and phosphorylation of UHRF2 at S643 position (p-UHRF2) after manipulating ETS1 protein level by overexpression plasmid and CRISPER-Cas9 knockdown plasmid, and obtained results consistent with those in previous studies (Fig. 7A). Data from RNA-Seq datasets from the Cancer Genome Atlas (TCGA) project indicated that ETS1 expression was upregulated in HBV-associated HCC compared to non-HBV-infected HCC (Supplementary Fig. 3C). Subsequently, the ETS1 expression in the experiment specimens was analyzed. ETS1 mRNA levels were significantly upregulated in HBV-associated HCC cancer tissues relative to the pan-cancers (Fig. 7B). Also, ETS1 mRNA levels in cells were consistent with results in tissues (Supplementary Fig. 3D).

However, HBx does not directly bind to ETS1 but upregulates ETS1 expression through a trans-activation manner [24, 25]. Therefore, we hypothesized that HBx upregulates ETS1 by regulating miRNA expression. First, miRNA expression was analyzed by a microarray gene expression dataset from the NCBI Gene Expression Omnibus (GEO) database (GSE147829). miRNA levels in cancer and pan-cancer samples with different HBV infection profiles were analyzed to screen miRNAs that were simultaneously upregulated in HBV-associated HCC and upregulated or unchanged in non-HBV and non-HCV liver cancer (Supplementary Fig. 3A, B). Next, these miRNAs were intersected with the miRNAs screened from the TargetScan database (ver.7.2) and the miRDB database that bind to the 3’UTR end of ETS1 (Fig. 7C). Finally, the miR-222-3p miRNA was identified and selected for further experiments.

To investigate the expression of miR-222-3p in HBV-associated HCC, the difference in miR-222-3p expression in 60 pairs of HBV-associated HCC cancer and paracancerous tissues was analyzed by qRT-PCR. Results showed that miR-222-3p expression was significantly downregulated in cancer tissues compared to paracancerous tissues (Fig. 7D). Furthermore, the miR-222-3p levels were investigated in hepatoma cell lines, and miR-222-3p levels were deregulated in HBV-positive HCC cell lines consistent with in tissue (Supplementary Fig. 3E).

Moreover, we detected miR-222-3p and ETS1 mRNA levels in 20 pairs of HBV-HCC cancer and paracancerous tissues to find ETS1 mRNA levels were negatively correlated with the miR-222-3p levels in both HBV-positive HCC tissues by cox regression (Fig. 7E). Ectopic over-expression of HBx downregulated miR-222-3p levels in HBV-negative HepG2 and Huh7 cells (Fig. 7F). It was shown that miR-222-3p could be dysregulated by HBx protein.

MiRNAs are well-recognized to regulate gene expression by binding to the 3’UTR of gene promoters [21, 26]. Therefore, we concluded that miR-222-3p might regulate ETS1 mRNA levels by binding to the ETS1 promoter region. To verify this hypothesis, we first studied the mechanism that how miR-222-3p regulates ETS1. ETS1 promoter binding sites were predicted by TargetScan database (version 7.2), which were used to design and construct wild-type and mutant luciferase plasmids with different loci (Fig. 7G). The dual-luciferase reporter assay showed that miR-222-3p expression significantly attenuated the luciferase activity of the reporter with wild-type ETS1, but not that of the mutant-1 reporter (Fig. 7H). Next, we examined the changes in ETS1 expression levels in HepG2.2.15 and HepAD38 (-) cell lines after transient transfection of miR-222-3p mimic and inhibitor. The qRT-PCR results showed that in both cell lines, miR-222-3p inhibitor upregulated, while mimic significantly inhibited ETS1 expression (Fig. 7I). Western blot analysis revealed the same results (Fig. 7J).

Furthermore, immunoprecipitation with western blot analysis was used to demonstrate that miR-222-3p regulates DHX9 ubiquitylation by mediating UHRF2 S643 phosphorylation. The ubiquitylation immunoprecipitation results revealed that the effect of miR-222-3p on DHX9 ubiquitylation was more significant at elevated UHRF2 levels and that inhibition of DHX9 ubiquitylation upon miR-222-3p downregulation could be reversed by overexpression of S643-mutated UHRF2. Western blot analysis illustrated this conclusion (Fig. 7K). Taken together, we conclude that HBx downregulates miR-222-3p to affect the phosphorylation of UHRF2 by ETS1-CDK2. After determining the regulatory role of miR-222-3p on ETS1, we further verified that miR-222-3p affects the phosphorylation of UHRF2 at serine 643. Western blot assay was used to analyze the levels of downstream proteins in two HBV-positive cell lines, HepG2.2.15 and HepAD38 (-) post miR-222-3p mimic or inhibitor treatment. The results showed that CDK2, phosphorylated S643 UHRF2 and DHX9 protein levels were all affected by miR-222-3p (Fig. 7L), which was in keeping with the results following miR-222-3p regulation of ETS1 levels. In addition, we reverted the miR-222-3p inhibitor induced upregulation of UHRF2 phosphorylation by knocking down CDK2 levels (Fig. 7M). Together, these results confirm that HBx phosphorylates UHRF2 via the miR-222-3p-ETS1-CDK2 axis.

### HBx induced miR-222-3p inhibition increases proliferation, migration and invasion of HBV-positive HCC cells

**Fig. 8:**
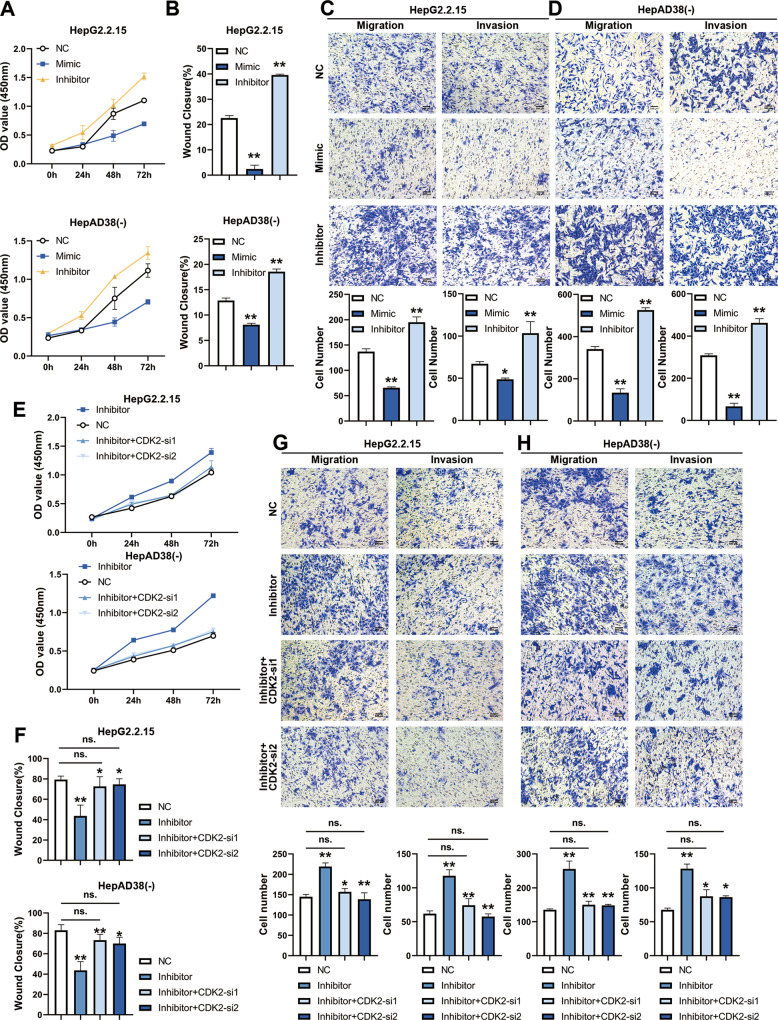
MiR-222-3p inhibition increases proliferation, migration and invasion of HBV-positive HCC cells. HBV-positive HepG2.2.15 and HepAD38(-) cell were infected with ectopic mimic or antagonism (inhibitor) of cellular miR-222-3p. **A** miR-222-3p a promoted cell proliferation in indicated cell lines. CCK-8 assay (*n* = 3) was used to observe cell proliferation ability in HepG2.2.15 (top panel) and HepAD38(-) (bottom panel). **B** miR-222-3p promoted cell migration in indicated cell lines. Wound healing assays (*n* = 3) were used to observe cell migration ability in HepG2.2.15 (top panel) and HepAD38(-) (bottom panel). **C**, **D** miR-222-3p enhanced cell migration and invasion of indicated cell lines. Transwell migration and invasion assays (*n* = 3) was used to observe both migration and invasion ability in HepG2.2.15 (**C**) and HepAD38(-) (**D**). HBV-positive HepG2.2.15 and HepAD38(-) cells were infected with antagonism (inhibitor) of cellular miR-222-3p or miR-222-3p inhibitor and CDK2 siRNAs. Cells were harvested for following assays 48 h post-transfected. **E** CDK2 siRNAs partially restored the proliferative capacity of HBV-positive HCC cells by rescuing the upregulation of CDK2 protein expression caused by miR-222-3p inhibitor. CCK-8 assay (*n* = 3) was used to observe cell proliferation ability in HepG2.2.15 (top panel) and HepAD38(-) (bottom panel). **F** CDK2 siRNAs partially restored the migratory capacity of HBV-positive HCC cells by rescuing the upregulation of CDK2 protein expression caused by miR-222-3p inhibitor. Wound healing assays (*n* = 3) were used to observe cell migration ability in HepG2.2.15 (top panel) and HepAD38(-) (bottom panel). **G**, **H** CDK2 siRNAs partially restored the ability of HBV-positive HCC cells migration and invasion by rescuing the upregulation of CDK2 protein expression caused by miR-222-3p inhibitor. Transwell migration and invasion assays (*n* = 3) was used to observe both migration and invasion ability in HepG2.2.15 (**G**) and HepAD38(-) (**H**). Statistical comparisons were using Welch’s two sample *t*-test. ns. not significant; *, *p* < 0.05; **, *p* < 0.01.

Cck-8 assay was performed on miR-222-3p mimic or inhibitor and control clones of HepG2.2.15 and HepAD38(-) cells. The results suggested that miR-222-3p mimics suppressed cell proliferation, whereas inhibitors promoted cell proliferation (Fig. 8A). The assessment of the miR-222-3p regulation of cell migration was performed by wound healing assay. Expectedly, the miR-222-3p inhibitor significantly increased migration in these HBV-positive HCC cells, while decreased migration was observed in the group treated with mimics (Fig. 8B). In addition, the transwell migration and invasion assay revealed that the miR-222-3p inhibitor significantly promoted migration and invasion in HBV-positive HCC cells (Fig. 8C, D).

Meanwhile, to observe the effect of changes in UHRF2 phosphorylation levels regulated by miR-222-3p-ETS1-CDK2 axis on cell function. We performed CDK2 knockdown to rescue the UHRF2 phosphorylation promoted by miR-222-3p inhibitor and observed the changes in proliferation, migration and invasion functions of HBV-positive HCC cells among the NC, positive and rescue groups. Results of cck-8 assay (Fig. 8E) suggest that reduced of UHRF2 phosphorylation caused by CDK2 knockdown rescued the promotion of cell proliferation. The wound healing assay (Fig. 8F) and the Transwell migration (Fig. 8G) and invasion (Fig. 8H) assay demonstrated that the migration and invasion functions of HCC cells could also be restored by CDK2 knockdown. Collectively, miR-222-3p-ETS1-CDK2-UHRF2 axis increases malignant phenotypes in HCC cells.

## Discussion

This study investigated the role of the UHRF2 phosphorylation-ubiquitylation switch in the process of HBV replication and HBV-associated HCC disease. A mechanism by which UHRF2 affects the poor prognosis of HBV-associated HCC patients has been elucidated.

HBV is considered to be one of the main causative factors in the development of HCC and is highly correlated with the survival and prognosis of HCC patients. HBV-associated HCC accounts for approximately half of all HCC cases [7]. However, the mechanism of HBV-associated HCC formation is unclear. There is an important link between the continued replication of HBV in humans and the malignant phenotypes of HCC [27, 28]. HBV integrates its genome into the host genome after infection and continues to replicate in order to infect other liver cells [8]. Finding target molecules affecting both HBV and HCC may be a viable strategy to overcome the two closely related diseases.

UHRF2 is a ubiquitin-protein ligase E3 and is involved in the development of many cancers. Increased expression of UHRF2 in esophageal squamous cell carcinoma [12, 14] is negatively associated with cancer progression, whereas the reverse was true in non-small cell lung cancer [11], colorectal cancer [29], gastric cancer, and intrahepatic cholangiocarcinoma. In previous studies, UHRF2 was identified as an oncogene and was associated with poor prognosis, immune infiltration and metastasis. This study clarified that phosphorylation of UHRF2 is an important factor affecting oncogenicity. Phosphorylation of UHRF2 blocked its ubiquitin-protein ligase E3 function and inhibited DHX9 degradation. Indirectly, it promoted HBV replication and HBV-associated HCC development by upregulating DHX9 protein levels. This may be related to the altered structure of UHRF2 protein after phosphorylation. The UHRF2 protein levels were upregulated in HBV-positive HCC cancer tissues compared to para-cancerous tissues (Fig. 1A, Supplementary Fig. 1B). However, due to auto-ubiquitylation, the half-life of UHRF2 protein is short, and low protein levels were found in most tissues. UHRF2 protein levels were only stably high in HBV-positive HCC cells and tissues. Therefore, we hypothesized that the presence of the HBV virus alters its stability. HBx upregulates its protein stability by promoting UHRF2 phosphorylation. However, UHRF2 does not directly affect HBV-related expression [30], suggesting that other factors mediate the interaction between UHRF2 and HBV. Subsequently, DHX9 was identified via mass spectrometry to bound with UHRF2 [21]. Recent studies strongly link the connection between DHX9 and HBV-related disease processes [31, 32]. DHX9 is a nuclear DNA/RNA helicase enzyme and resolvase. Mechanistically, it affects DNA, RNA and complexes, also impacting various processes such as DNA replication, transcription, RNA translocation, translation, micro-RNA and circular RNA processing [33–36]. DHX9 may neutralize the immediate threat posed by transposon insertions, allowing them to evolve as post-transcriptional regulators of gene expression [37]. Functionally, DHX9 is involved in the expression and nuclear export of retroviral RNAs [19, 38–40] and is dysregulated in various cancers [32, 41–45]. Since our previous mass spectrometry results identified the binding of UHRF2 to DHX9, this study demonstrated that UHRF2 acts as a ubiquitin-protein ligase E3 to promote DHX9 degradation. However, this is inconsistent with the high expression of DHX9 in HBV-associated HCC. Our previous study revealed that phosphorylation at serine 643 exerted an important effect on the proto-oncogenic function of UHRF2. Therefore, only 643 serine phosphorylated UHRF2 have a different effect on DHX9 ubiquitylation and degradation, whereas the S643 mutant UHRF2 does not. Furthermore, the HBV viral protein HBx affects UHRF2 phosphorylation by ectopic overexpression of HBx in HBV-negative HepG2 cells, suppressing DHX9 expression in HBV-positive HCC and promoting HBV disease progression (Fig. 5C–F). At the same time, the literature reports downregulation of DHX9 expression in non-HBV hepatocellular carcinoma [45], indirectly affirming our conclusion. In conclusion, upregulated UHRF2 protein levels by its phosphorylation at serine 643 suppresses the ubiquitin-protein ligase E3 activity. UHRF2 regulates HBV replication by inhibiting DHX9 degradation and worsens clinical outcomes by promoting the development of HCC (Fig. 6).

In addition, the HBV viral protein HBx was characterized as a critical role in the regulation of UHRF2 phosphorylation. Accordingly, the mechanism was investigated, thereby facilitating the positive feedback between HBV replication and HCC carcinogenesis. HBx protein, one of the transcription products of the HBV genome, is recognized to play an consequential role in the HBV-related disease process. HBx is thought to regulate host gene expression mainly through post-transacting [24, 25], and UHRF2 does not bind directly to HBx. Recent studies have shown that HBx affects cellular function by dysregulating the expression of multiple non-coding RNAs, especially miRNAs [46–49]. Therefore, we screened miRNAs affecting UHRF2 phosphorylation that may be regulated by HBx and identified miR-222-3p as a potential candidate. Significantly downregulated miR-222-3p levels were observed in HBV-associated HCC (Fig. 7F). Inhibiting miR-222-3p expression resulted in upregulation of ETS1, which was proved to be associated with UHRF2 phosphorylation. The relationship between miR-222-3p and ETS1 was elucidated by qRT-PCR, Western blot and dual luciferase assay (Fig. 7G–J), clarifying the role of HBx in the regulation of UHRF2 phosphorylation (Fig. 7K–M). These results demonstrate that miR-222-3p is indeed an essential link in the process of HBx-UHRF2 regulation of DHX9 ubiquitylation and degradation.

Intriguingly, DHX9 may affect miRNA shearing through its own miRNA splicing activity [49]. By analyzing the primary miR-222-3p RNA levels versus mature miR-222-3p RNA levels after overexpression and knockdown of DHX9 in HepG2.2.15 and HepAD38(-) cells, we found that DHX9 expression changes only affected miR-222-3p levels, but did not affect primary miR-222-3p levels (Supplementary Fig. 4A, B). These findings may not be a coincidence, and miR-222-3p may be only one of many miRNAs regulated by DHX9. DHX9 may be responsible for the general dysregulation of miRNA levels in HBV host cells in a similar manner.

**Fig. 9:**
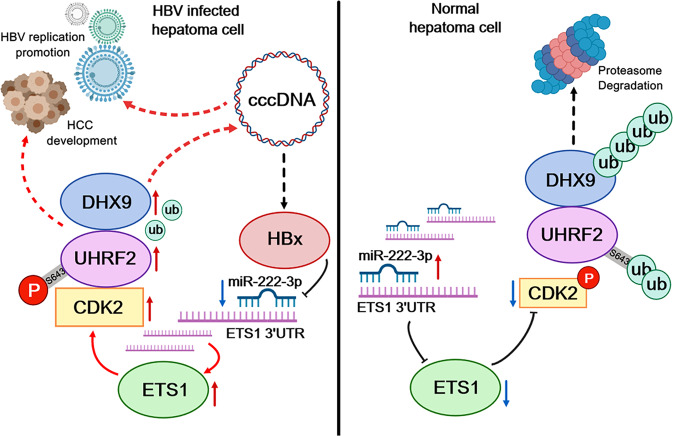
HBx promotes viral replication and HCC development by influencing UHRF2 phosphorylation in HBV-positive HCC. Phosphorylation of UHRF2 acts as a “switch” in the disease process, allowing UHRF2 to play a bi-faceted role in cancer. This study focused on the mechanism through which the viral protein HBx promotes viral replication and HCC development by influencing UHRF2 phosphorylation in HBV-positive HCC.

In summary, the positive feedback regulation of HBV-UHRF2-DHX9 on the development of HBV-associated HCC has been reported for the first time, improving our knowledge of the mechanism of co-progression between HBV and HCC. We suggest that phosphorylation of UHRF2 acts as a “switch” in the disease process, allowing UHRF2 to play a bi-faceted role in cancer. This study focused on the mechanism through which the viral protein HBx promotes viral replication and HCC development by influencing UHRF2 phosphorylation in HBV-positive HCC. The results confirmed that UHRF2 phosphorylation plays a critical role in the HBV disease process and implied that UHRF2 could be a new regulator and a potential prognostic indicator of adverse clinical outcomes for HBV-associated HCC (Fig. 9).

## Materials and methods

### Patients and samples

The use of human tissues in this study was approved by the Ethics Review Committee of Chongqing Medical University (Chongqing, China), numbered as 2019005. All the samples were obtained from resected cancer and para-cancerous tissues of HBV-positive patients who underwent liver resection at the First Hospital of Chongqing Medical University between September 2019 and May 2021 with their informed consent. The baseline information related to patient specimens was listed in Supplementary Table 1.

### Cell transfection and lentivirus infection

The cell lines used in this experiment have been identified by STR profiling and were free of mycoplasma contamination. Plasmids, miR-222-3p mimic, cellular miR-222-3p inhibitor (or antagonism), and small interfering RNAs (siRNA) were transfected by Lipofectamine 2000 (Invitrogen, Carlsbad, CA) or Advanced DNA RNA Transfection Reagent (ZetaLife, USA) according to the reagent instructions.

Ectopic overexpression lentivirus infected cells for at least 48 h, followed by puromycin and blasticdin selection according to the manufacturer’s protocol.

Detailed information of plasmids and lentivirus in this study was listed in the Supplementary Table 2. Sequence of miR-222-3p mimic, inhibitor, and siRNAs were available in Supplementary Table 3.

### Quantitive reverse transcription PCR (qRT-PCR)

All primers used in qRT-PCR experiment were available in Supplementary Table 5.

### HBV cccDNA quantitation

A previous study described the extraction of HBV cccDNA [50–52]. Cells were lysed in lysis buffer containing 50 mM Tris-HCl, pH 7.4, 1 mM EDTA, and 1% NP-40 supplemented with proteinase inhibitor cocktail (Topscience, China) for 10 min. After centrifugation, the precipitation was resuspended in lysis buffer B (10 mM Tris-HCL, 10 mM EDTA, 150 mM NaCl, 0.5% SDS, and 0.5 mg/ml protein K) and lysed for 16 h. Subsequently phenol-chloroform extraction and ethanol precipitation. The extracted DNA was treated at 37 °C with Plasmid-Safe DNase I (Takara, China) and further treated at 70 °C to inactive the Plasmid-Safe™ ATP-Dependent DNase for 30 min (Lucigen, USA) before analyzed by qPCR with primers supplied in Supplementary Table 5.

### Western blot

Whole cell extract was isolated from 48 h post-transfection cells with RIPA lysis buffer (Beyotime Biotechnology, China) with proteasome inhibitor cocktail EDTA-free (Roche, Germany) and phosphatase inhibitor cocktail I/II (Topscience, Shanghai, China). After protein concentrations determinated by BCA protein assay Kit (Beyotime Biotechnology, China), the proteins were separated by SDS-PAGE, followed by western blotting with indicated antibodies. All the primary antibodies were listed in Supplementary Table 4. HRP conjugated Goat anti-Mouse/Rabbit antibody were used as secondary antibody (Boster, Wuhan, China) for chemiluminescence. Eventually, bands were visualized using SuperSignal™ West Atto Ultimate Sensitivity Substrate (ThermoFisher Scientific, USA) or Immobilob™ Western Chemiluminescent HRP Substrate (Millipore, USA). Antibodies applied in this study were listed in Supplementary Table 4. Uncropped western blot strips are provided in the supplementary material.

### Immunoprecipitation and Co-immunoprecipitation (Co-IP)

Cells were lysed with NP40 (Beyotime Biotechnology, China), and supplemented with proteasome inhibitor cocktail EDTA-free (Roche, Germany) and phosphatase inhibitor cocktail I/II (Topscience, Shanghai, China) 72 h–96 h post-transfection. WCE were collected after centrifugation at 14,000 × *g*. After protein concentration quantified, WCEs at equal concentration and volume were pre-cleaned by IgG of the same species as the indicated primary antibody and protein A/G agarose. The supernatant was immunoprecipitated over night at 4 °C with indicated primary antibodies and protein for following assays. The precipitated protein complexes were washed using NP40 lysis buffer at least five times before being separated on SDS-PAGE and immunoblotting by the indicated antibodies.

For ubiquitylation immunoprecipitation, cells were transfected by HA tagged ubiquitin (HA-ub) plasmids together with the other indicated plasmids. Before collecting the cell lysate, cells were treated with 20 μmol/L of MG-132 (Topscience, Shanghai, China) for at least 6 h. The following assays were performed as previous described. Antibodies applied in this study were listed in Supplementary Table 4.

### In vivo subcutaneous xenograft tumor

In xenograft experiments, we injected 1 × 10^7^ cells/mouse into randomly grouped 4-week-old male athymic BALB/c nude mice (Vital River Laboratories, USA). The control and treatment groups were allocated blinding and randomized. Twenty days after the operation, subcutaneous tumors were removed and preserved after humane execution of the mice. Tumor weights and volumes were determined immediately.

### Duel luciferase reporter gene experiment

Plasmids used in this assay were listed in the Supplementary Table 2.

### Statistical analyses

Bar graphs present the average ± SD of the results for the experiments, which were independently repeated at least three times. All statistical analyses were given a performance by GraphPad Prism 8.0.2 (GraphPad Software Inc., Chicago, IL). Student’s *t* test, paired *t*-test, Wilcox test, Welch’s two-sample *t*-test and Spearman’s correlation test were used as appropriate. The variance is similar between the groups that are being statistically compared. The survival curve was evaluated by the Kaplan–Meier method. *P* values <0.05 (*, *p* < 0.05; **, *p* < 0.01) were considered to be statistically significant throughout this study.

## Supplementary information

Supplementary Table 1

Supplementary Table 2

Supplementary Table 3

Supplementary Table 4

Supplementary Table 5

Supplementary Figure Legends

Supplementary Figure 1

Supplementary Figure 2

Supplementary Figure 3

Supplementary Figure 4

Original uncropped western blot strips

## Data Availability

All datasets used in this study have been analyzed during the current study are available from the corresponding author on reasonable request.
