# Supplementary material for: Phosphorylation of UHRF2 affects malignant phenotypes of HCC and HBV replication by blocking DHX9 ubiquitylation
Source: Cell Death Discov. 2023 Jan 24;9:27. doi: 10.1038/s41420-023-01323-2 (PMC9871042; doi:10.1038/s41420-023-01323-2)
Supplement: Supplementary file 6 — Supplementary Figure Legends [file 41420_2023_1323_MOESM6_ESM.docx]

**Supplementary Figure Legends**

**Supplementary Figure 1. UHRF2 mRNA level upregulates in HCC and HBV-positive HCC cancers.**

1. UHRF2 mRNA upregulated in HCC. Expression of UHRF2 mRNA level in HCC specimens (n=371) and non-tumor tissues (n=50) were from TCGA database. The differential analysis is based on TCGA-LIHC dataset versus TCGA normal + GTEx normal.
2. UHRF2 mRNA upregulated in HBV-associated HCC. Expression of UHRF2 mRNA level in HBV-positive HCC specimens (n=145) and non-tumor tissues (n=50) were from TCGA database. The differential analysis is based on HBV-positive data from TCGA-LIHC dataset versus TCGA normal + GTEx normal.

All the statistical comparisons were using Wilcox test. **, p<0.01.

**Supplementary Figure 2. Lentivirus, UHRF2 siRNA and overexpression efficiency validation.**

(A-B) UHRF2 siRNAs knockdown (B) and overexpression (C) efficiencies were detected in HepG2.2.15 cells. 48h post-transfected, UHRF2 mRNA levels were analyzed by qRT-PCR and western blot, n=3. Image J gray-scale scanning was used to obtain the gray values of UHRF2 bands. All the values were normalized to GAPDH (mRNAs) and β-actin (proteins).

(C) Stably overexpression Huh7 cell lines were successfully established. Huh7 cell were infected by indicated lentivirus. We cultured infected cells in puromycin (2ng/mL) and blasticidin (1ng/mL) containing medium for 2 weeks for following assays. UHRF2 (top panel) and HBx (bottom panel) mRNA expression were analyzed by qRT-PCR and values are normalized to GAPDH (n=3).

All the statistical comparisons were using Student t-test. ns., no significant; *, p<0.05 **; p<0.01.

**Supplementary Figure 3. miRNA screening and analysis of ETS1, miR-222-3p expression in HCC tissues and cell lines.**

1. B) Volcano plots (A) and heat map (B) were used to describe the differential expressed genes (DEGs).

(C) ETS1 mRNA upregulated in HBV-associated HCC. Expression of UHRF2 mRNA level in NBNC-HCC specimens (n=226) and HBV-HCC (n=145) were from TCGA database. miR-222-3p expression in hepatoma cell lines were analyzed by qRT-PCR. U6 were used for normalization.

(D-E) ETS1 mRNA (D) miR-222-3p (E) levels in hepatoma cell lines were analyzed by qRT-PCR (n=3). U6 were used for normalization.

For (C), statistical comparisons were using Wilcox test. *, p<0.05.

For (D-E), statistical comparisons were using Student t-test. **, p<0.01.

**Supplementary Figure 4. DHX9 regulates miR-222-3p expression by primary miR-222-3p splicing.**

1. DHX9 overexpression and knockout efficiency was confirmed in HepG2.2.15 and HepAD38(-) cell lines. qRT-PCR were used to analyze the level of DHX9 mRNA (n=3), and values normalized to GAPDH.
2. DHX9 level regulates mature miR-222-3p level but not primary miR-222-3p. Primary miR-222-3p level and mature miR-222-3p level were detected by qRT-PCR after transfection (n=3). The values normalized to GAPDH.

Statistical comparisons were using Student t-test. ns., not significant; *, p<0.05; **, p<0.01.
