# Supplementary figures and images for: Phosphorylation of UHRF2 affects malignant phenotypes of HCC and HBV replication by blocking DHX9 ubiquitylation
Source: Cell Death Discov. 2023 Jan 24;9:27. doi: 10.1038/s41420-023-01323-2 (PMC9871042; doi:10.1038/s41420-023-01323-2)

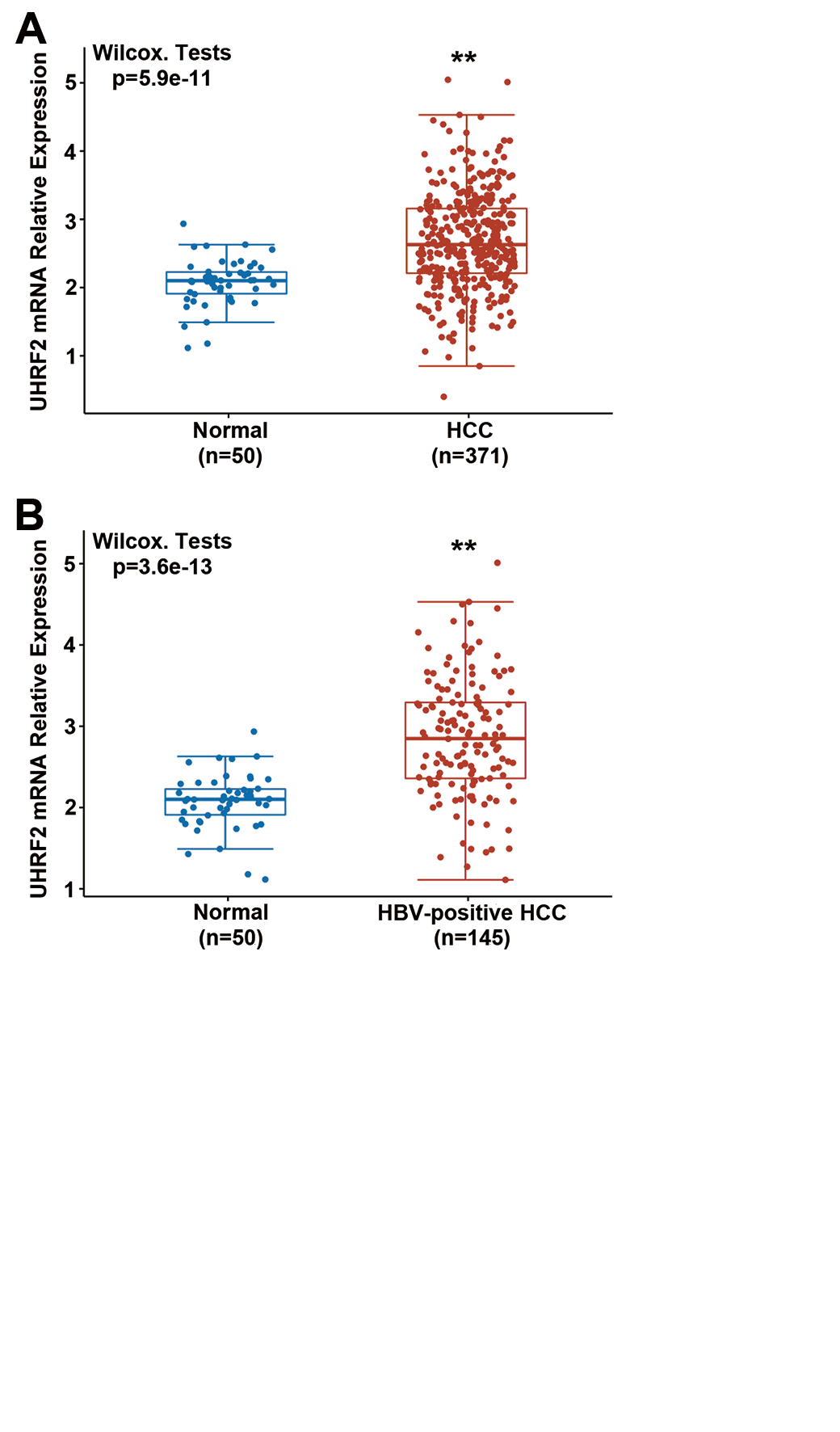

Supplement: Supplementary file 7 — Supplementary Figure 1 [file 41420_2023_1323_MOESM7_ESM.png]

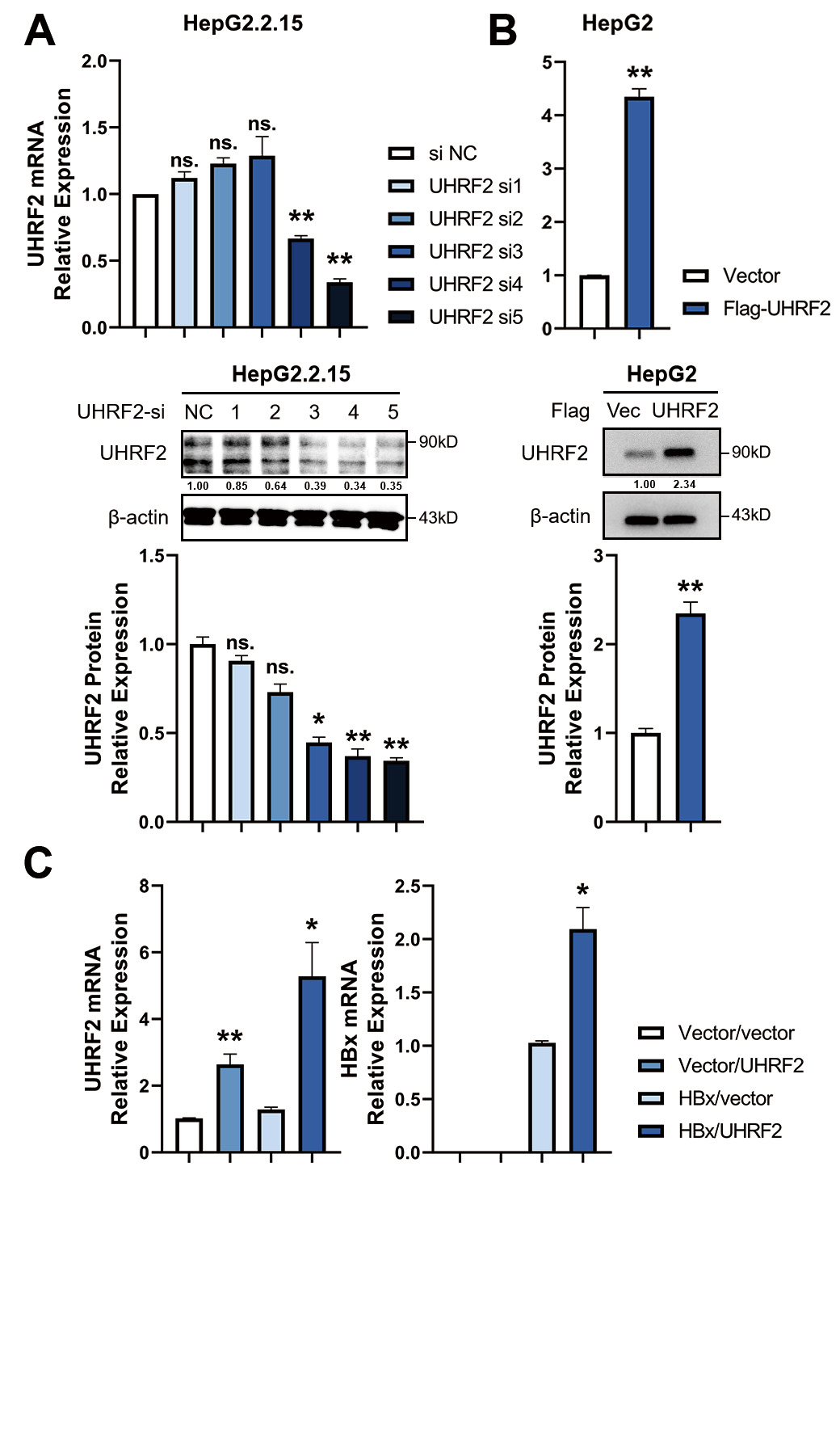

Supplement: Supplementary file 8 — Supplementary Figure 2 [file 41420_2023_1323_MOESM8_ESM.png]

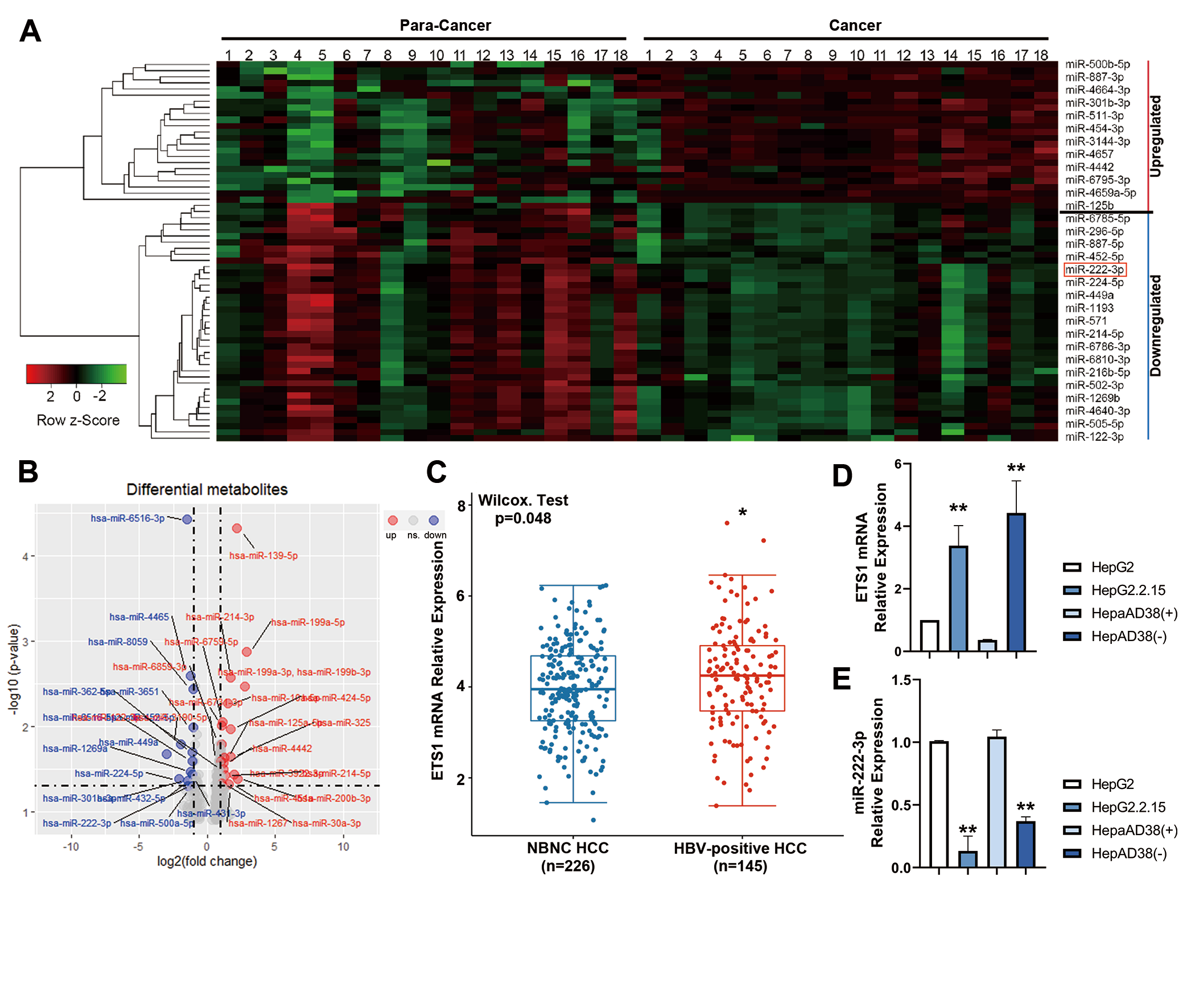

Supplement: Supplementary file 9 — Supplementary Figure 3 [file 41420_2023_1323_MOESM9_ESM.png]

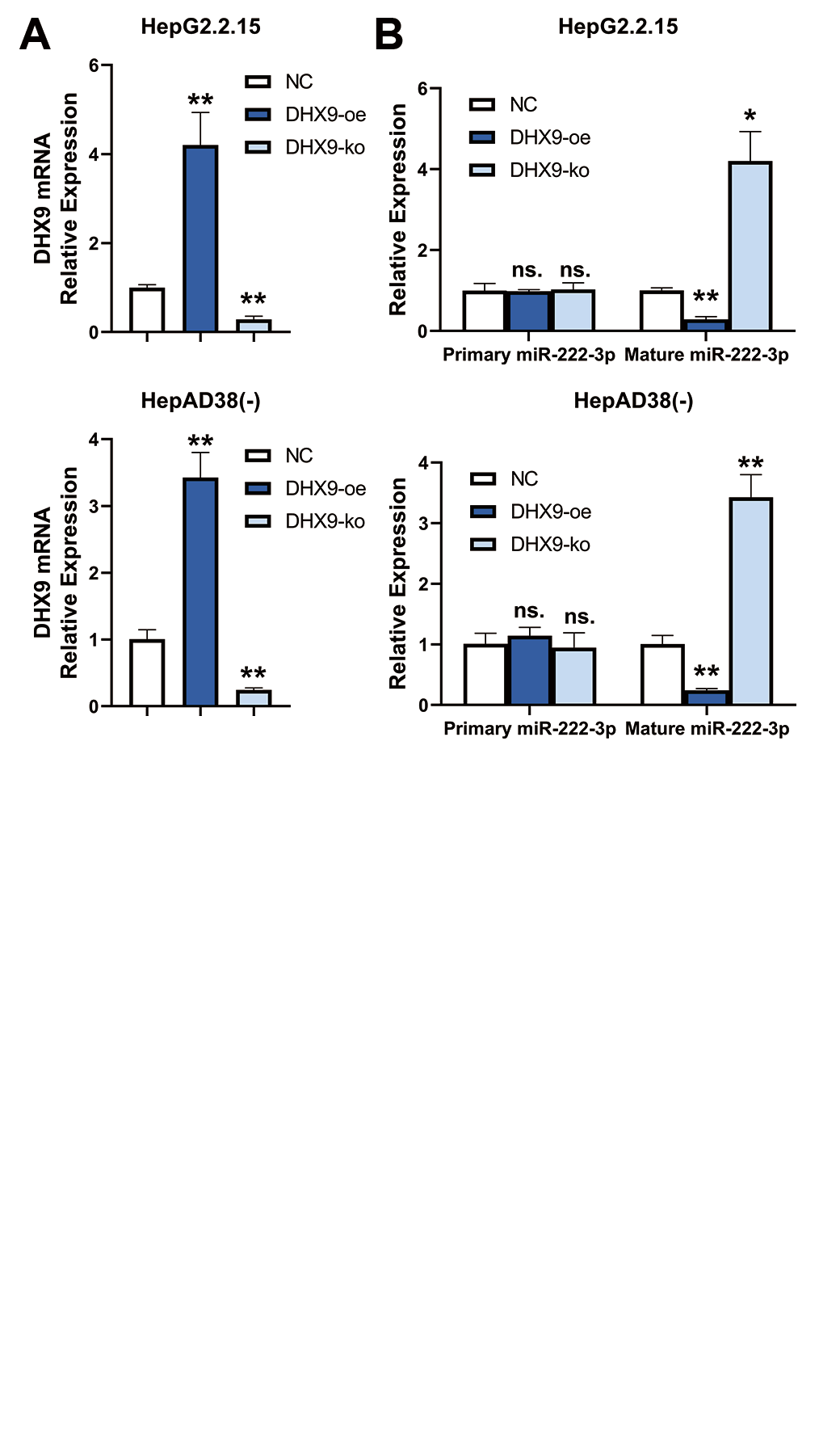

Supplement: Supplementary file 10 — Supplementary Figure 4 [file 41420_2023_1323_MOESM10_ESM.png]

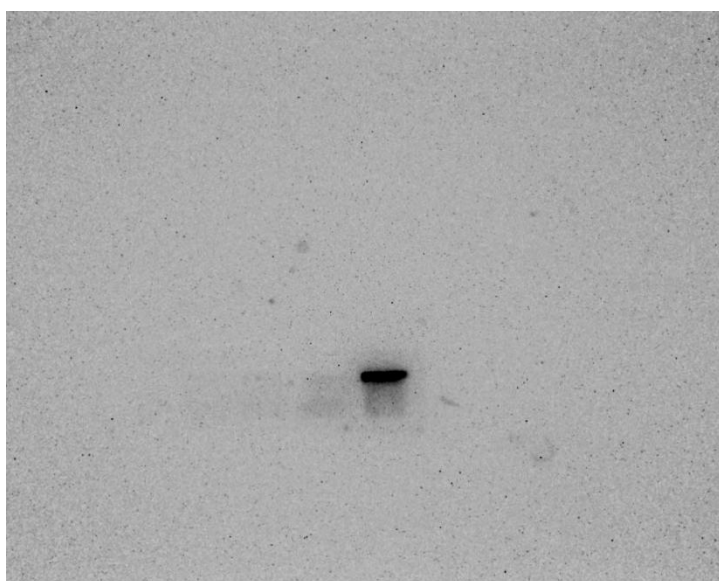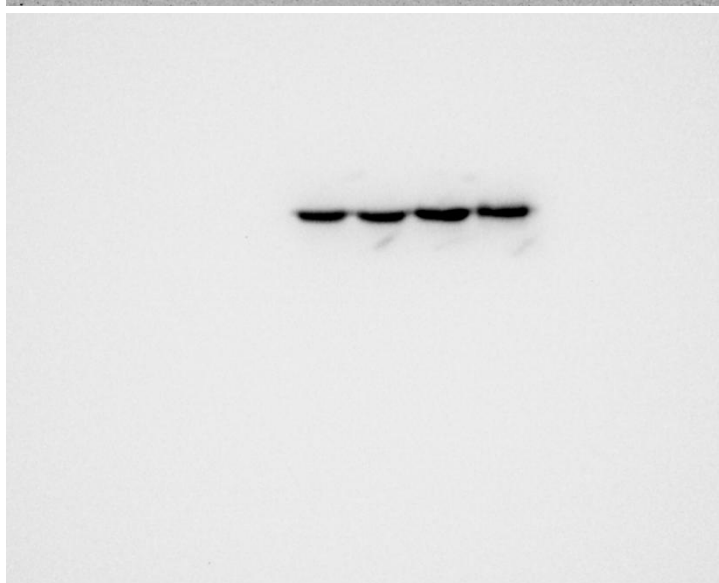

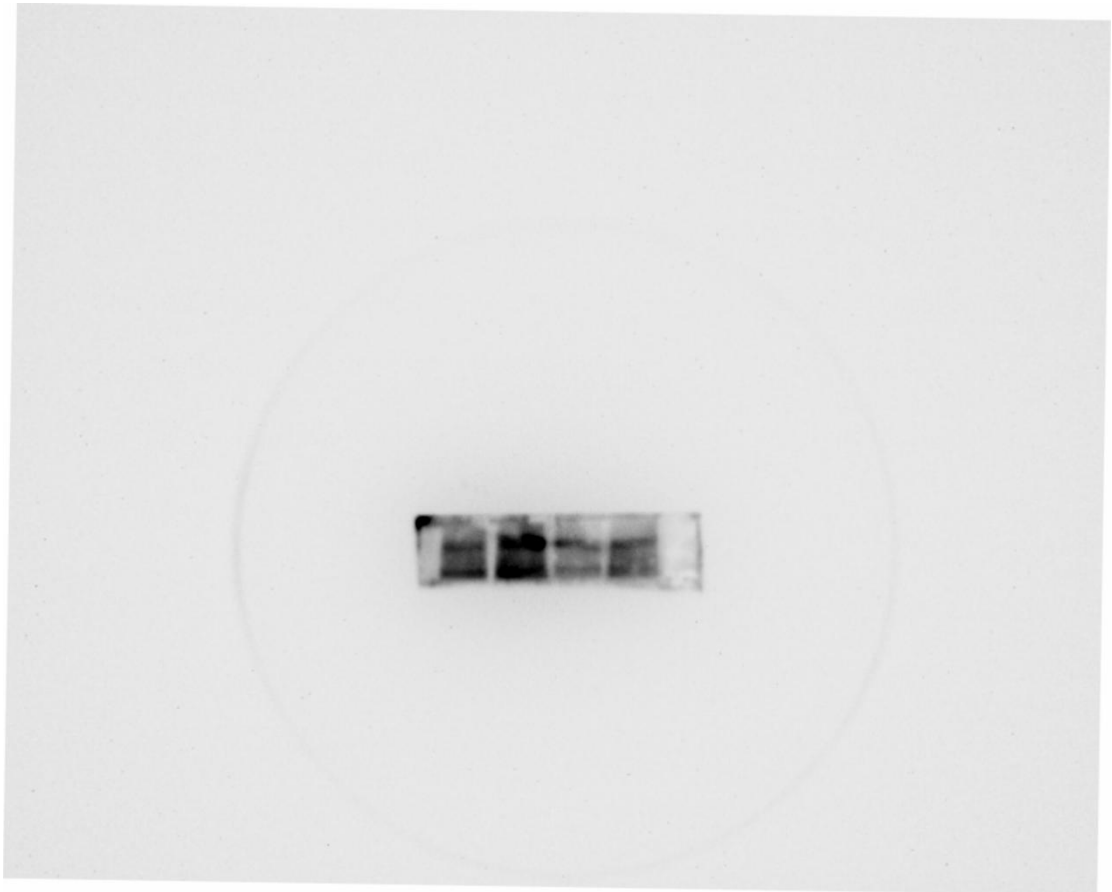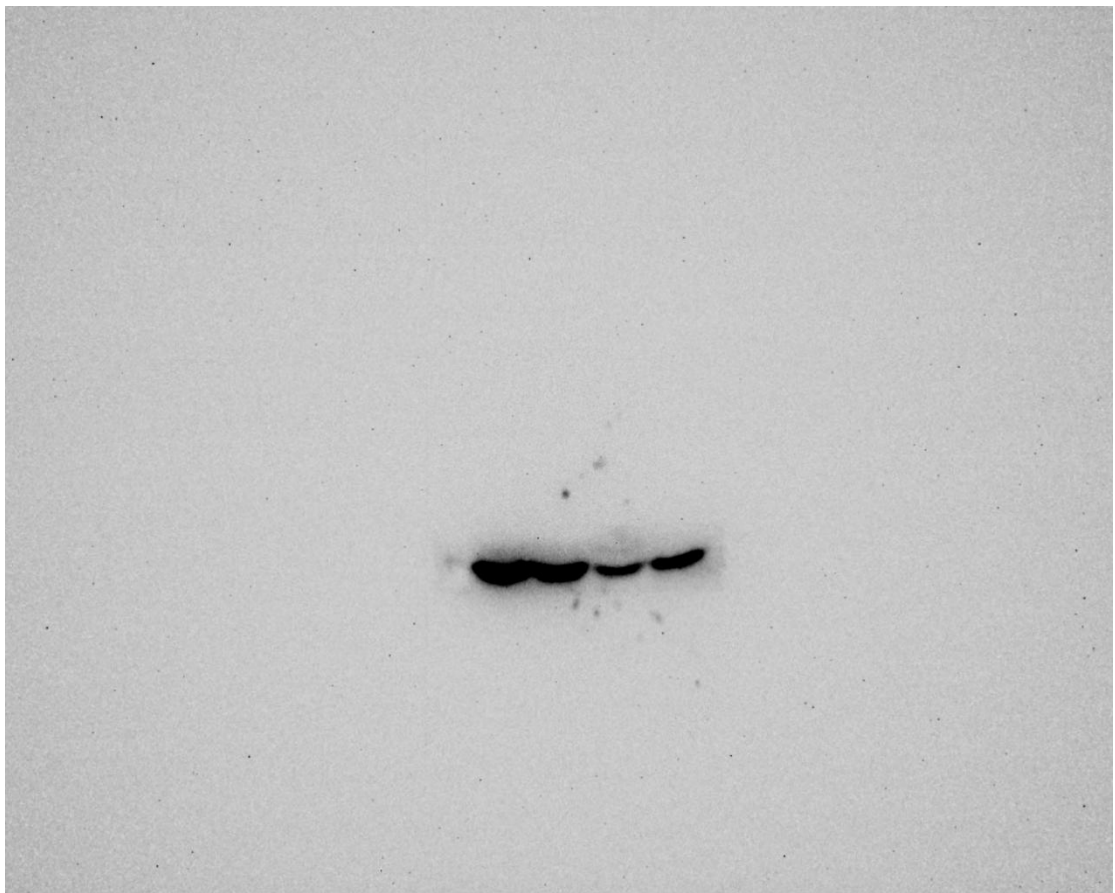

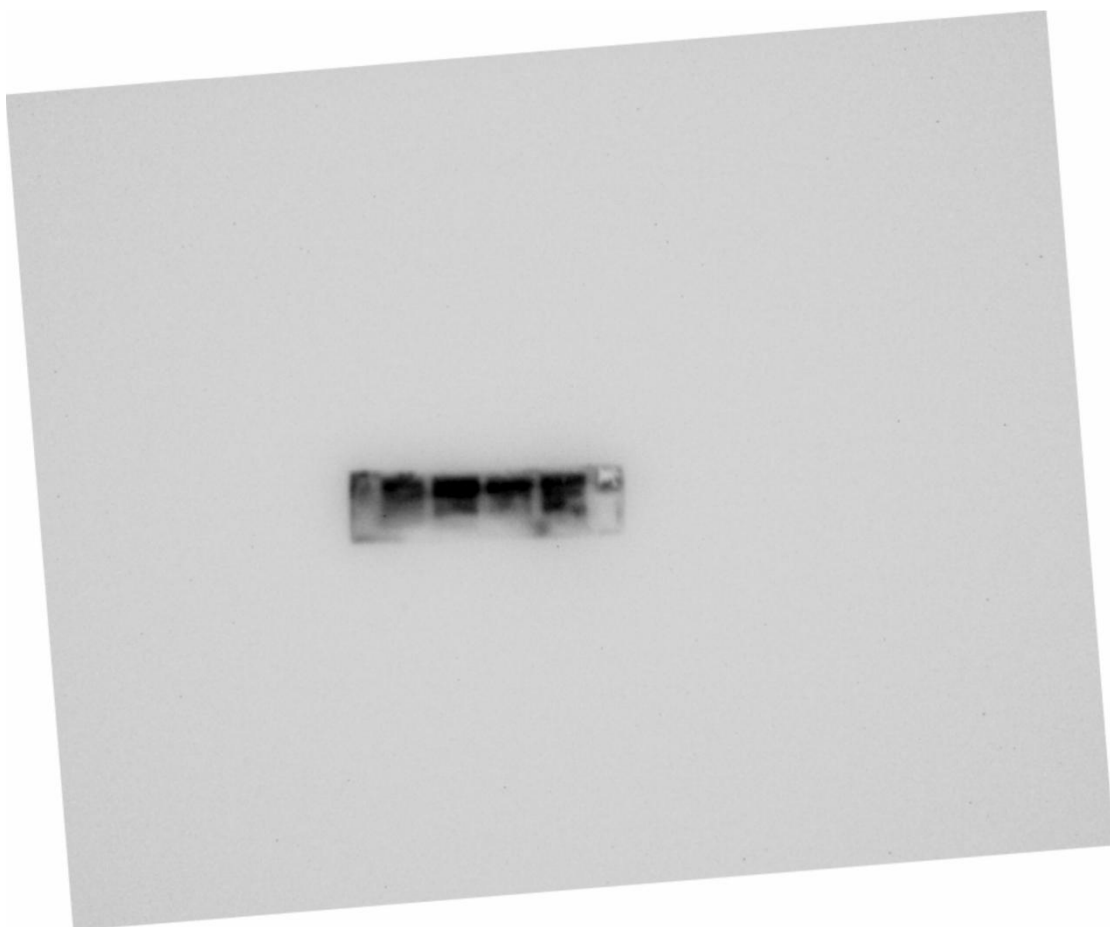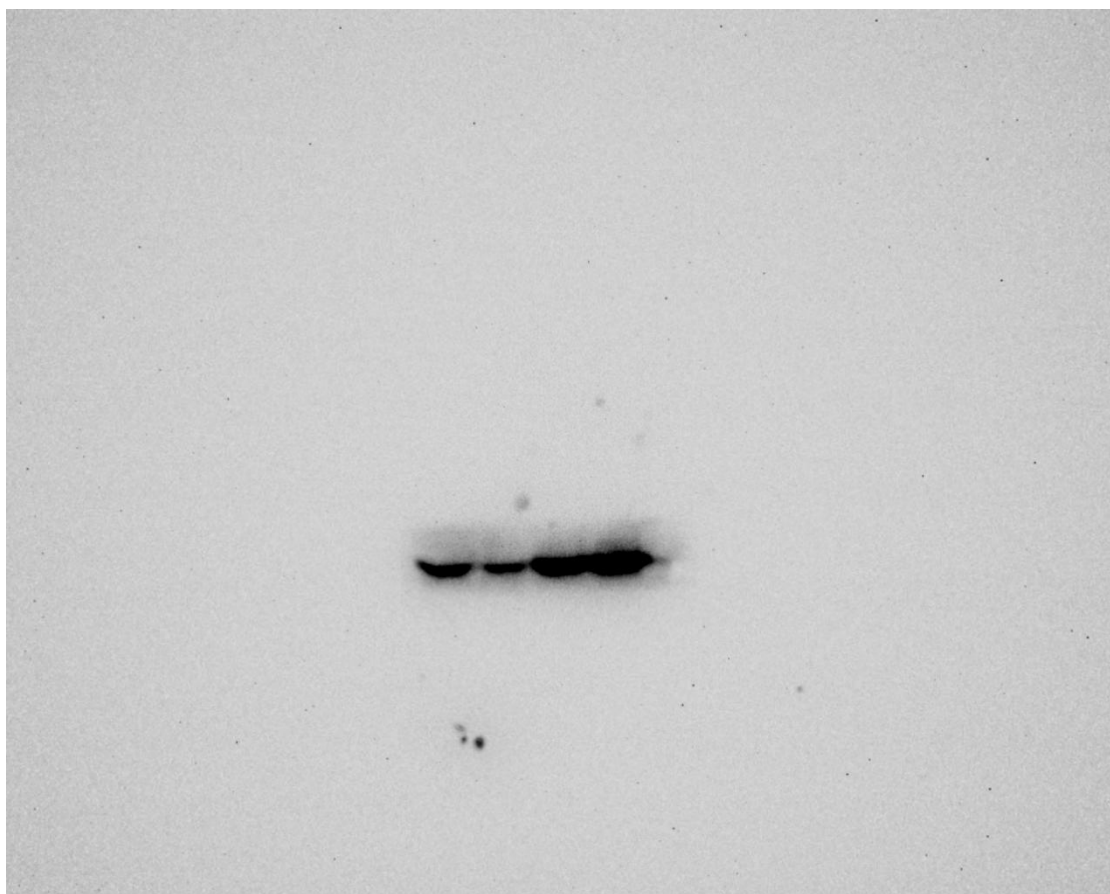

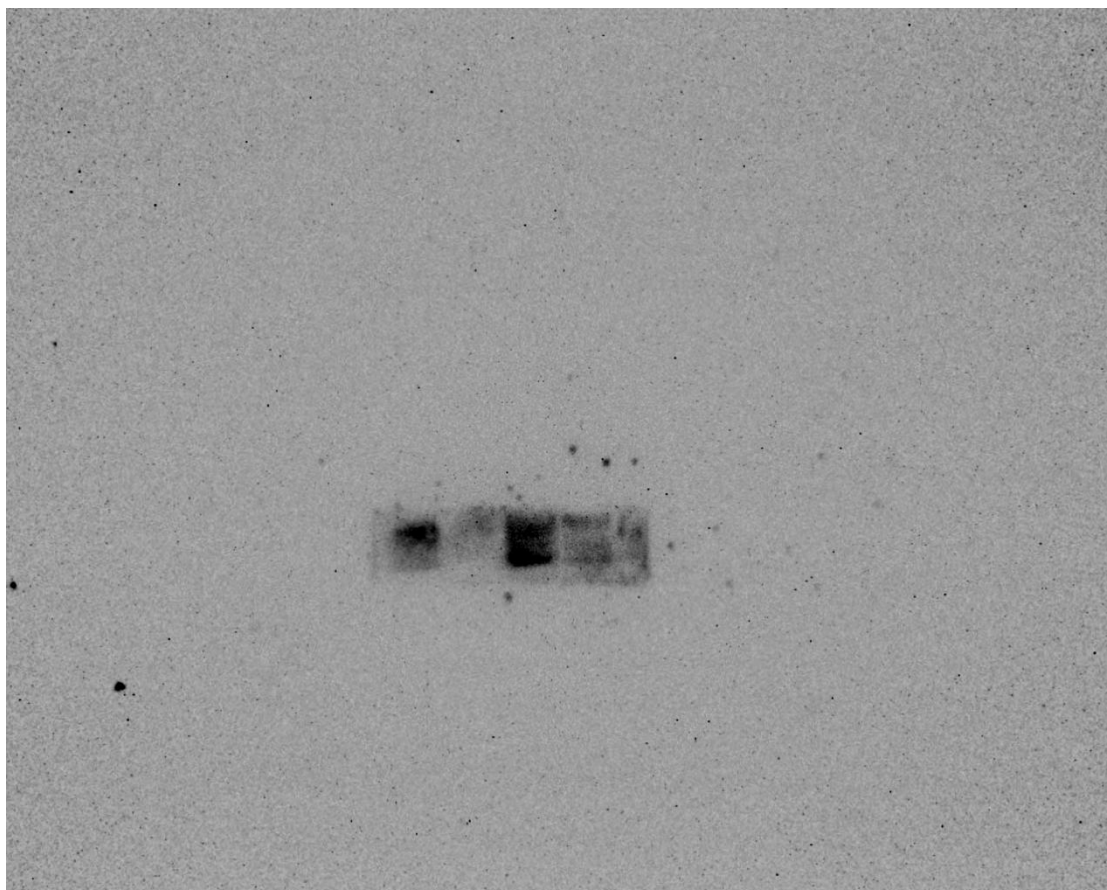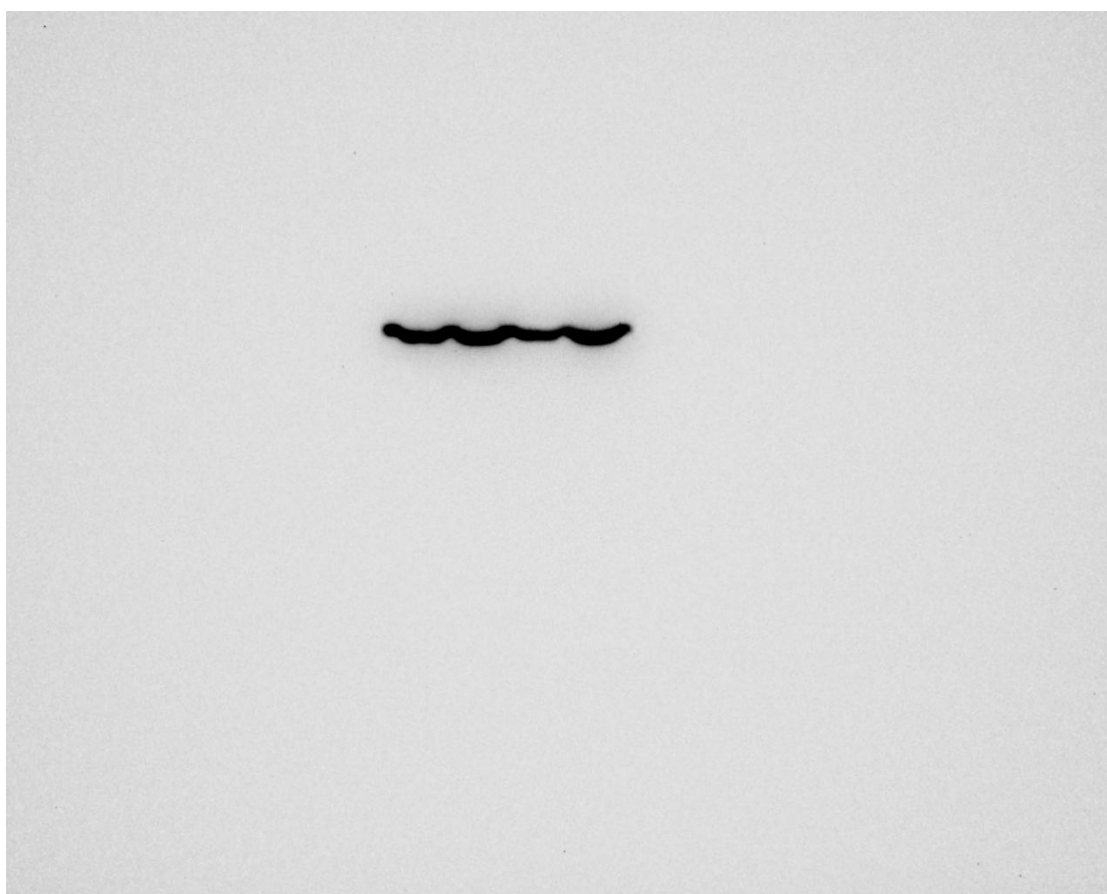

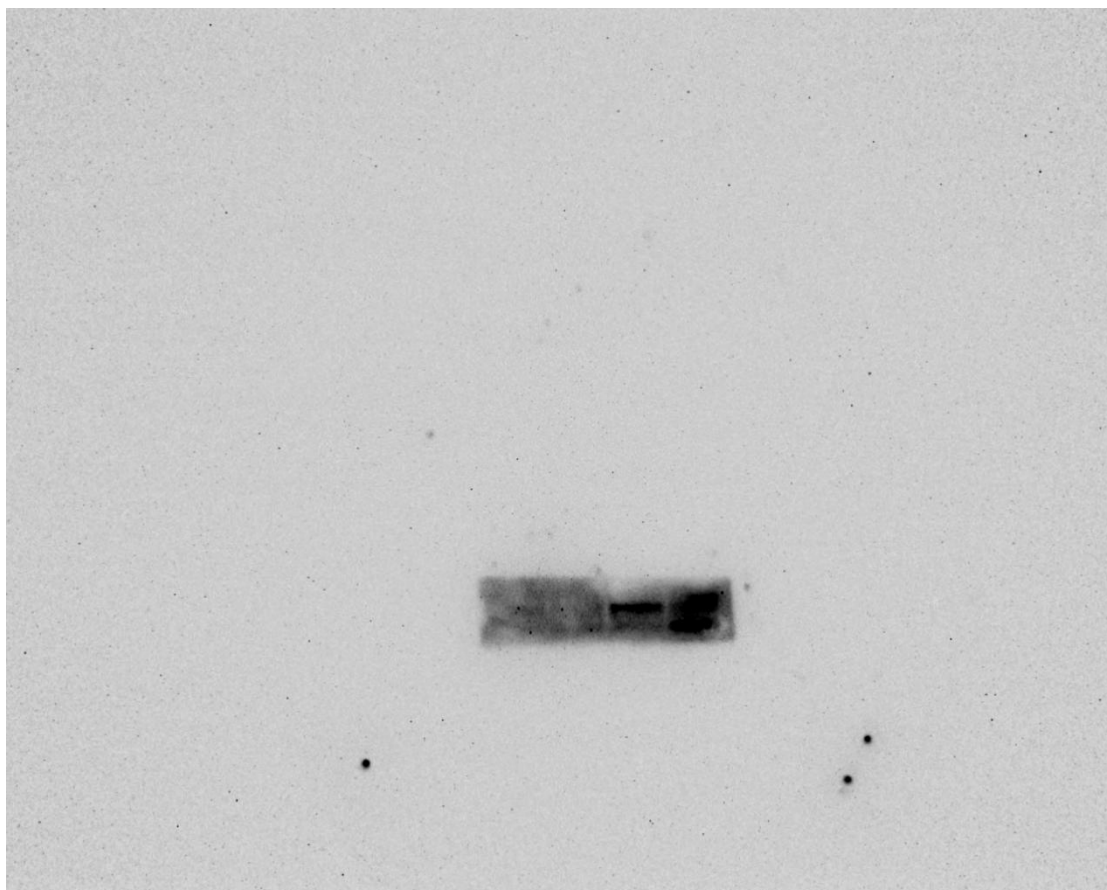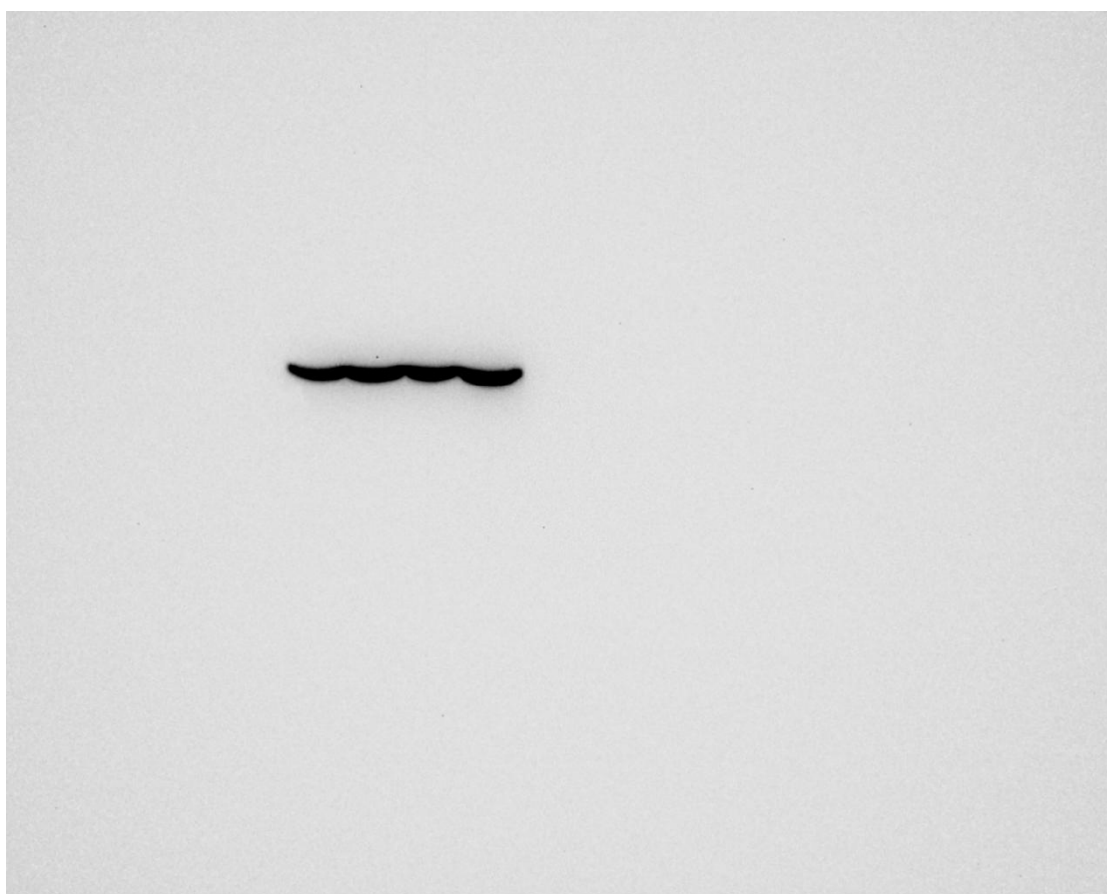

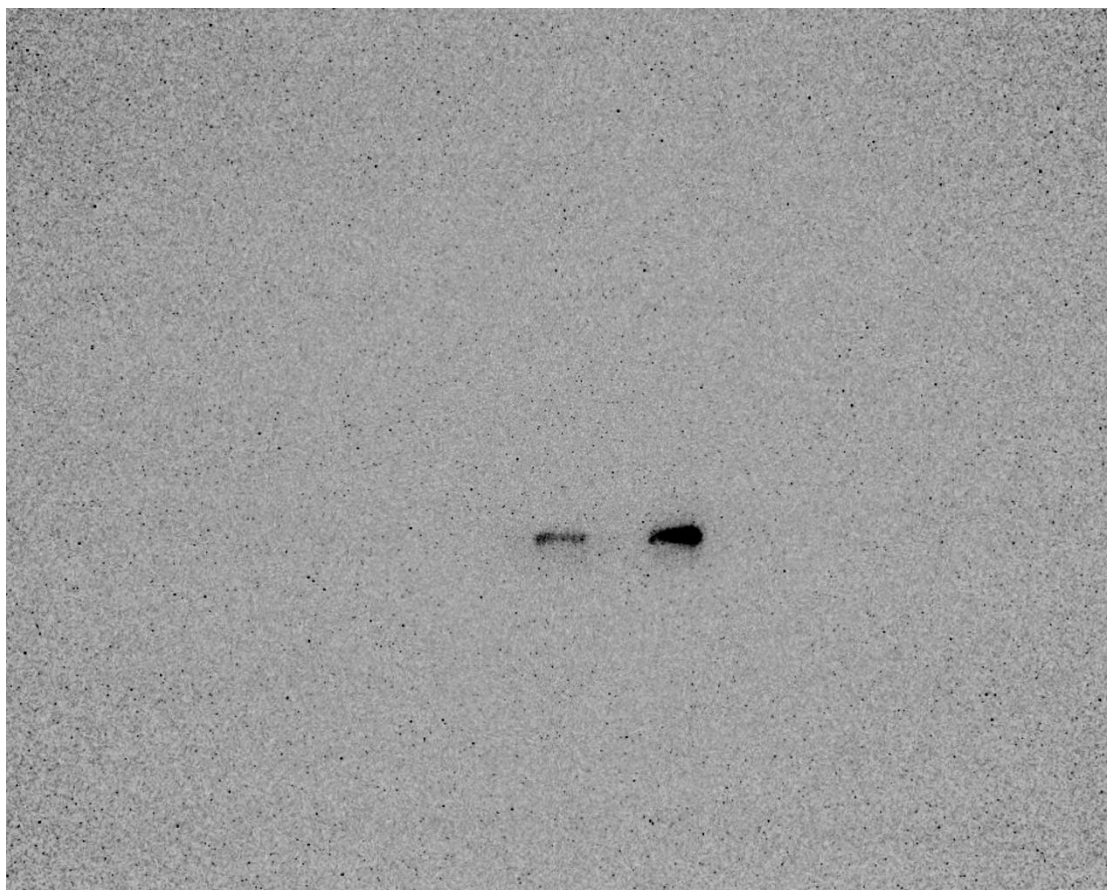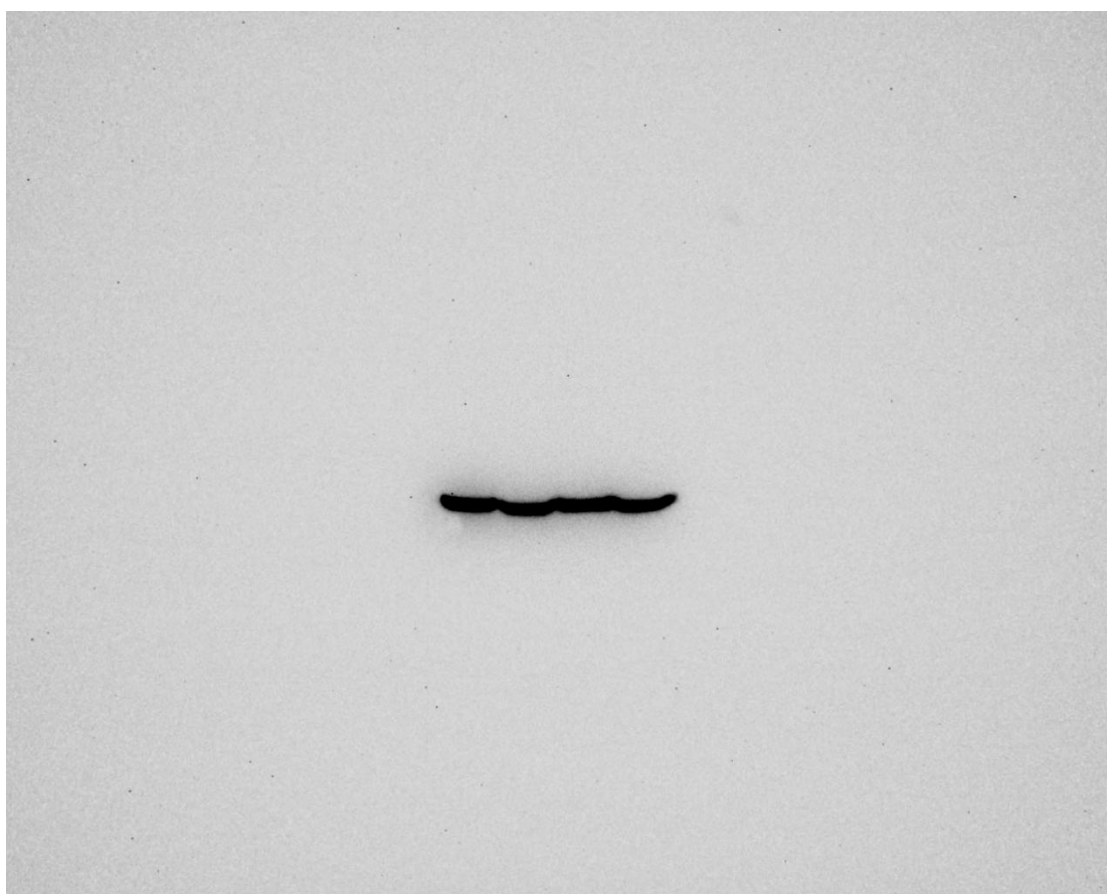

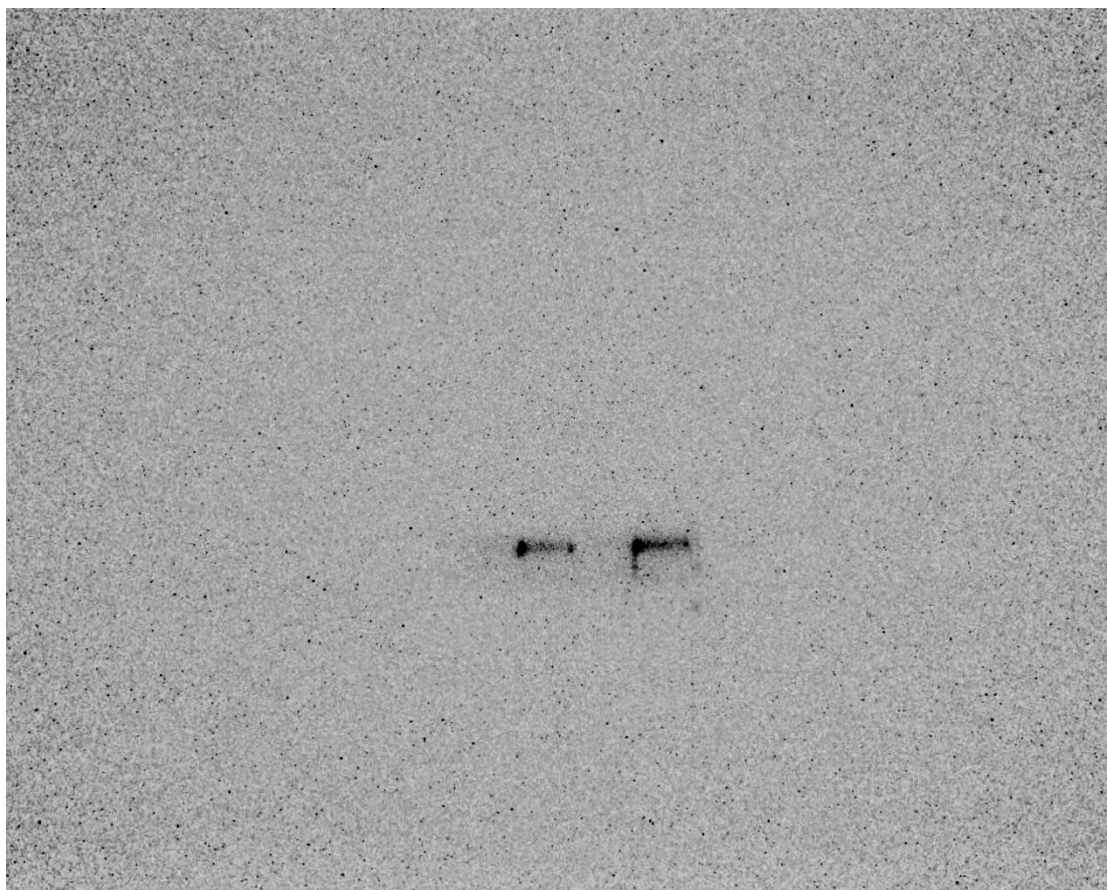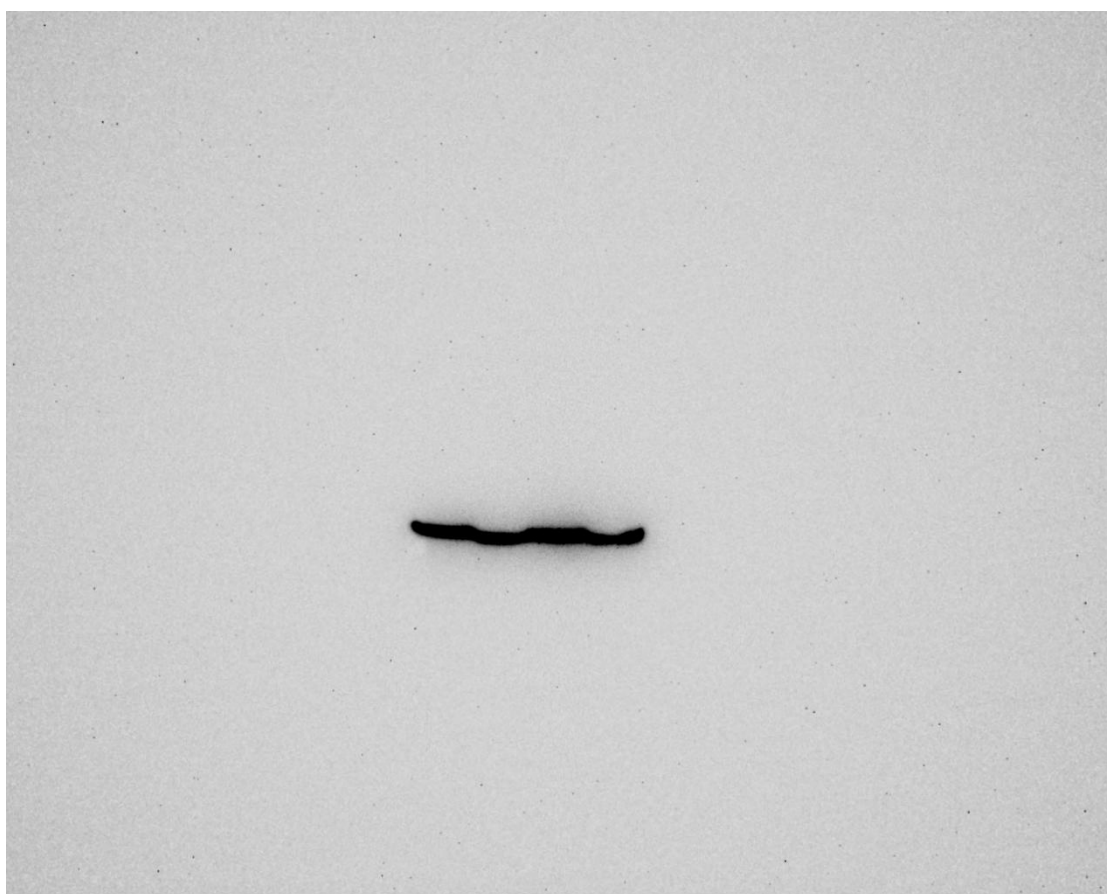

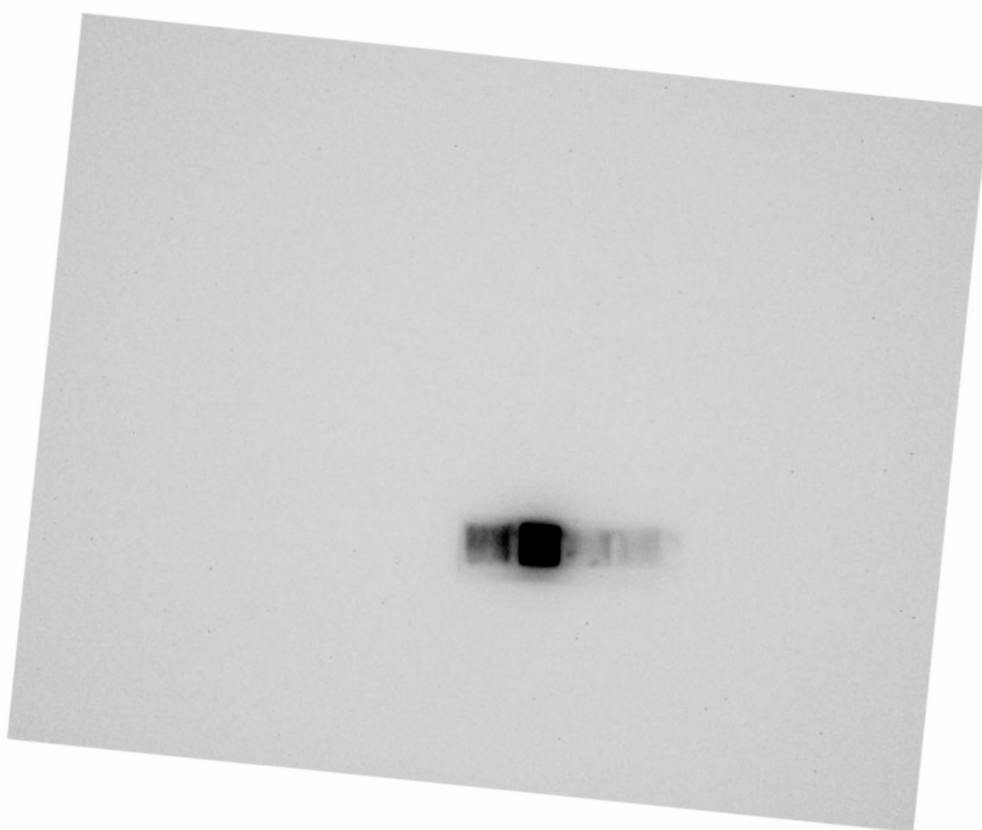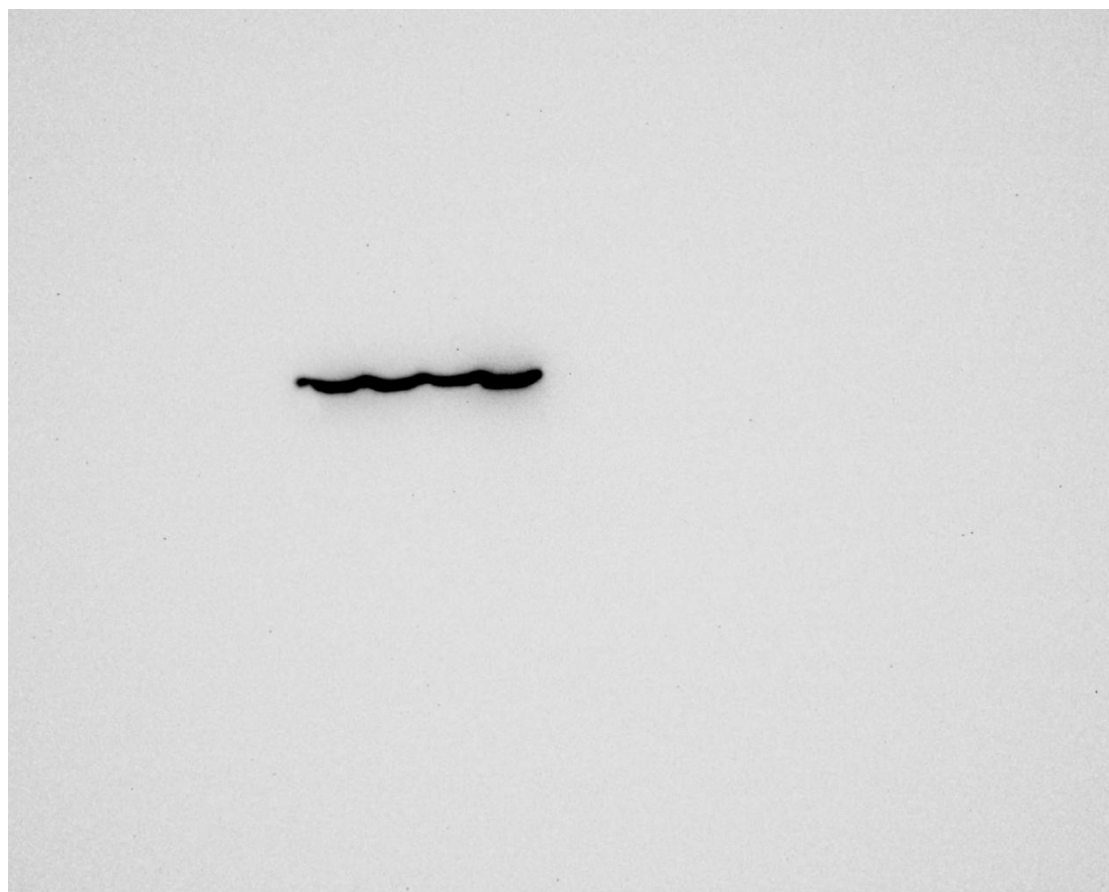

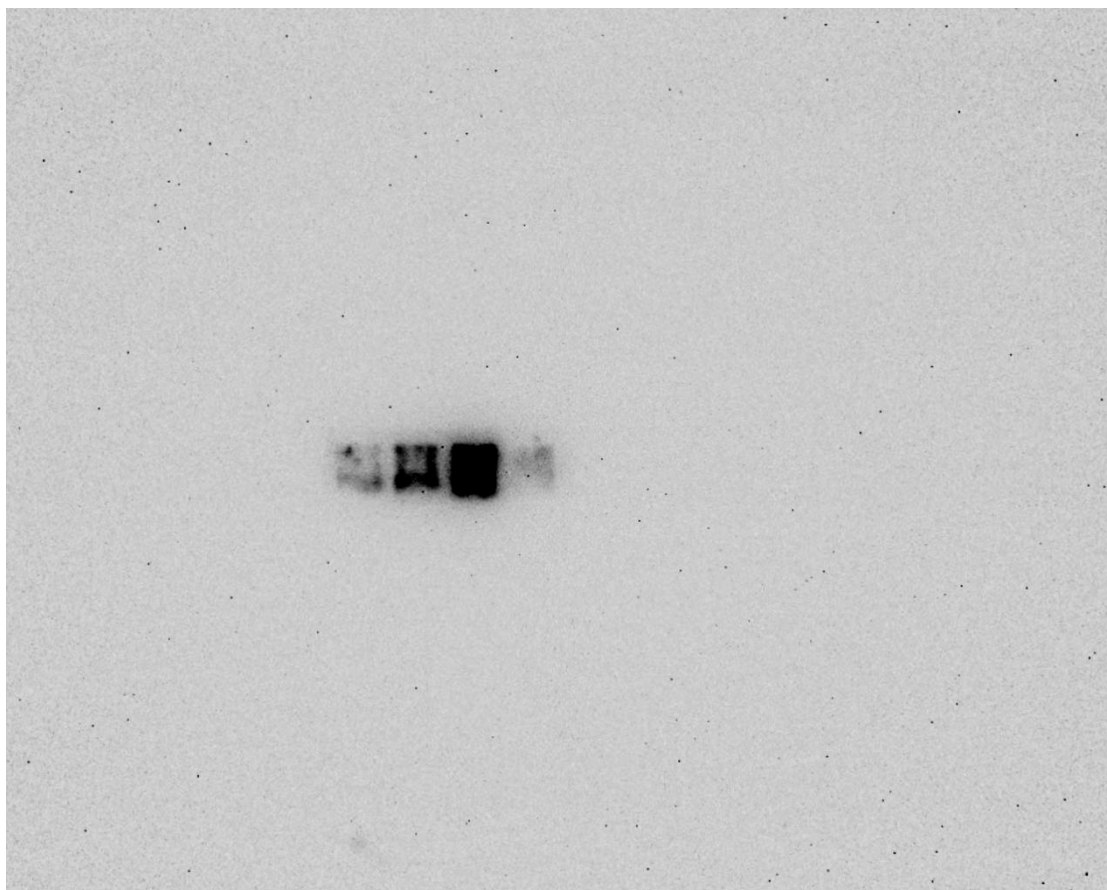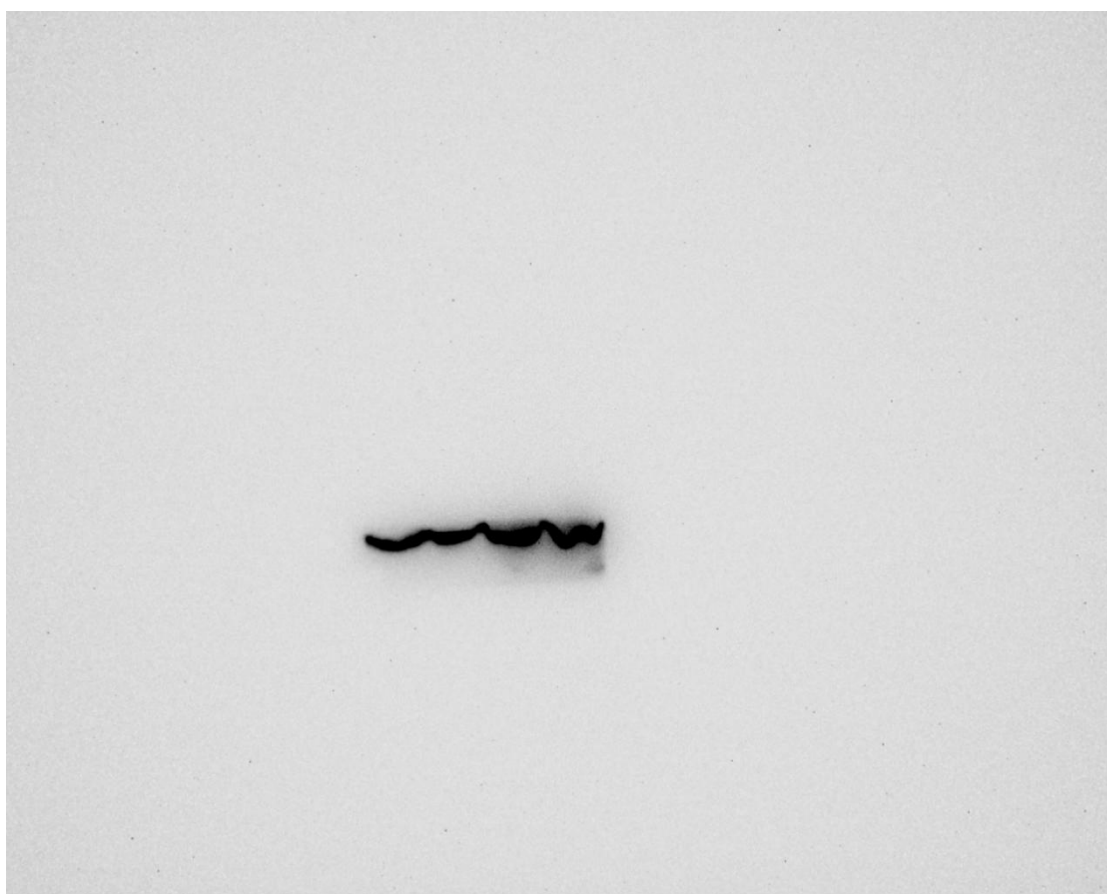

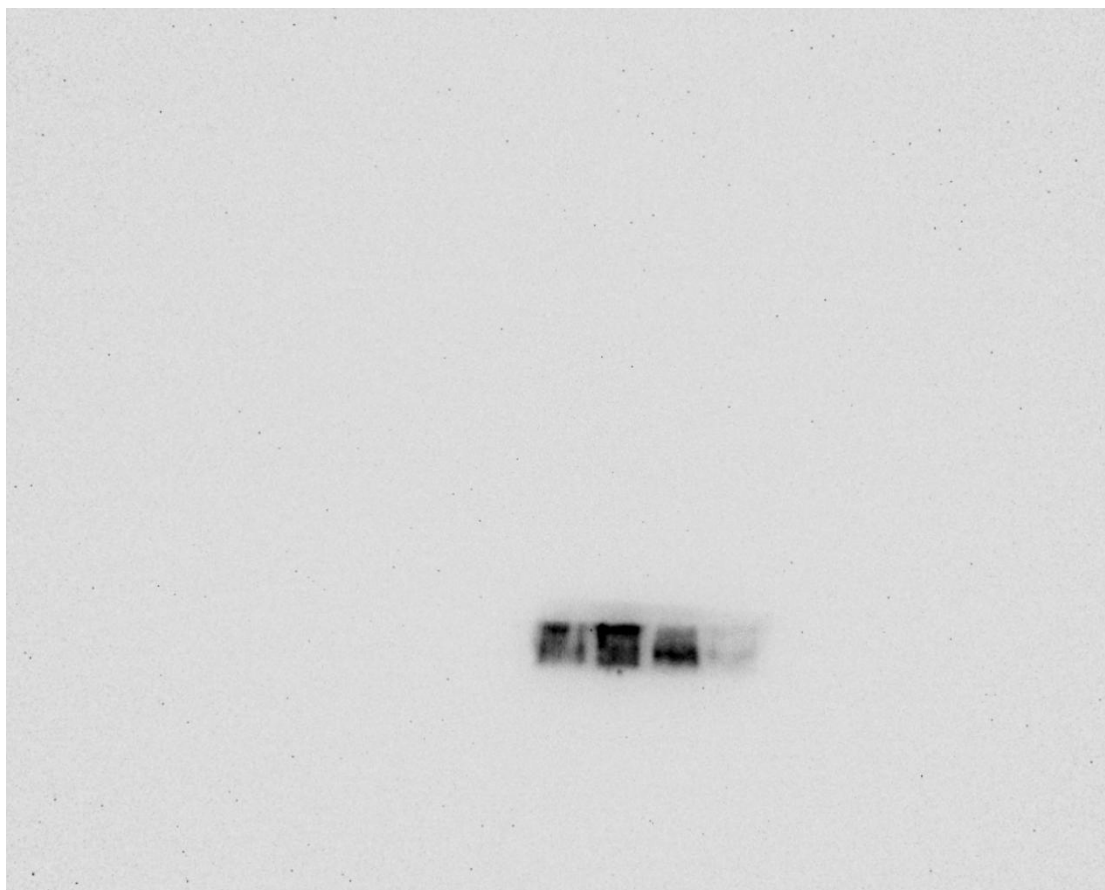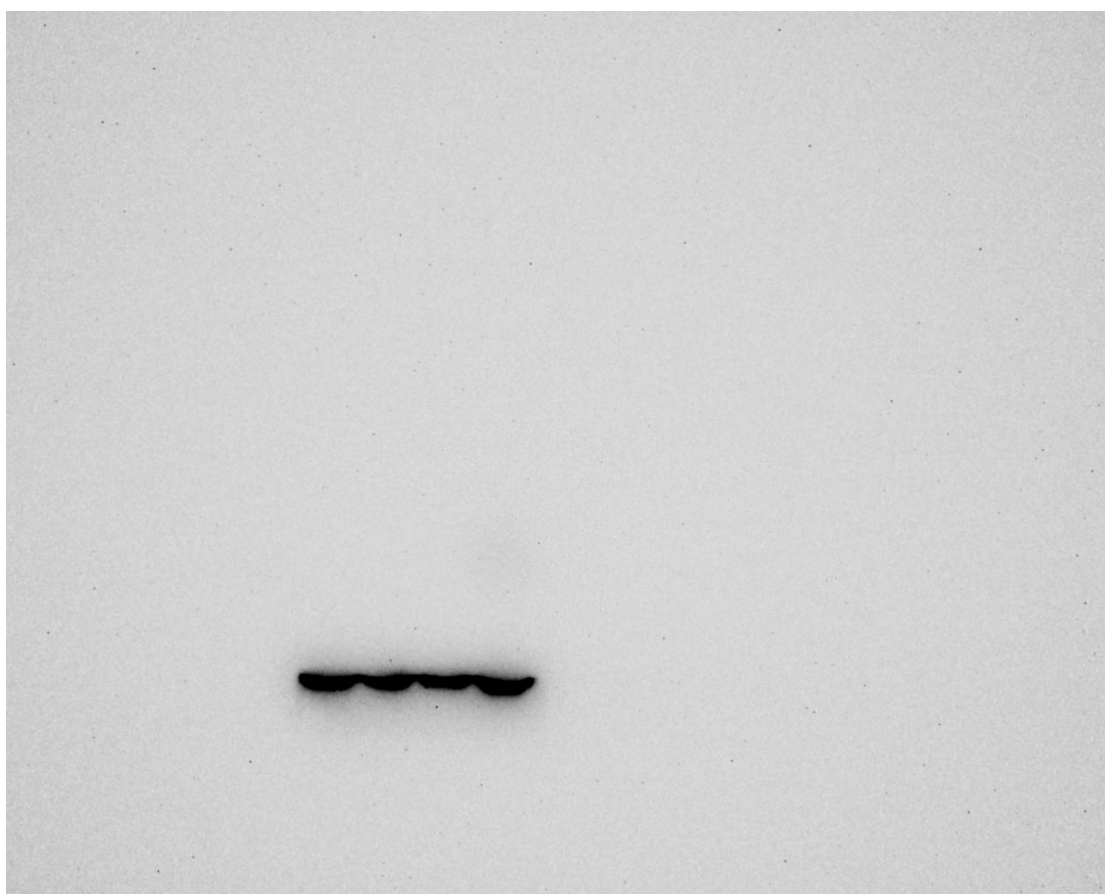

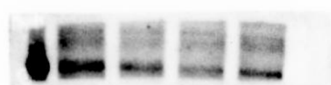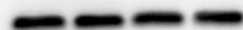

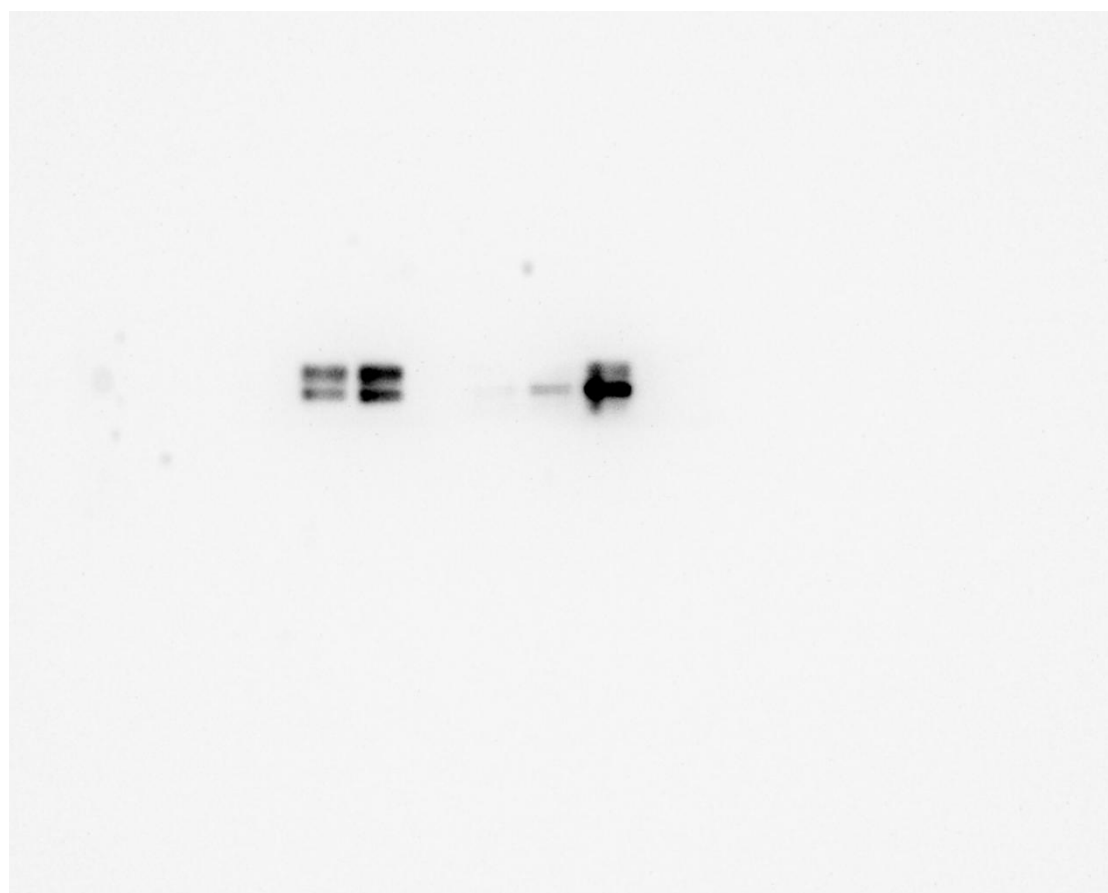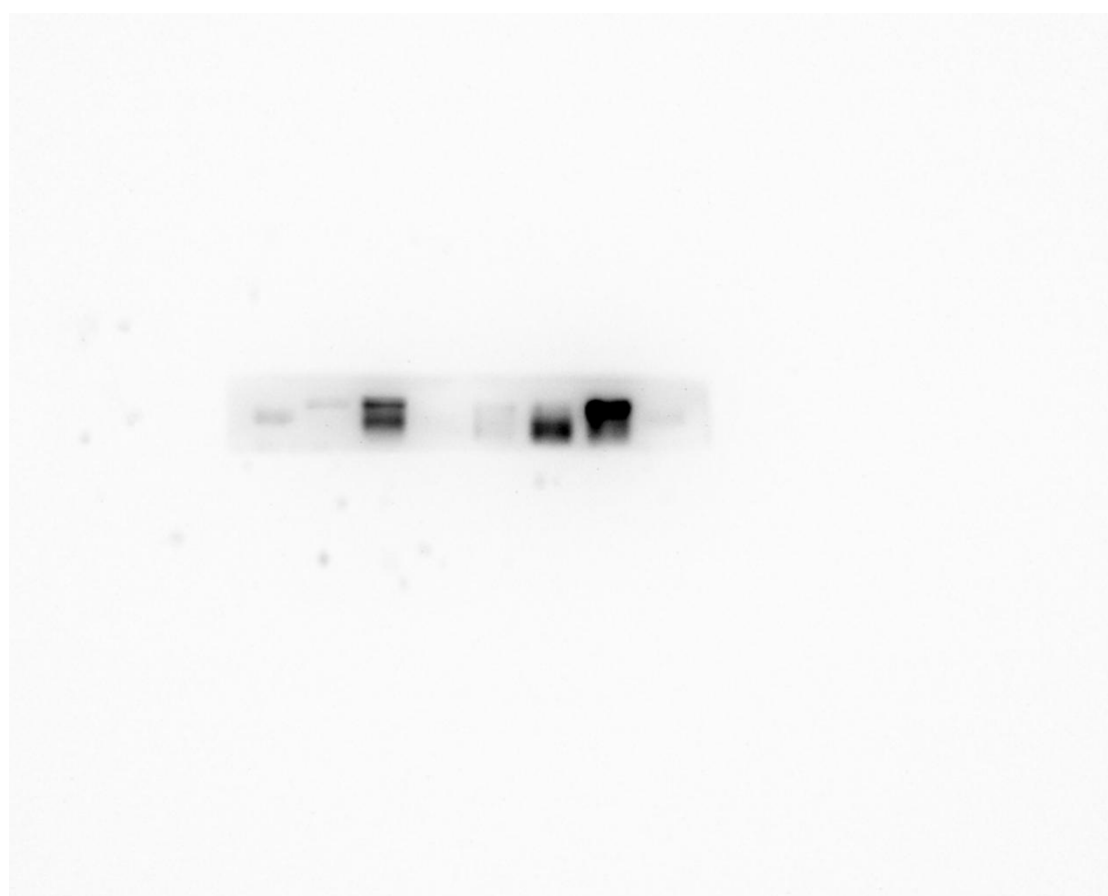

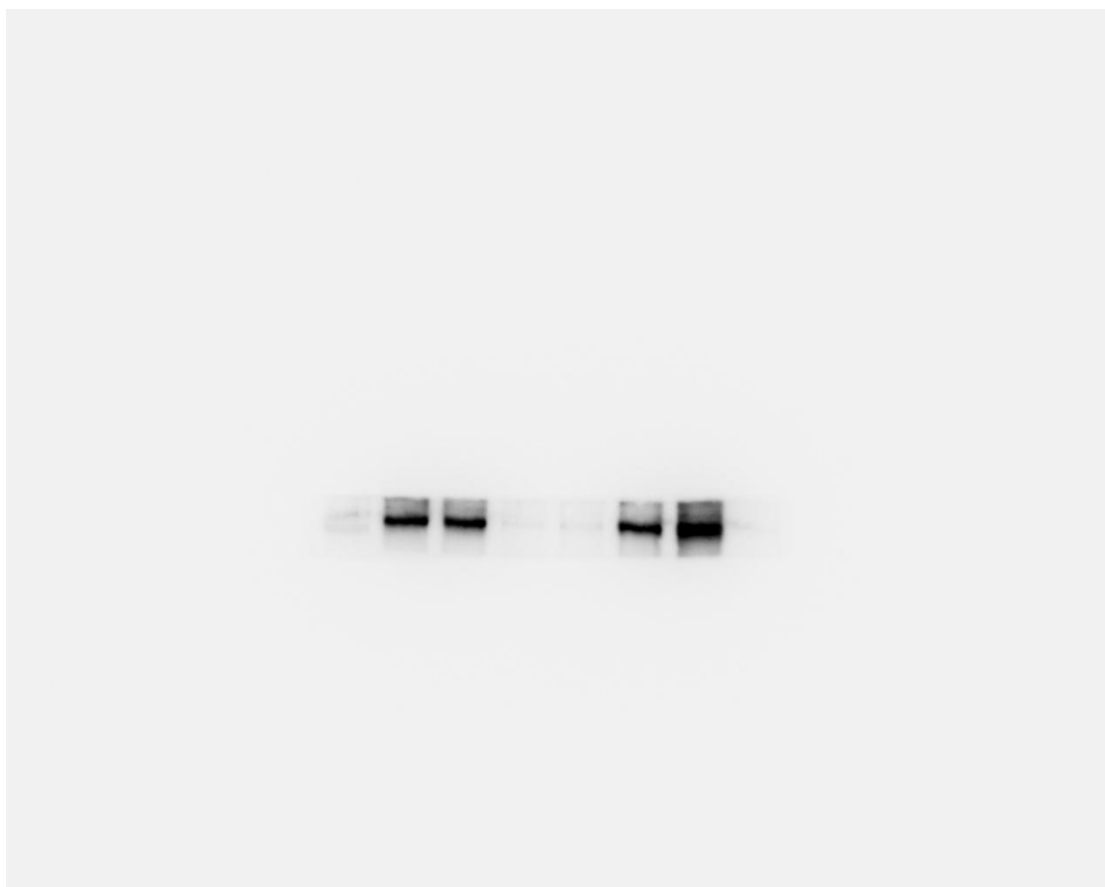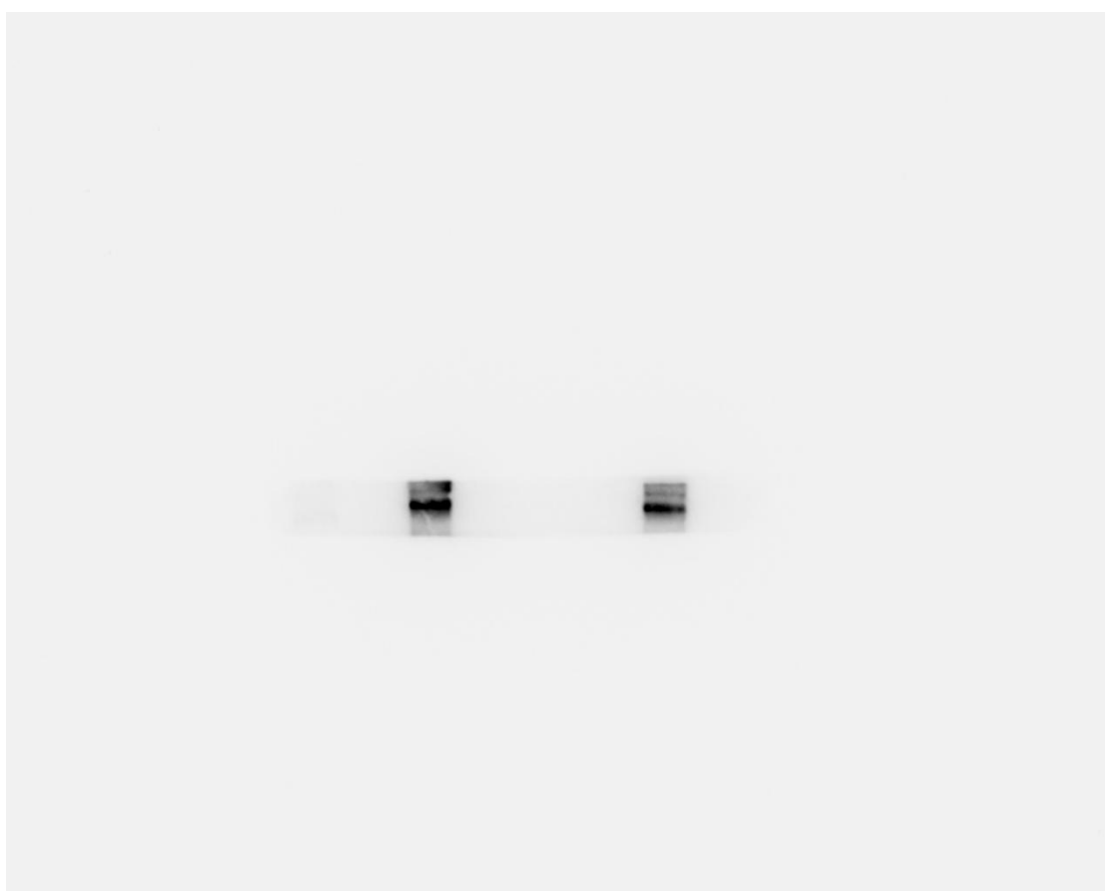

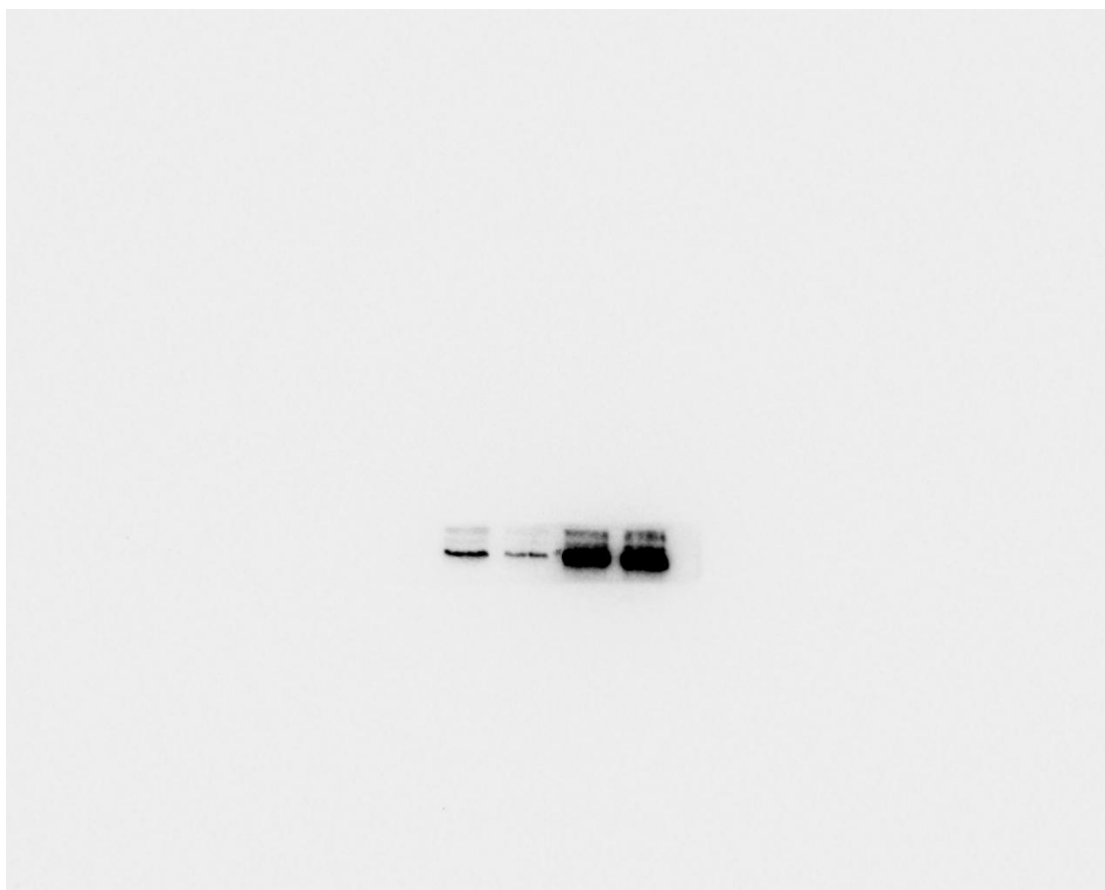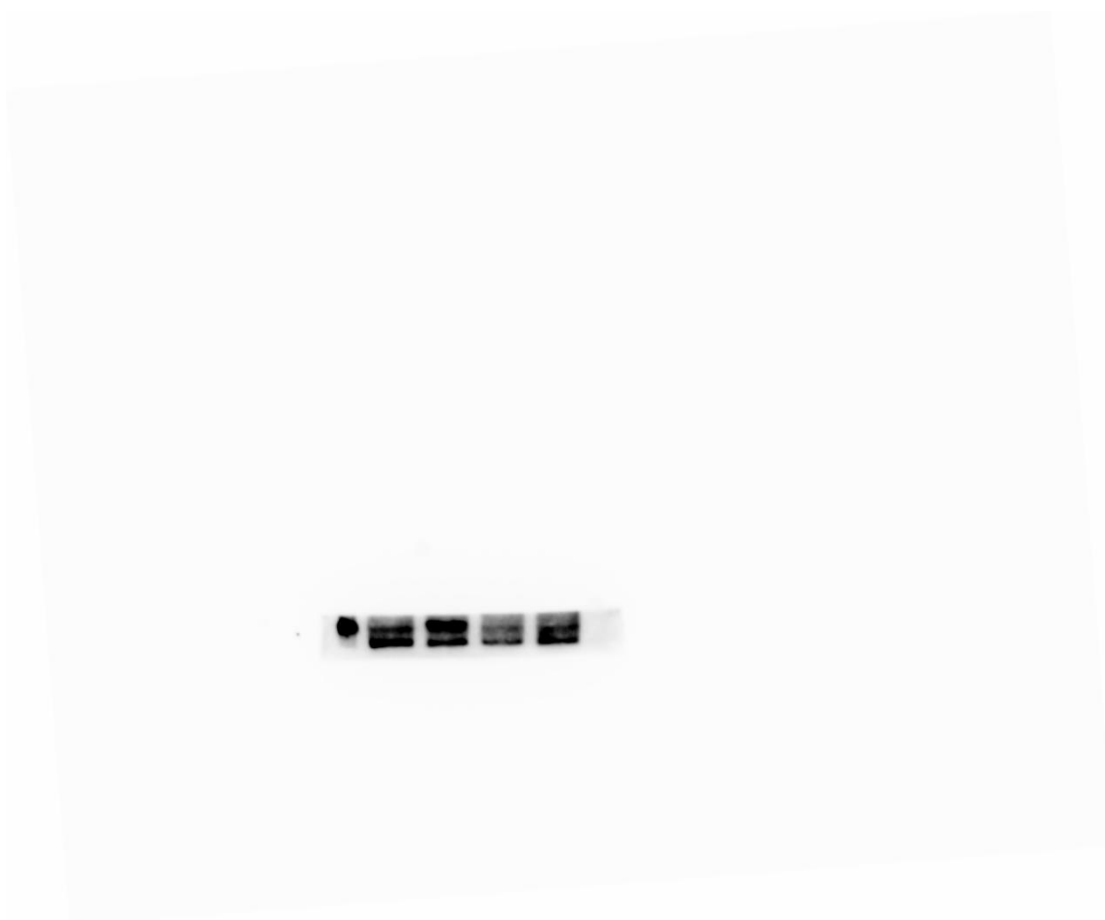

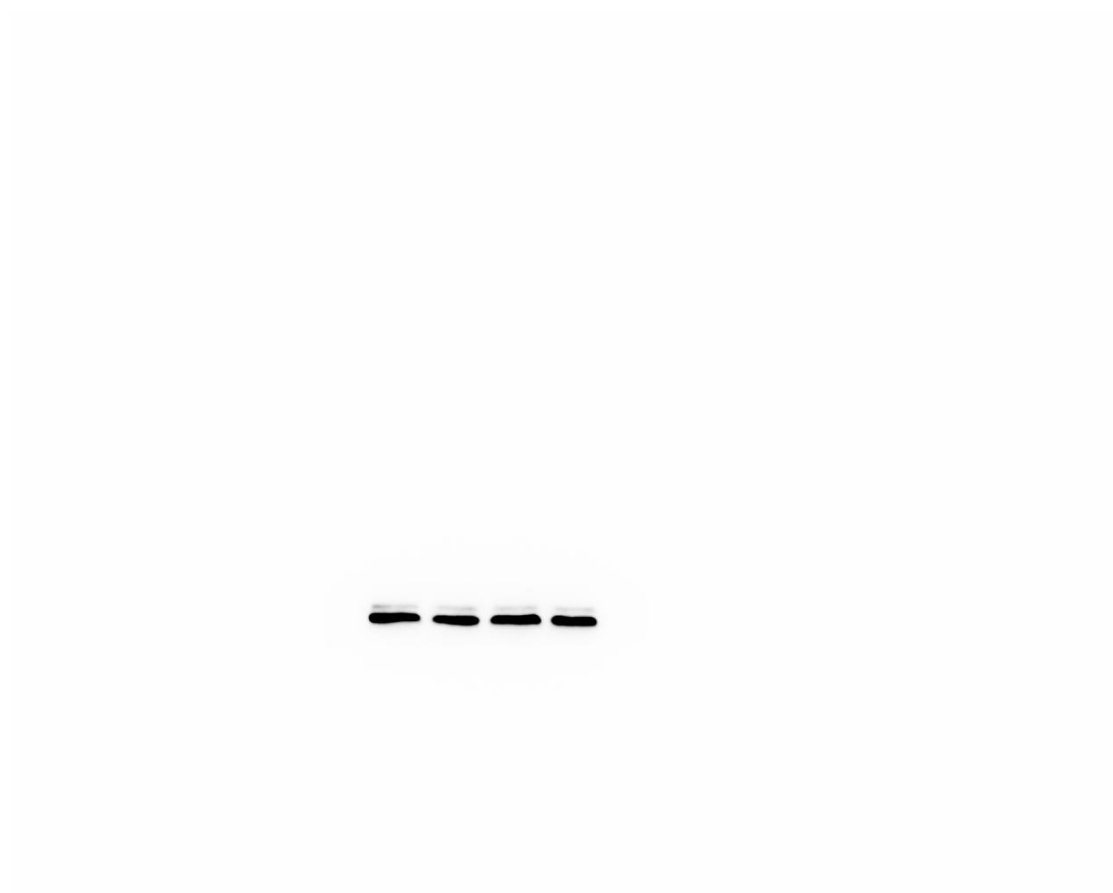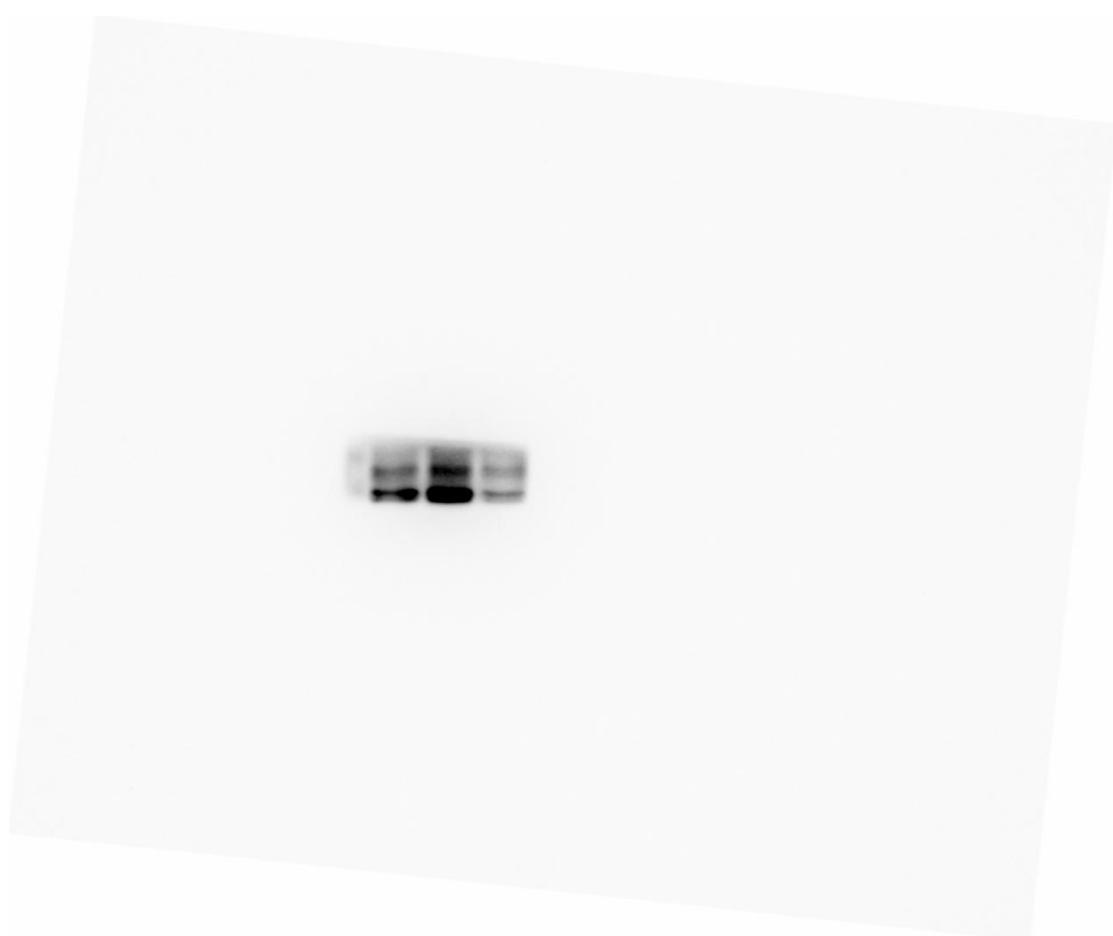

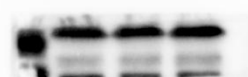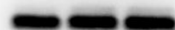

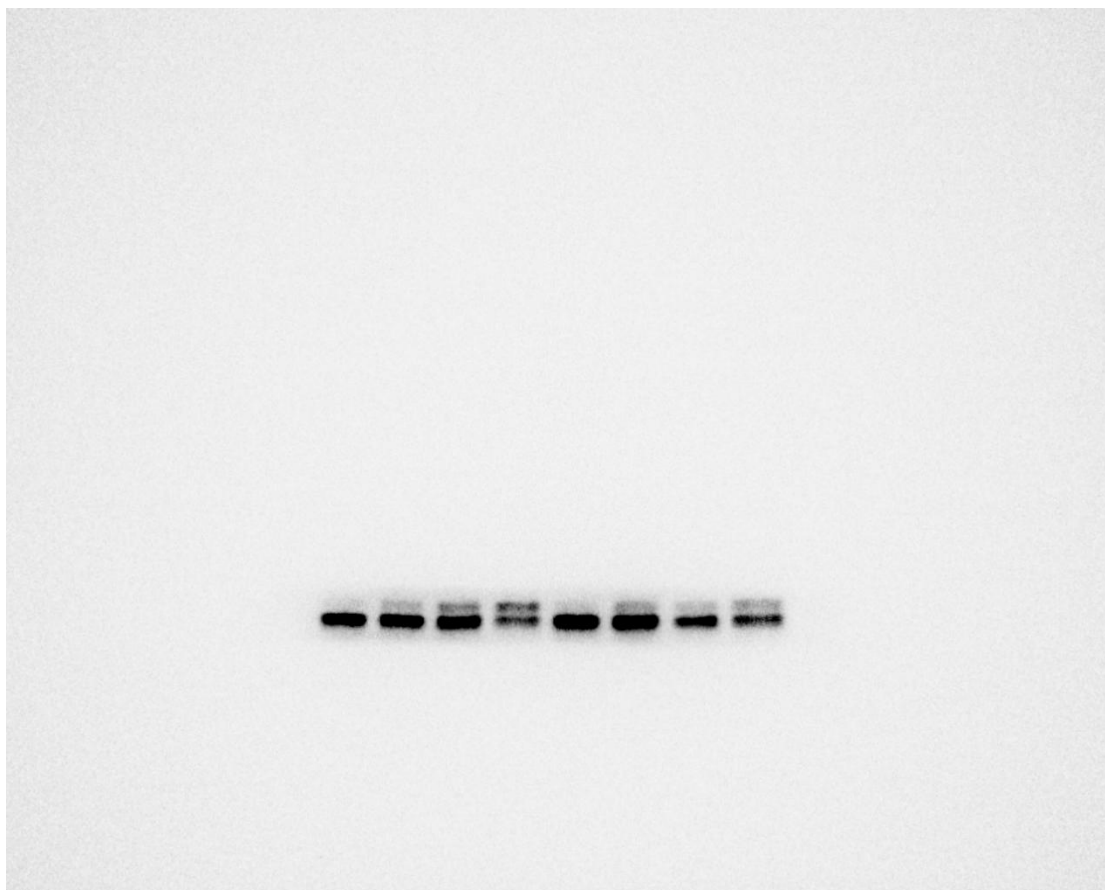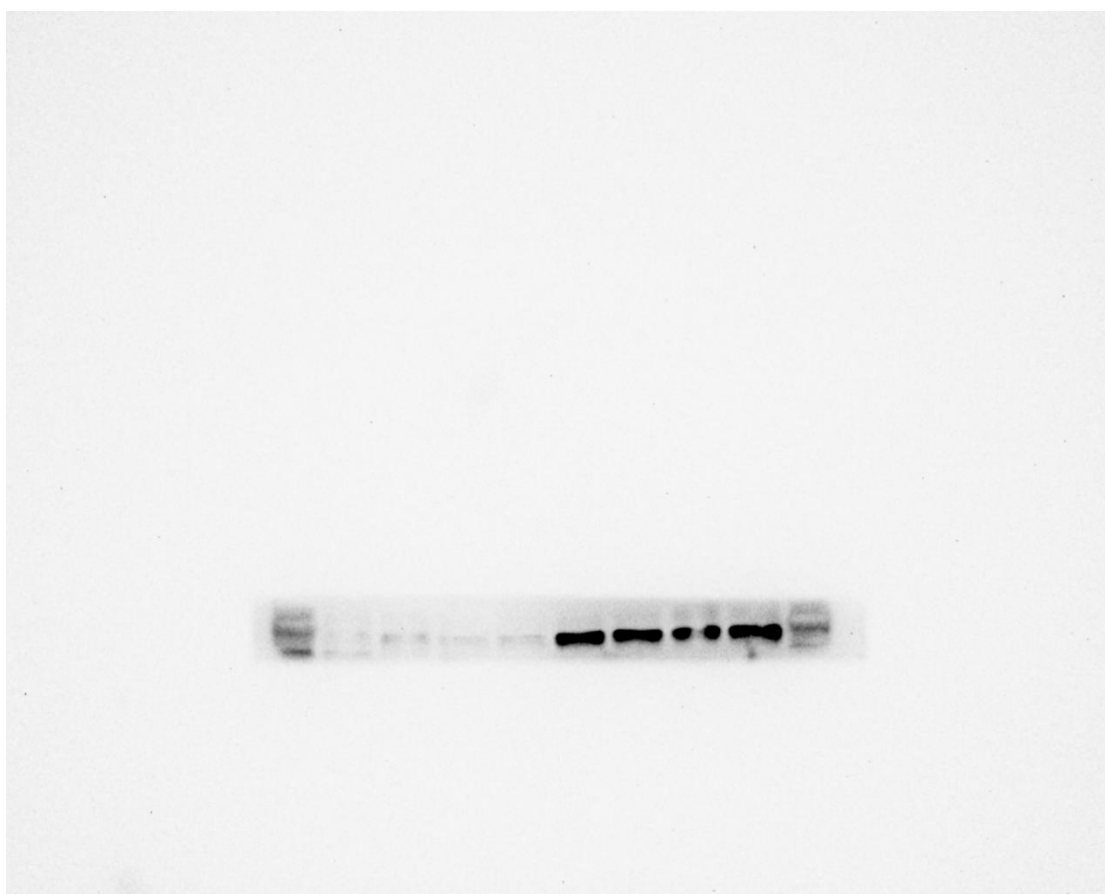

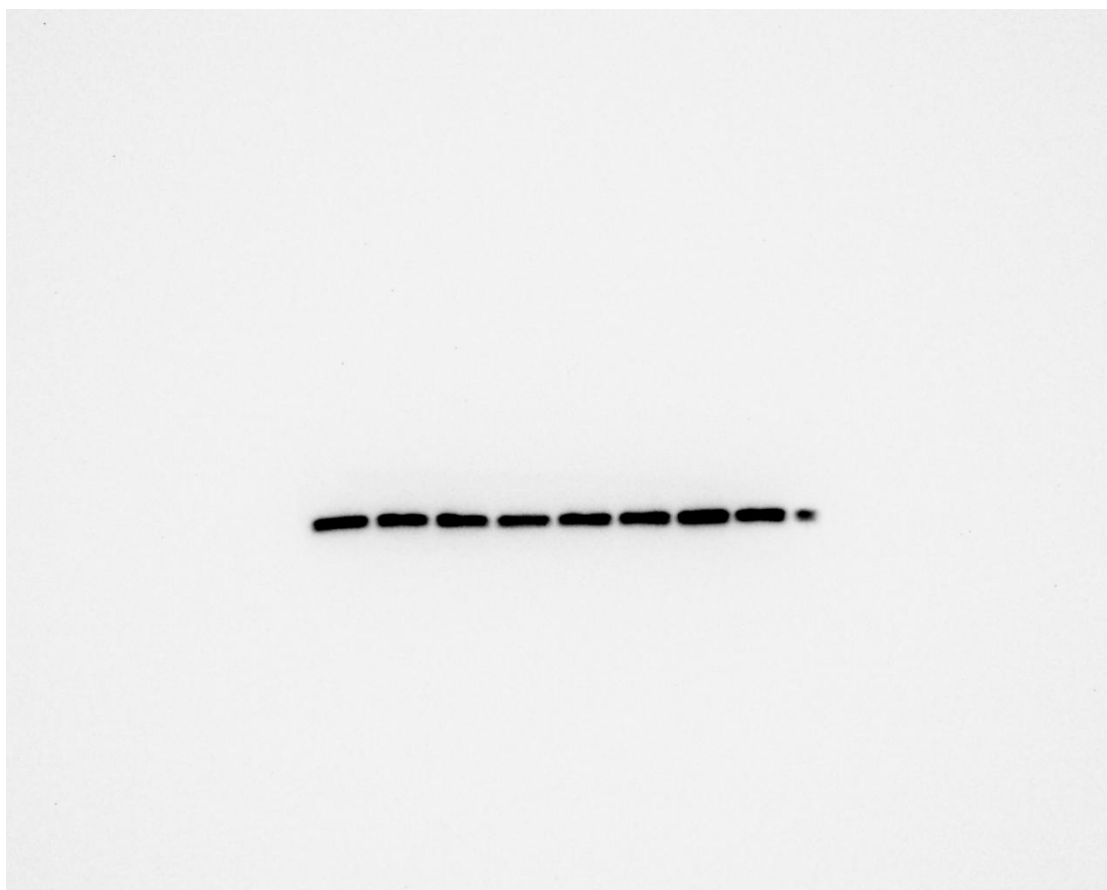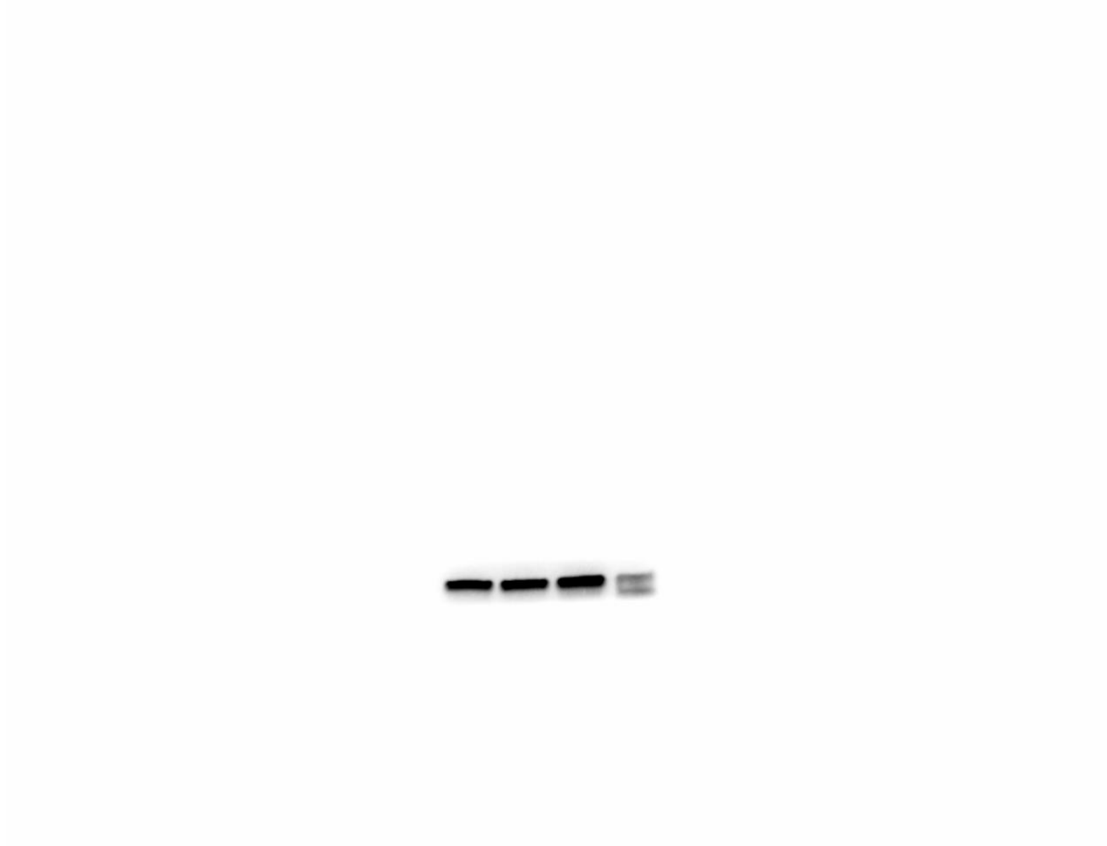

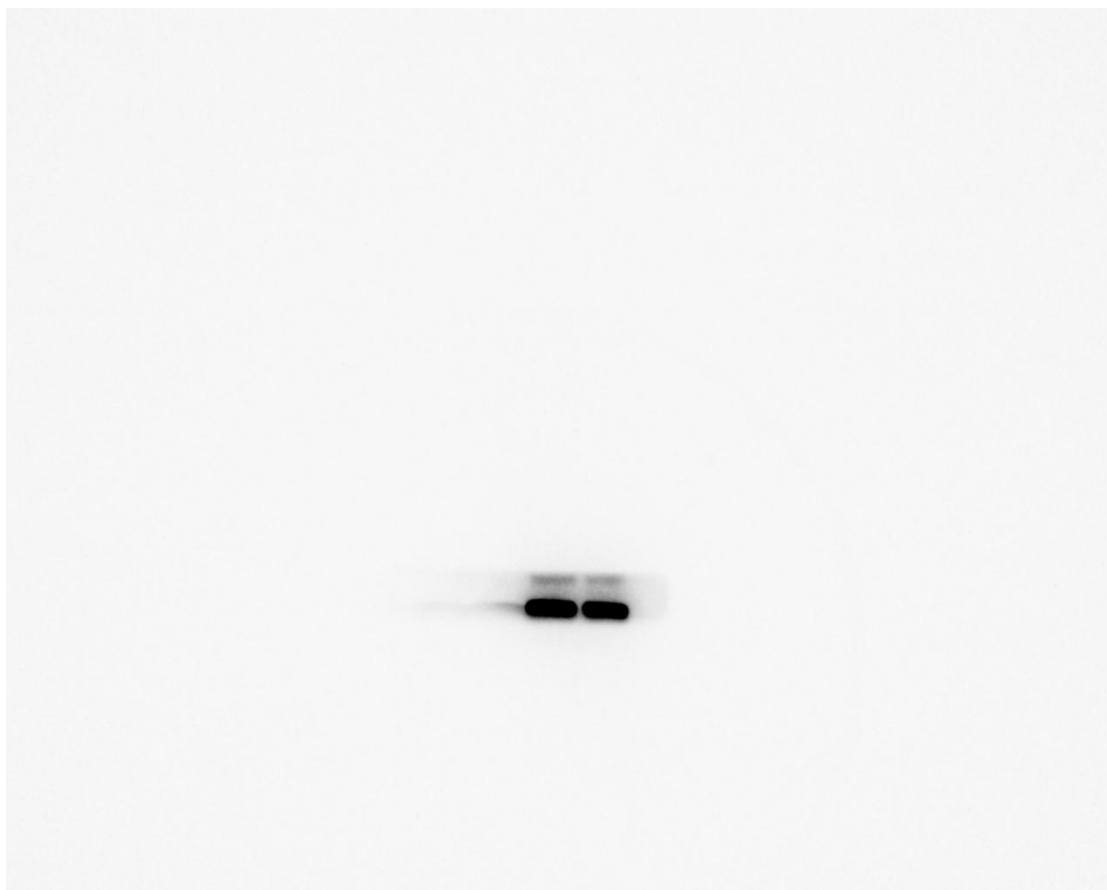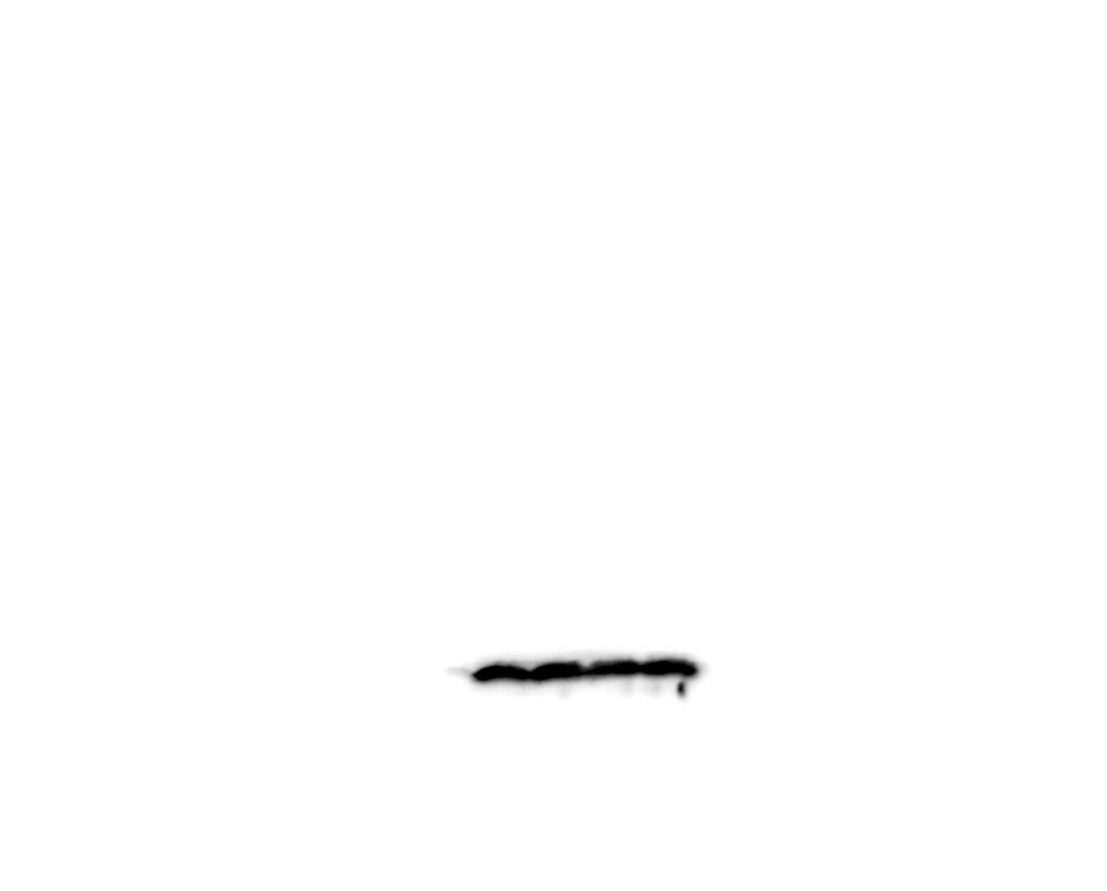

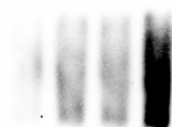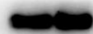

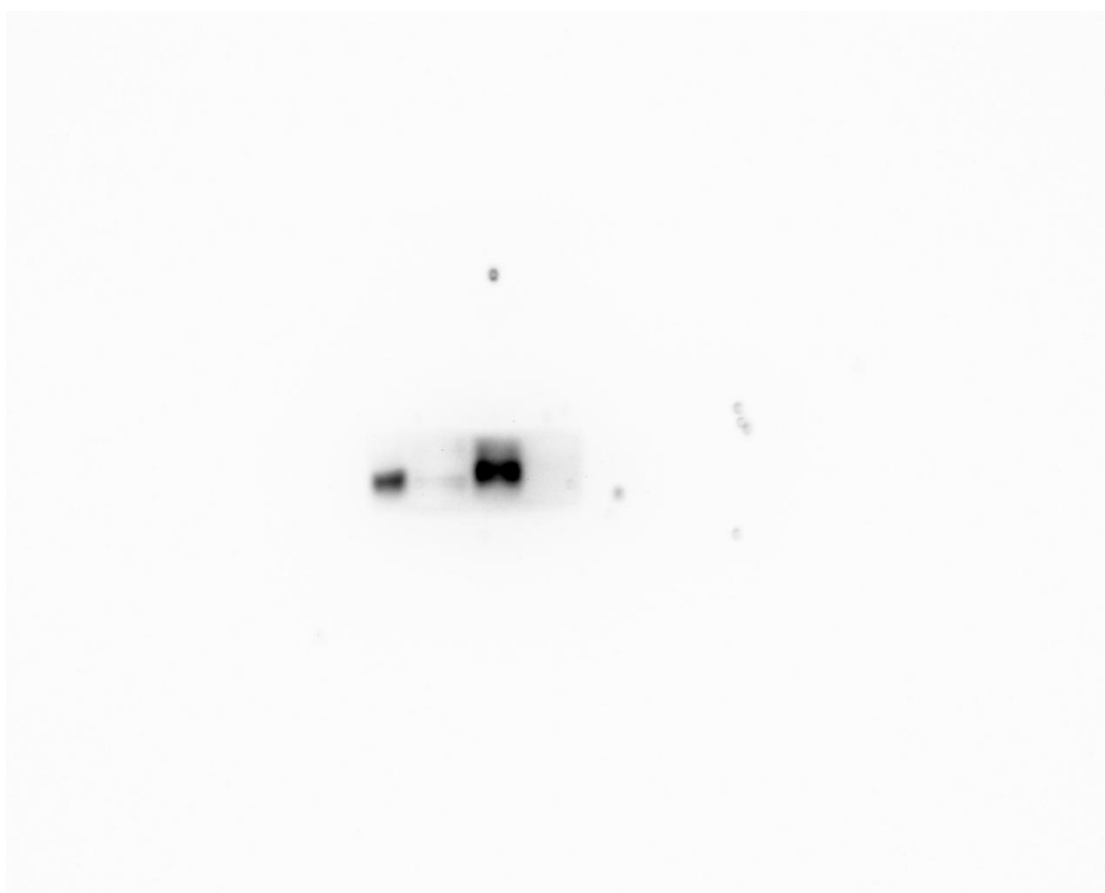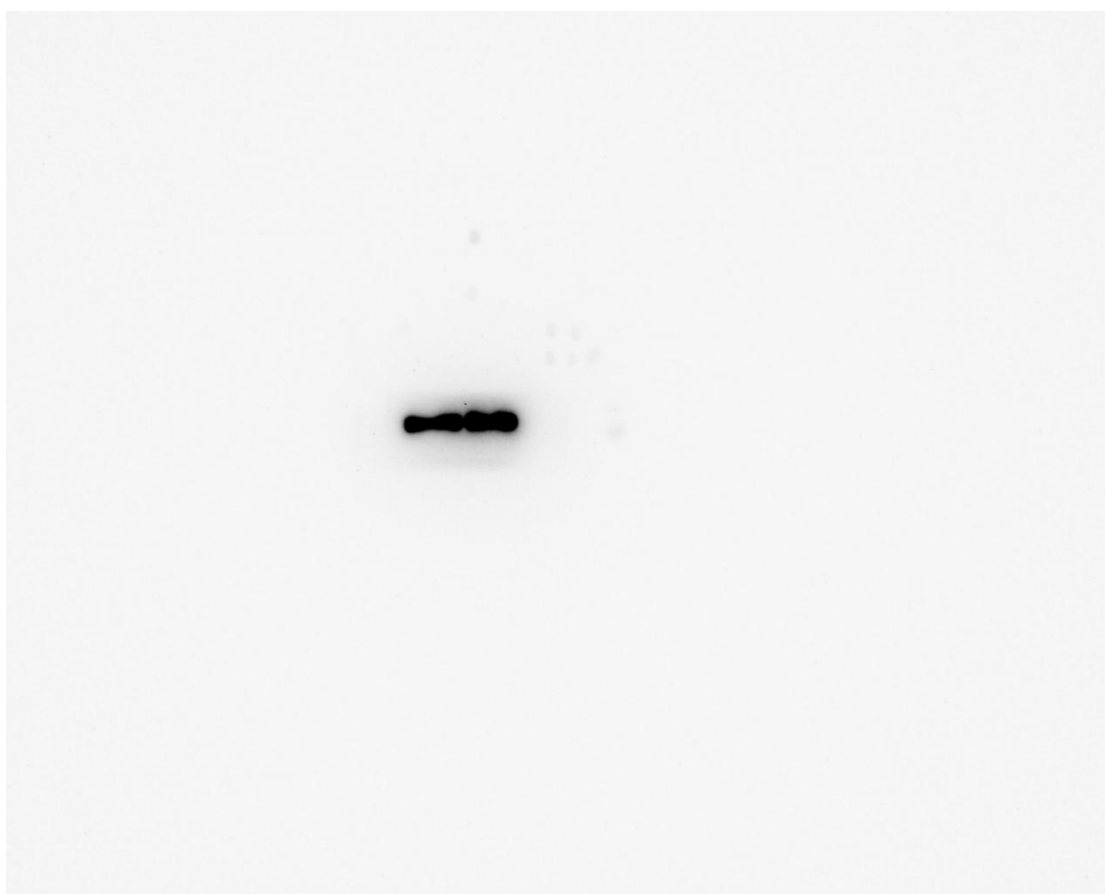

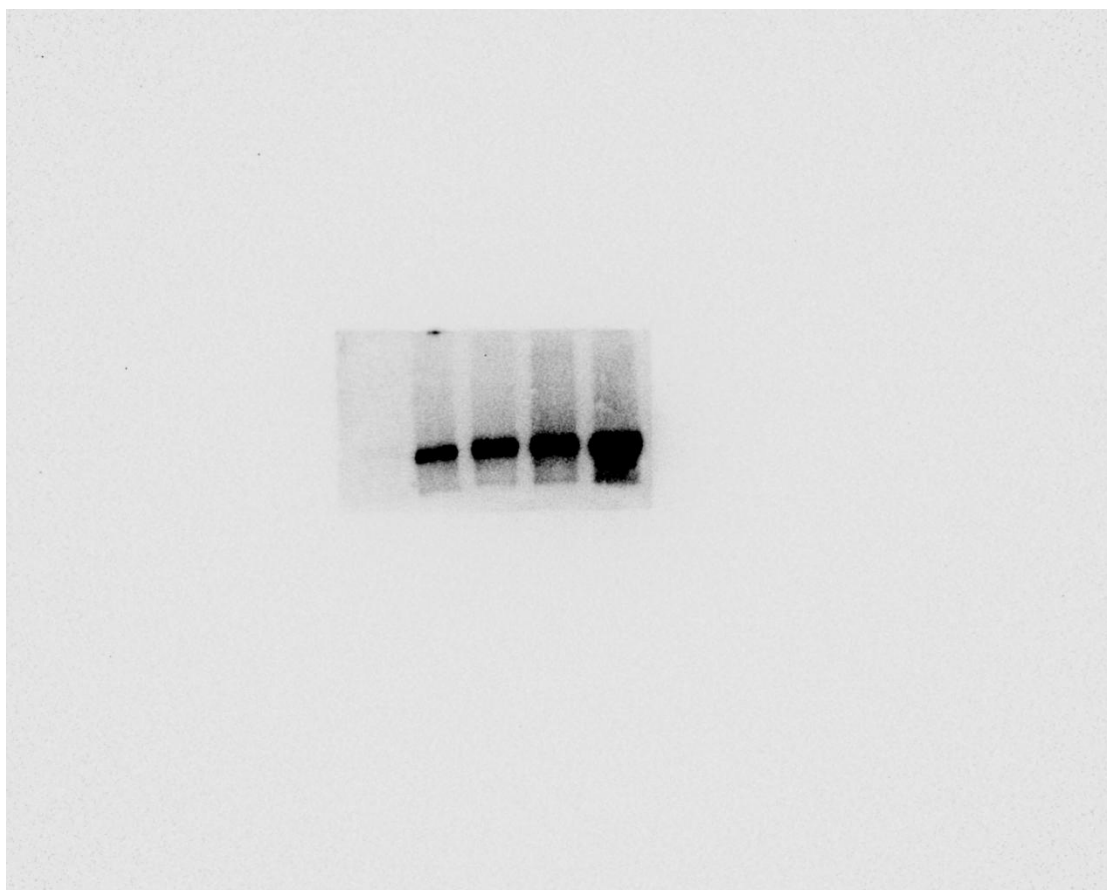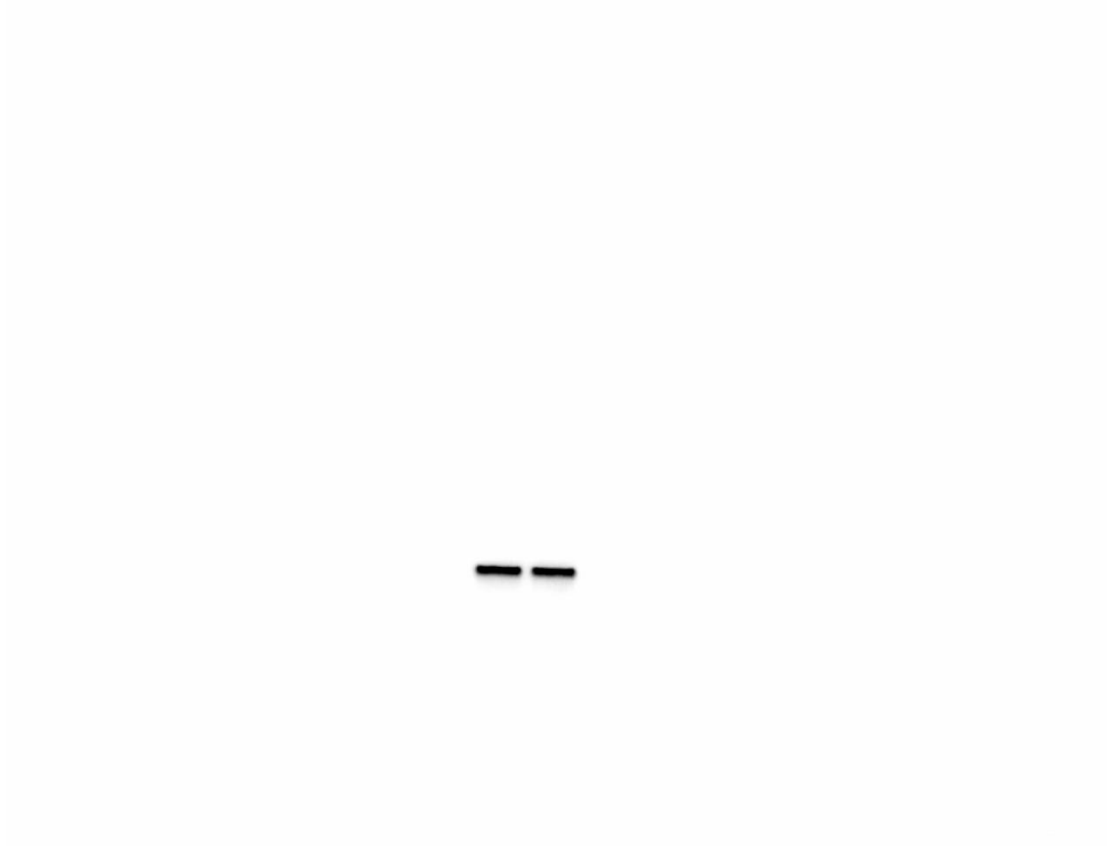

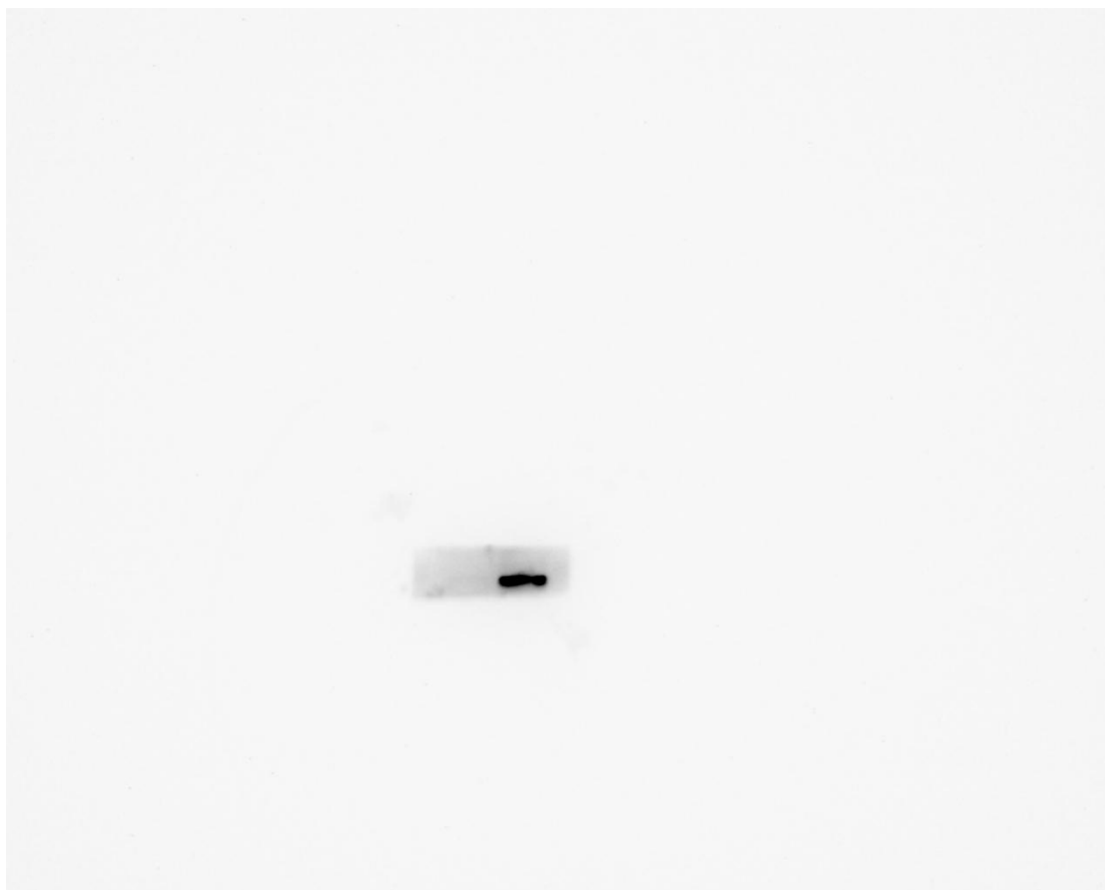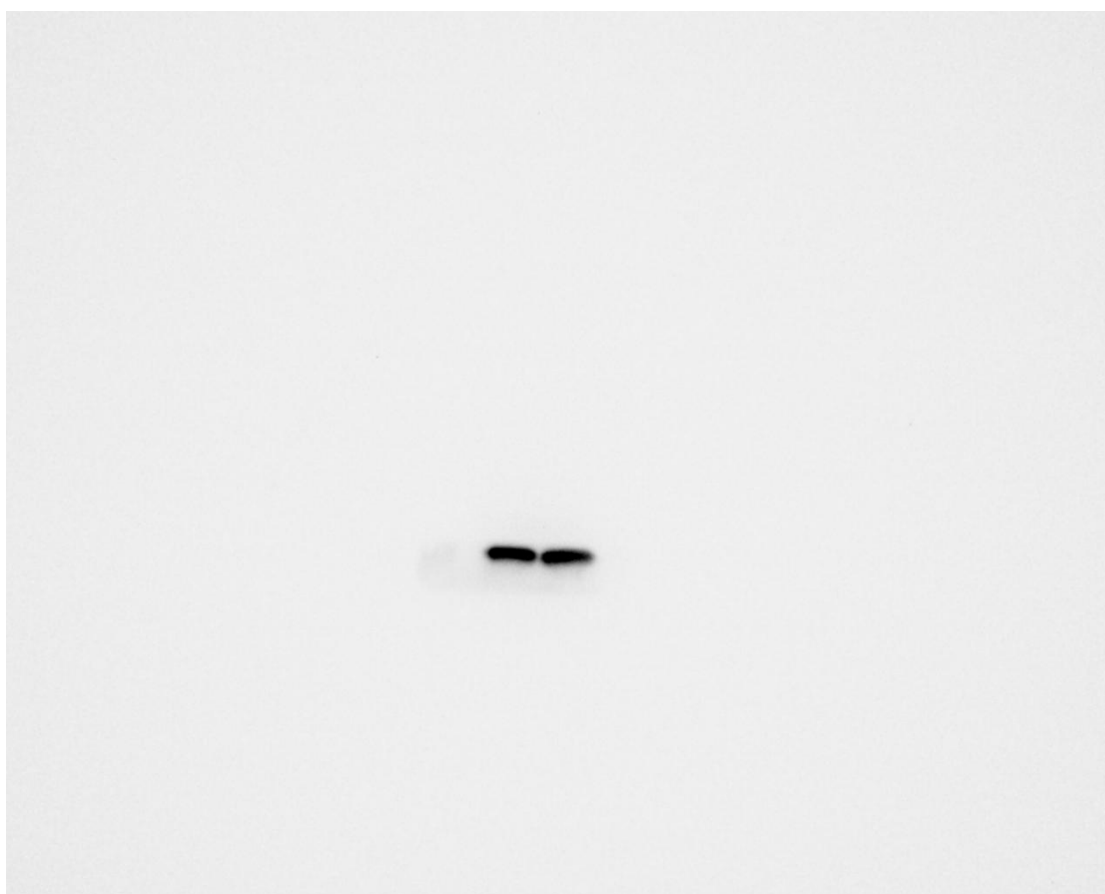

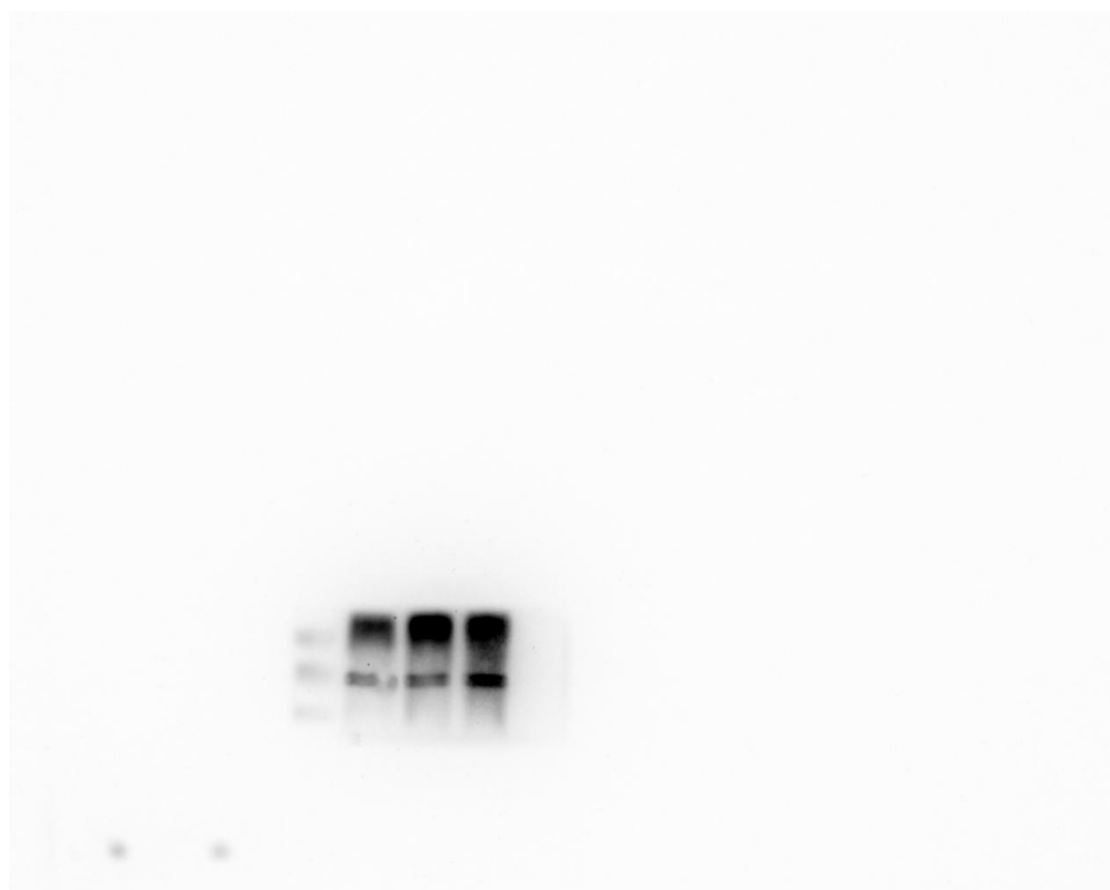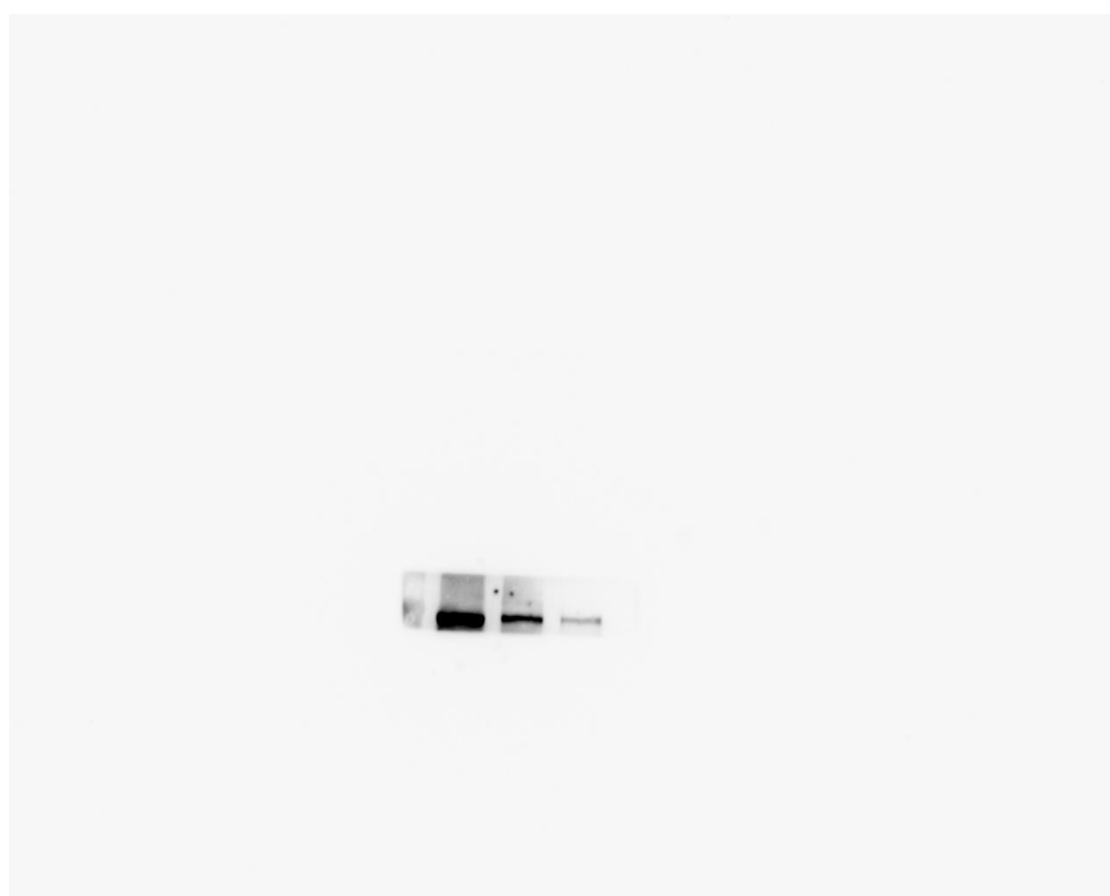

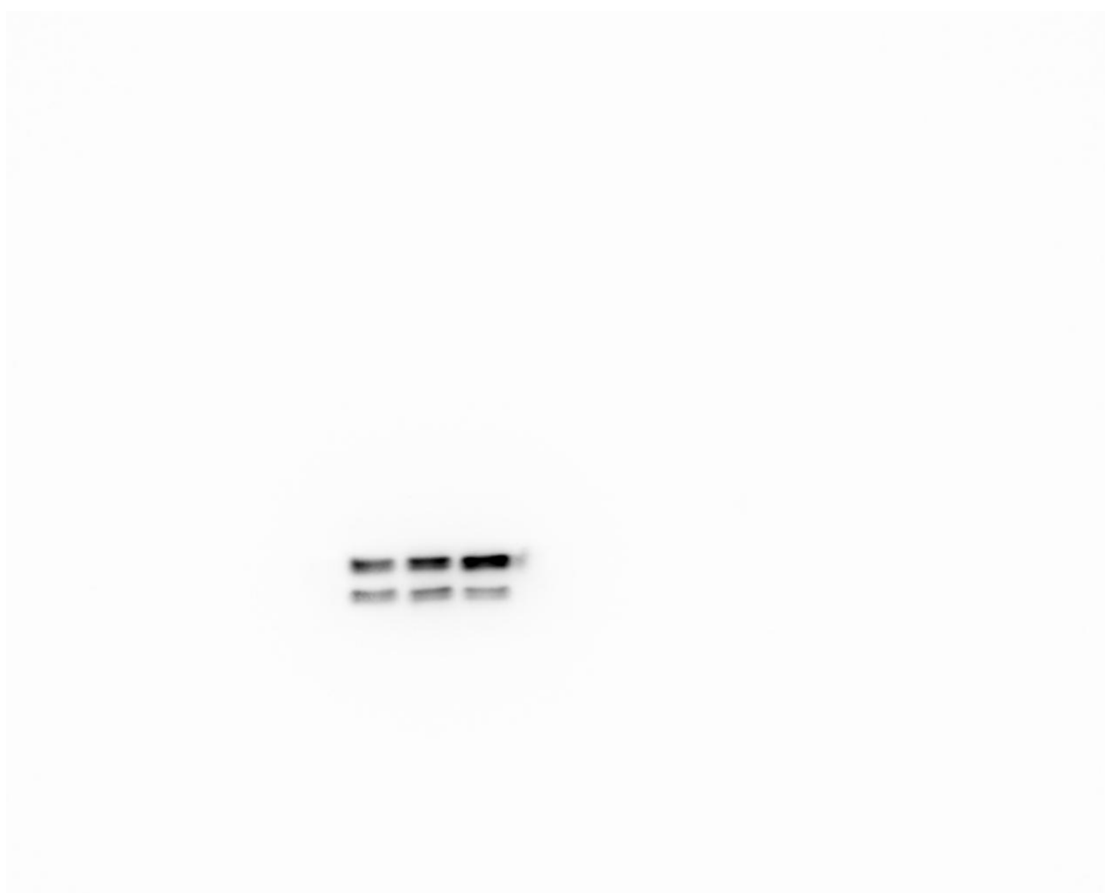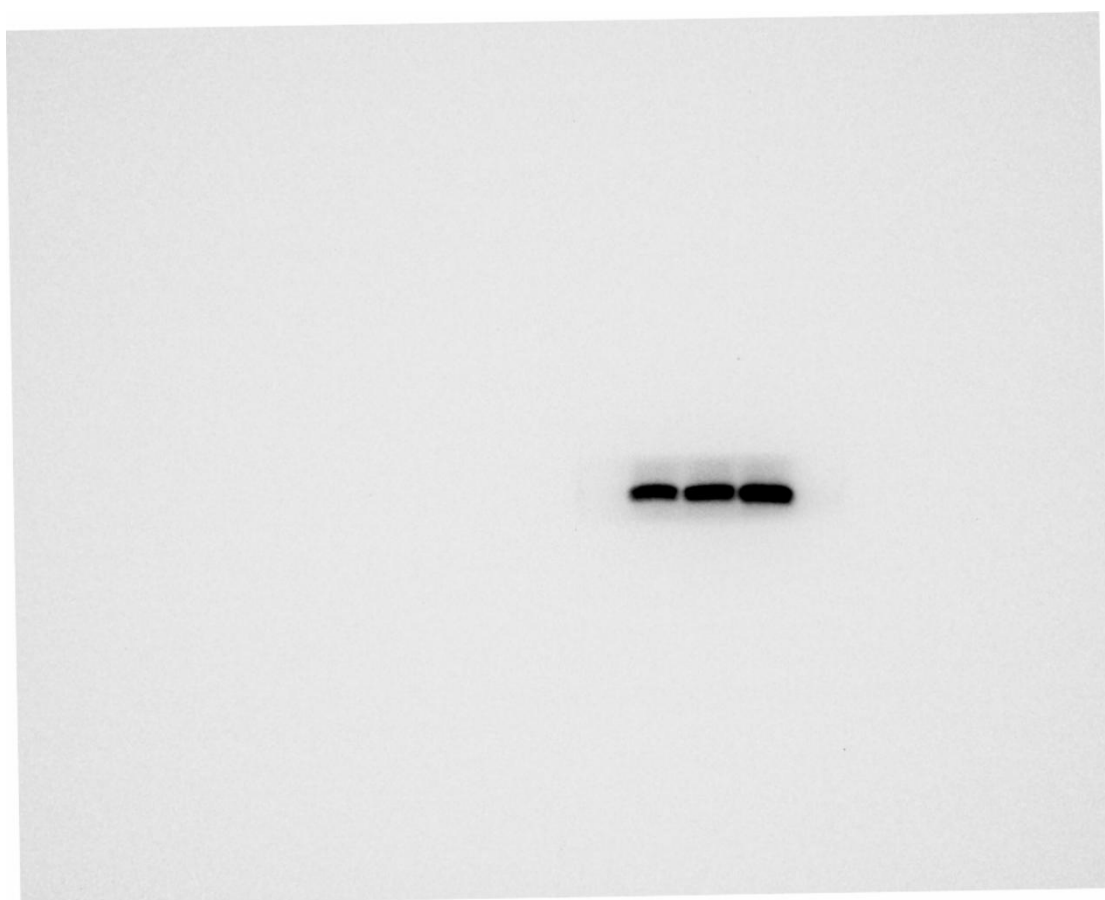

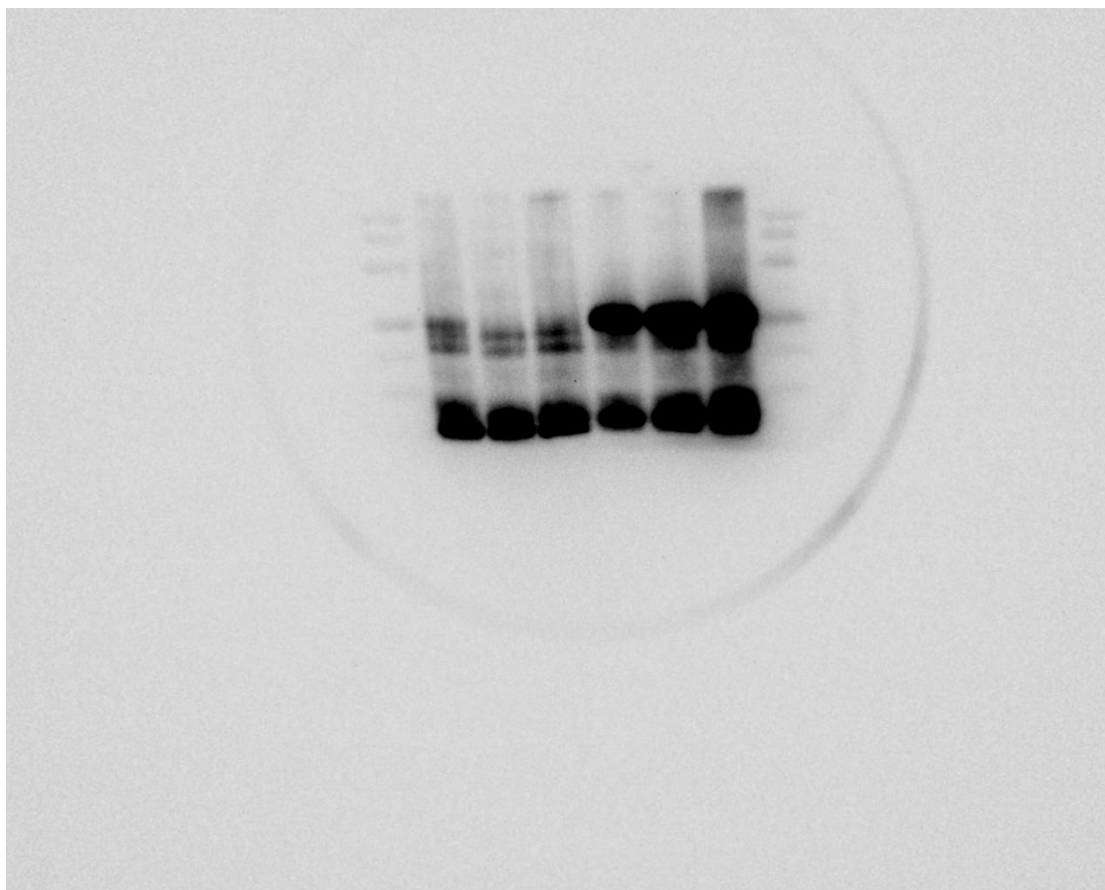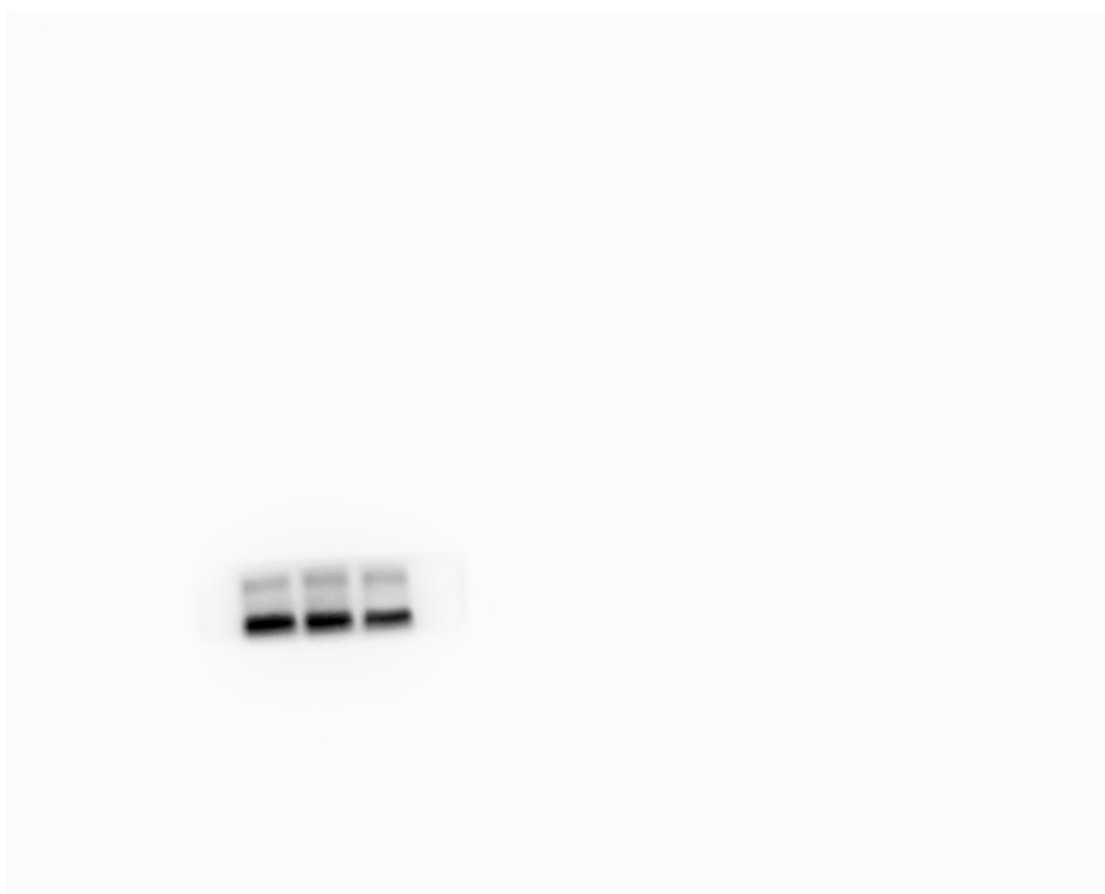

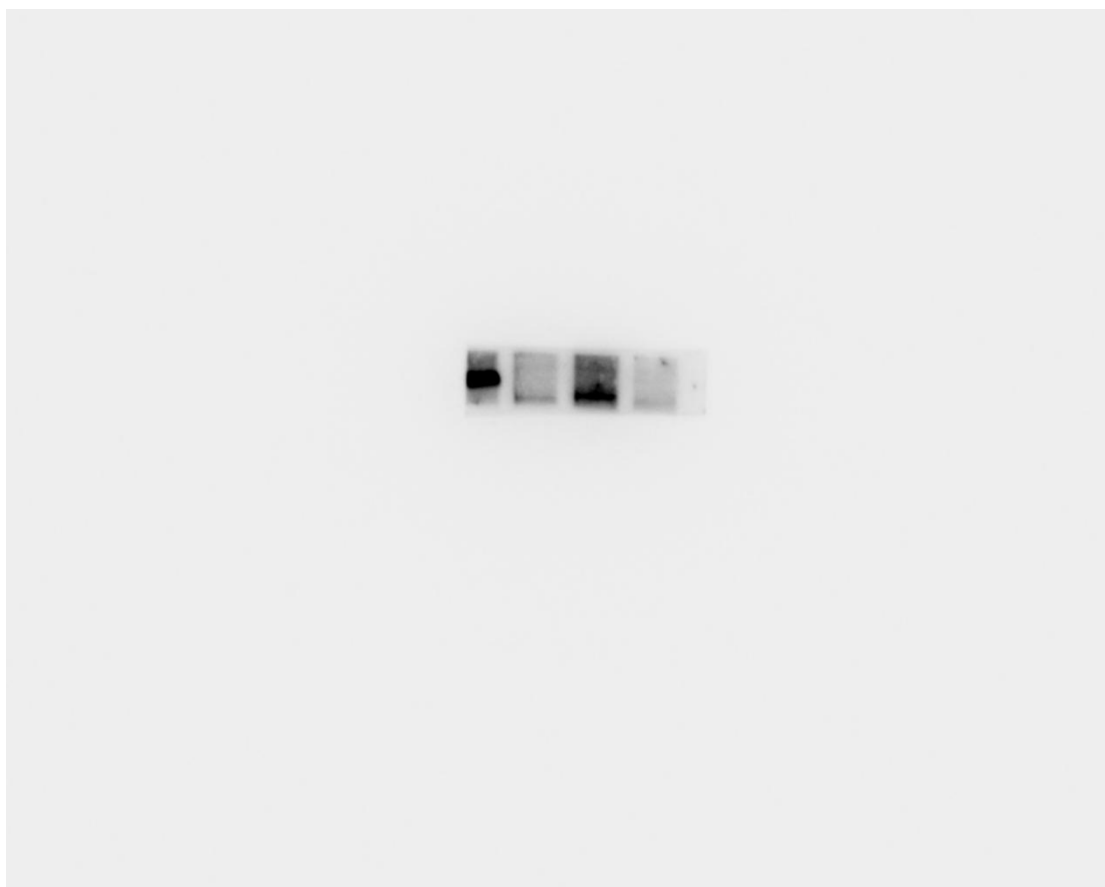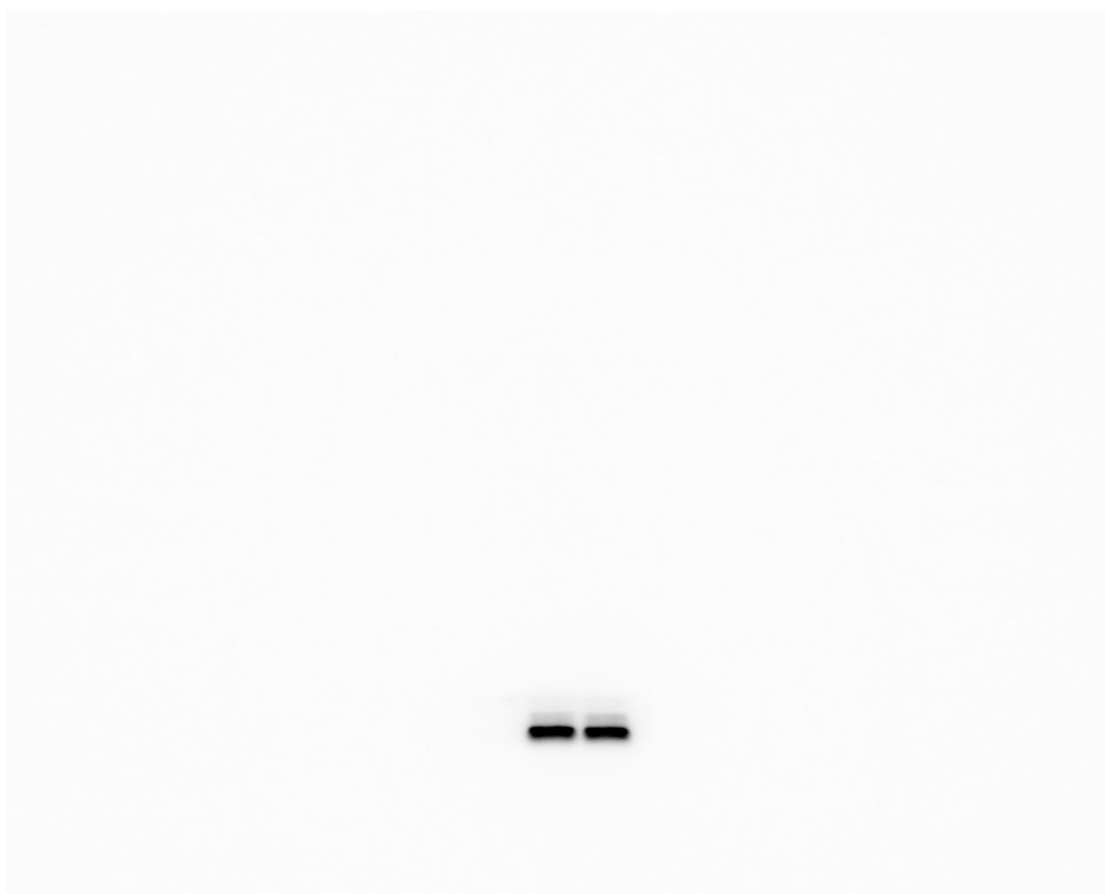

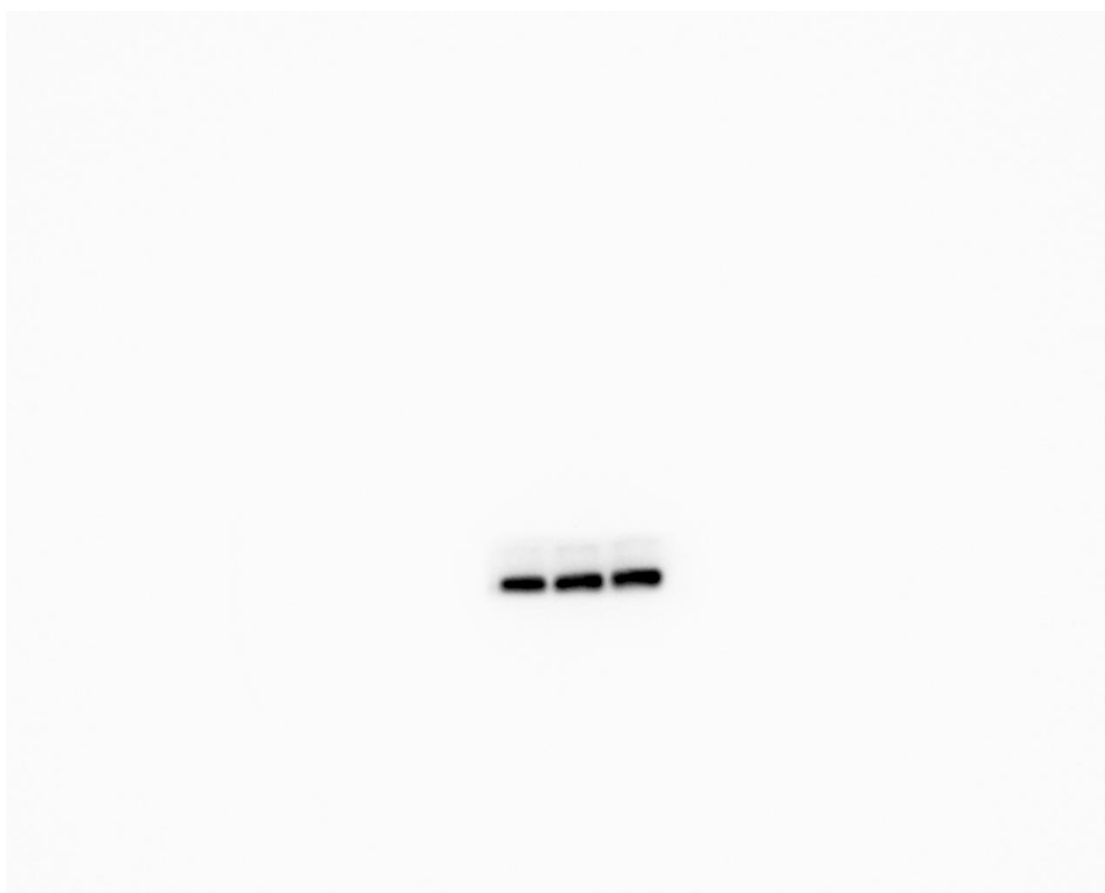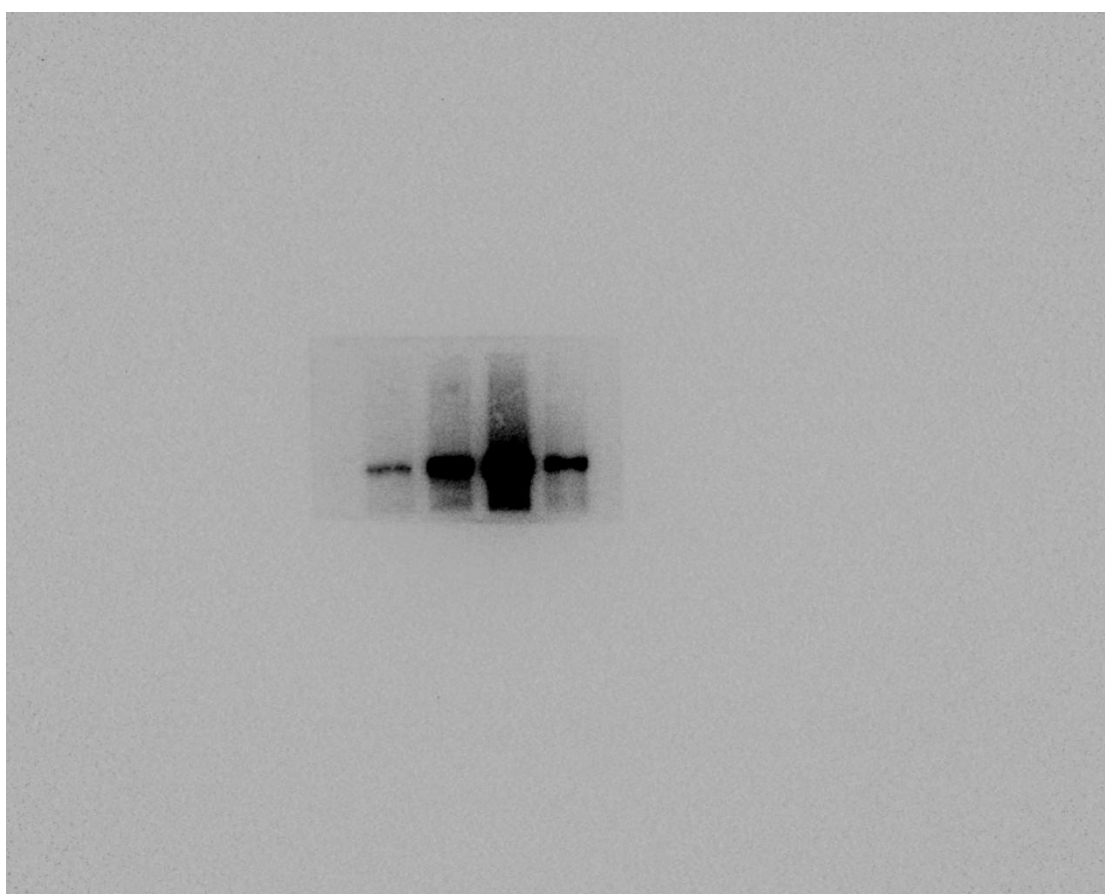

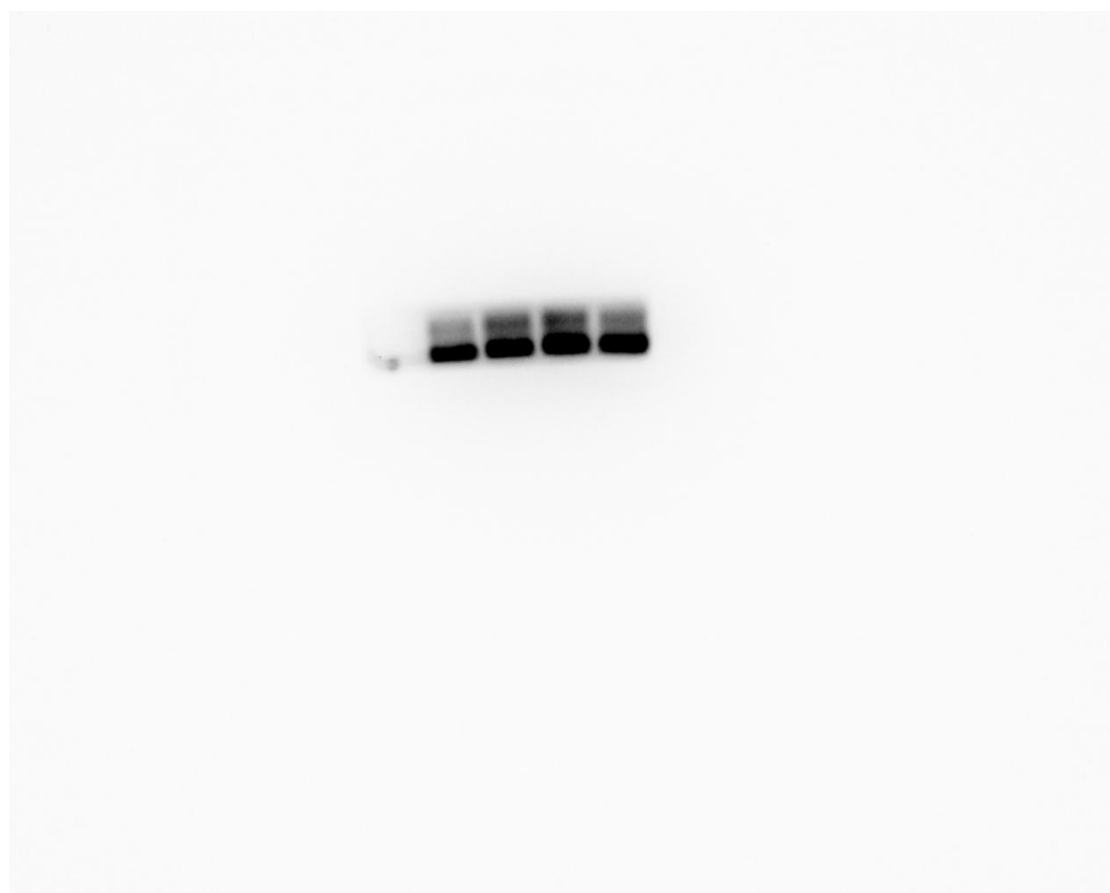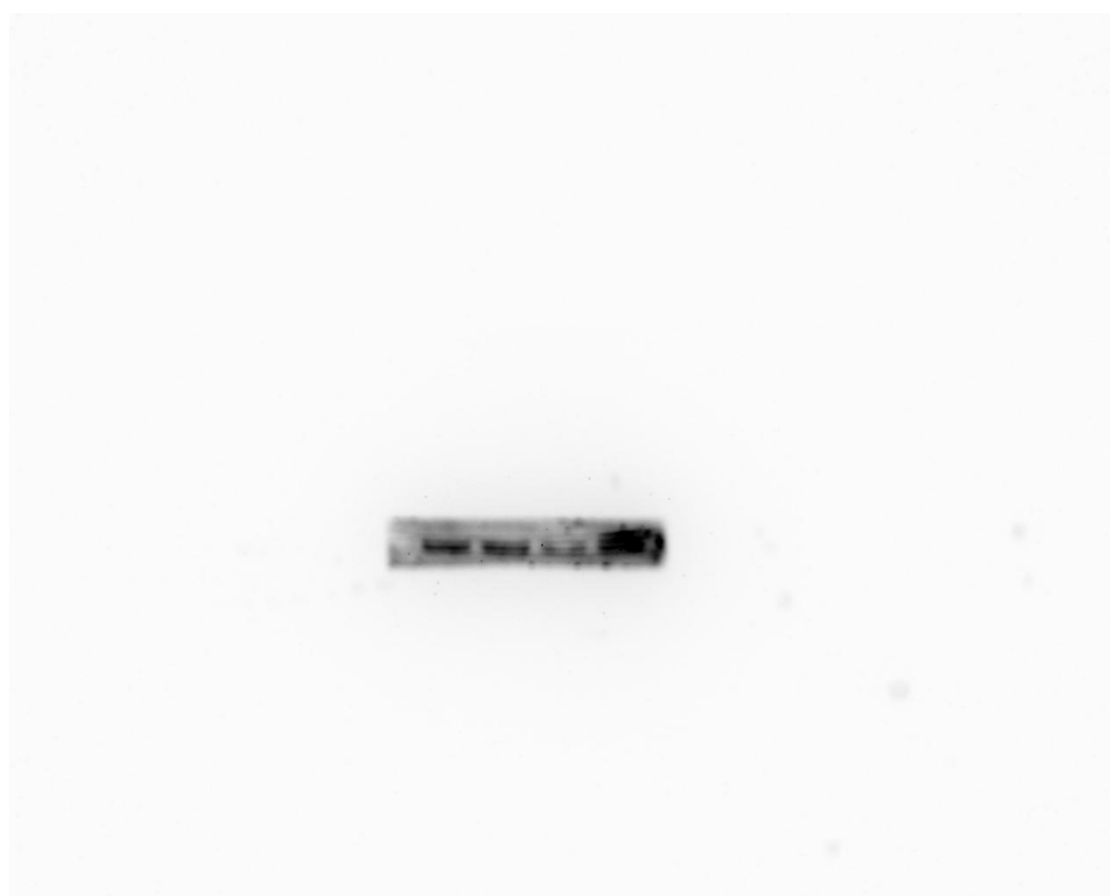

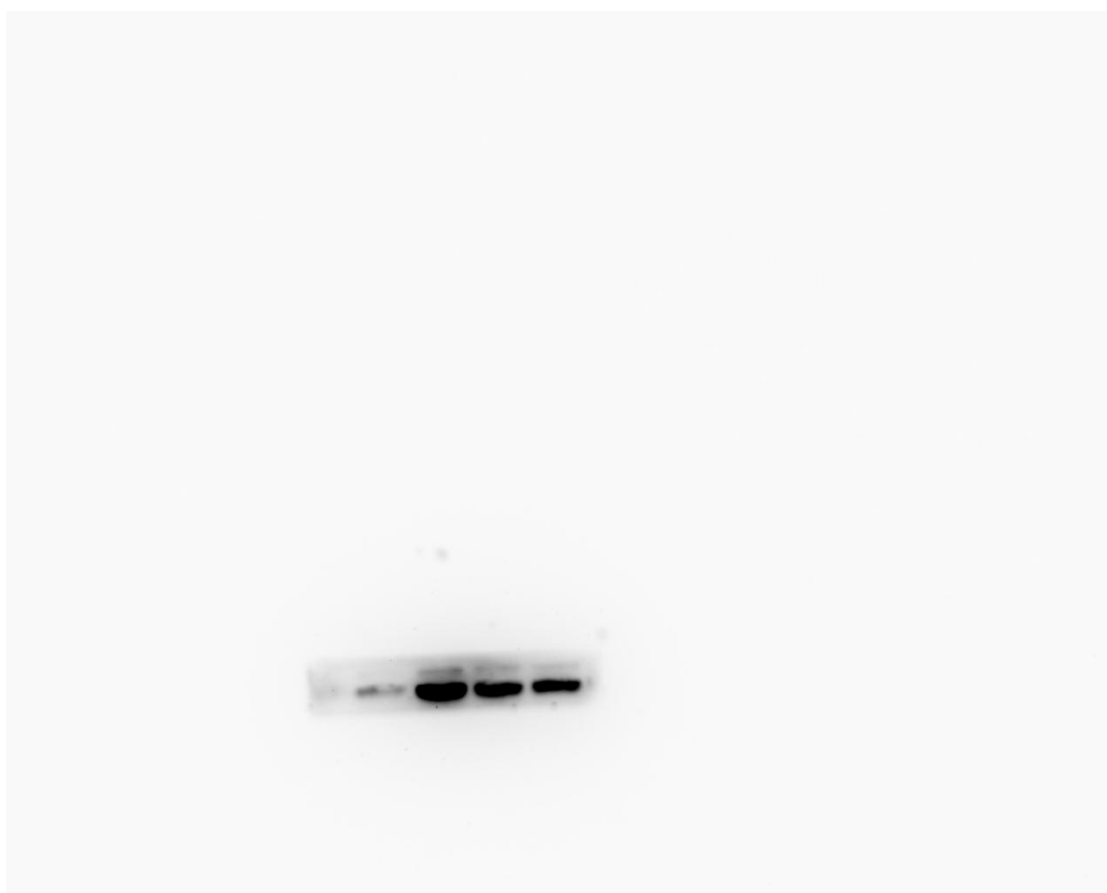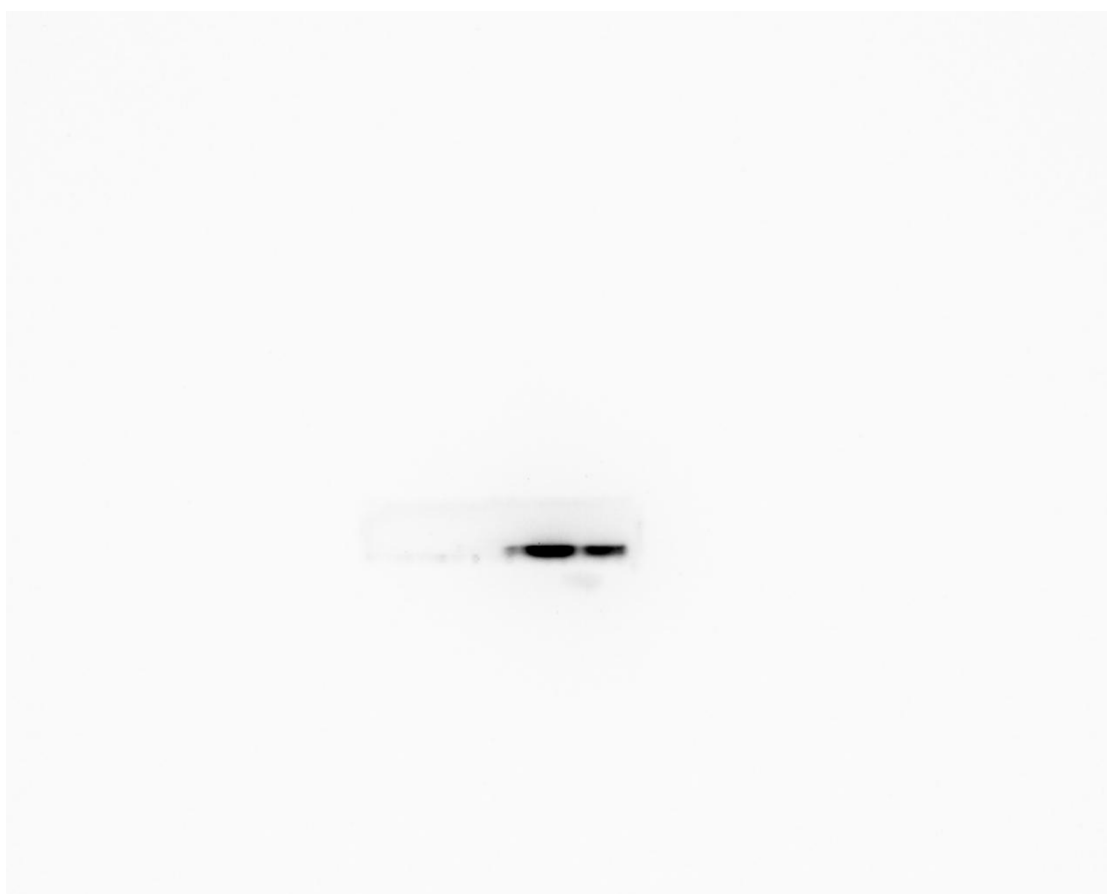

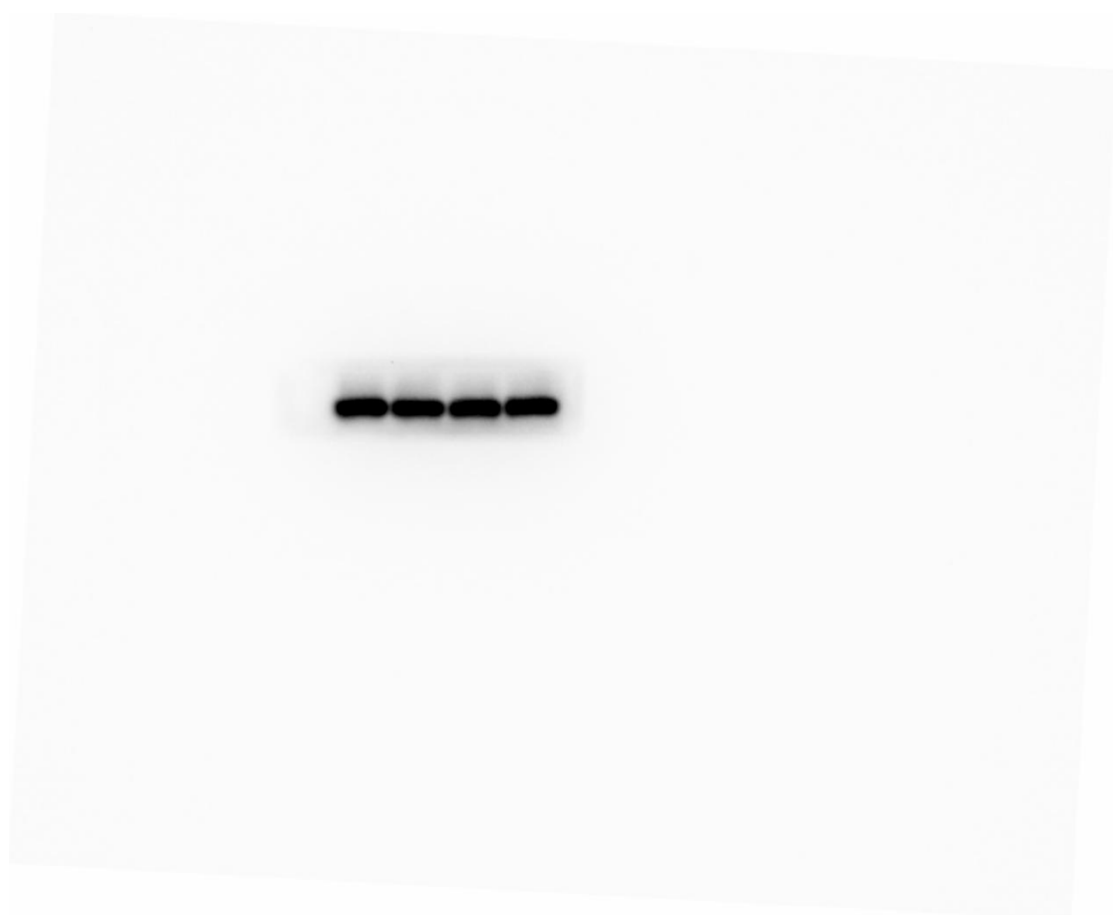

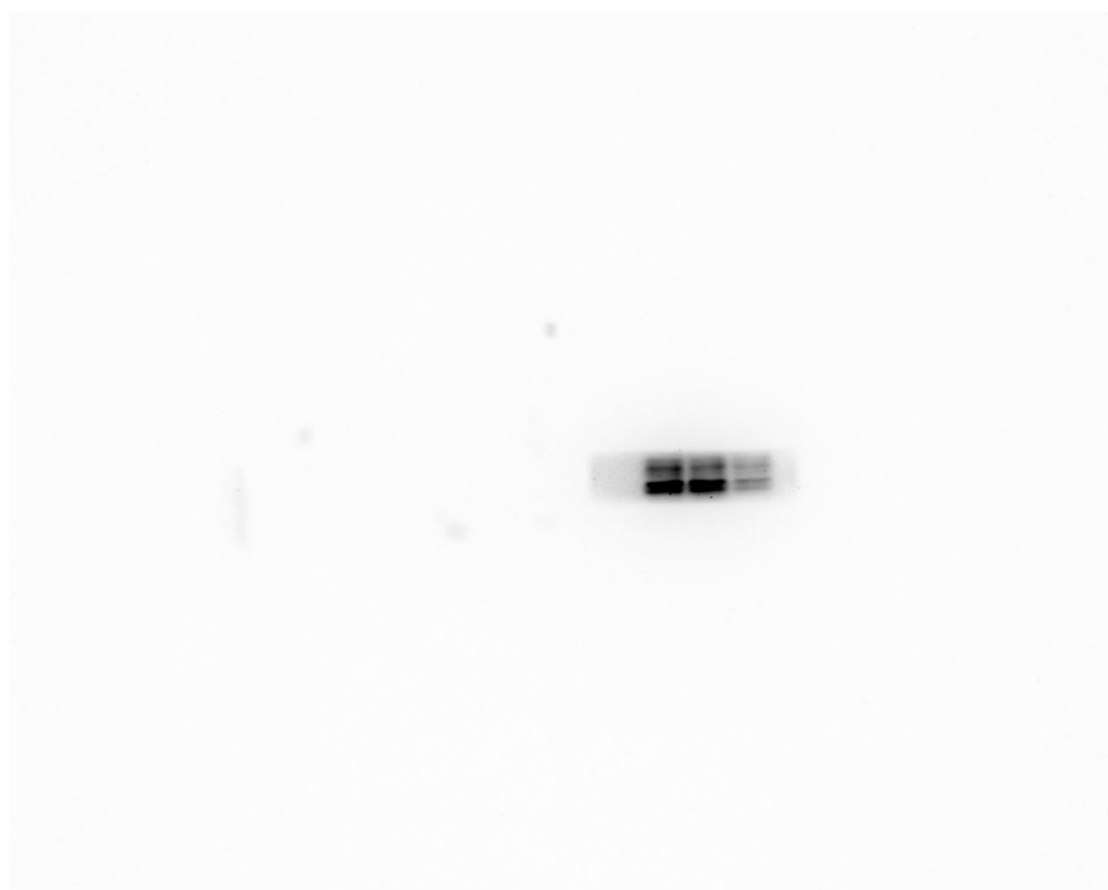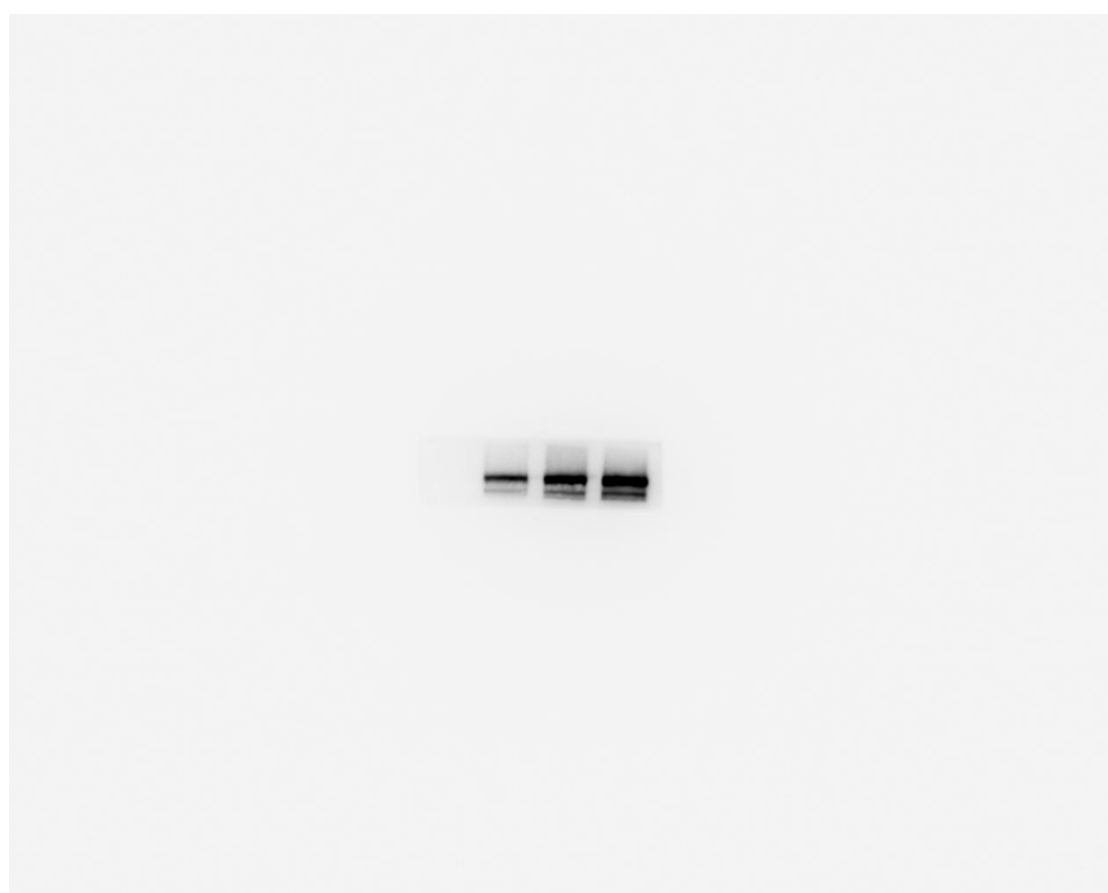

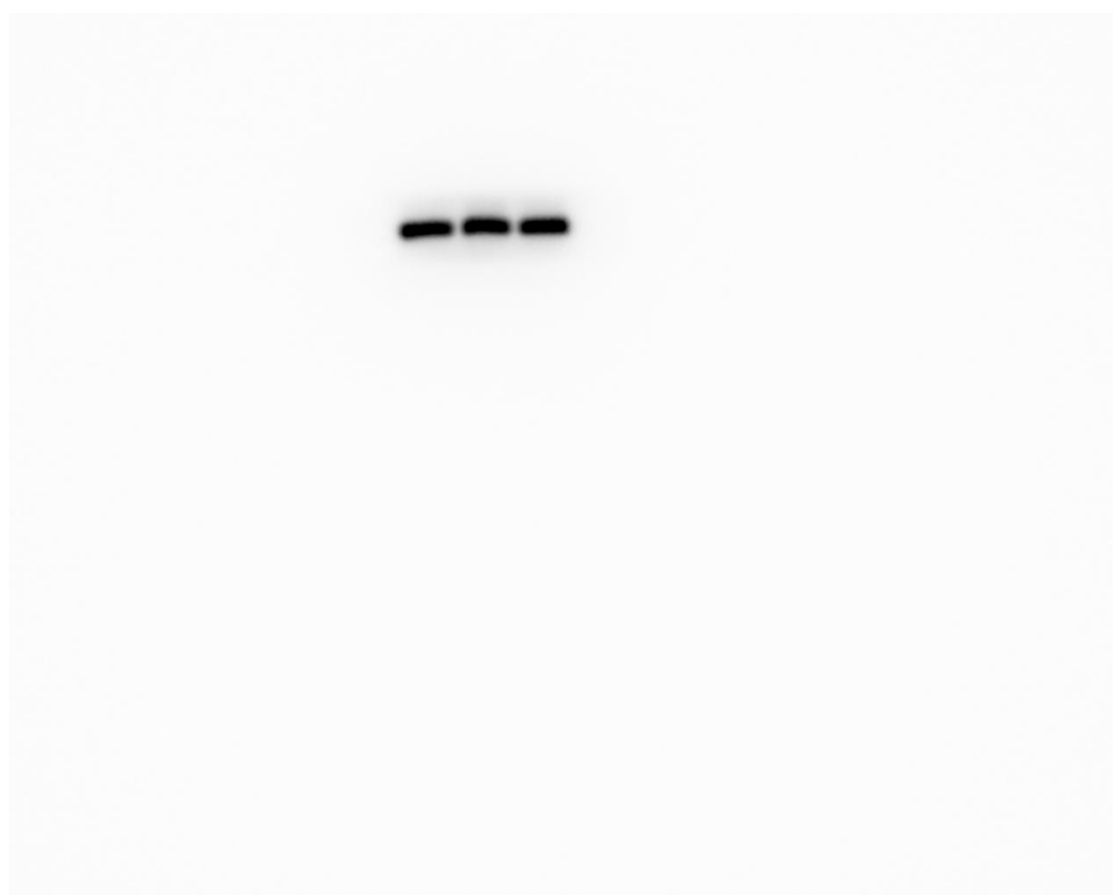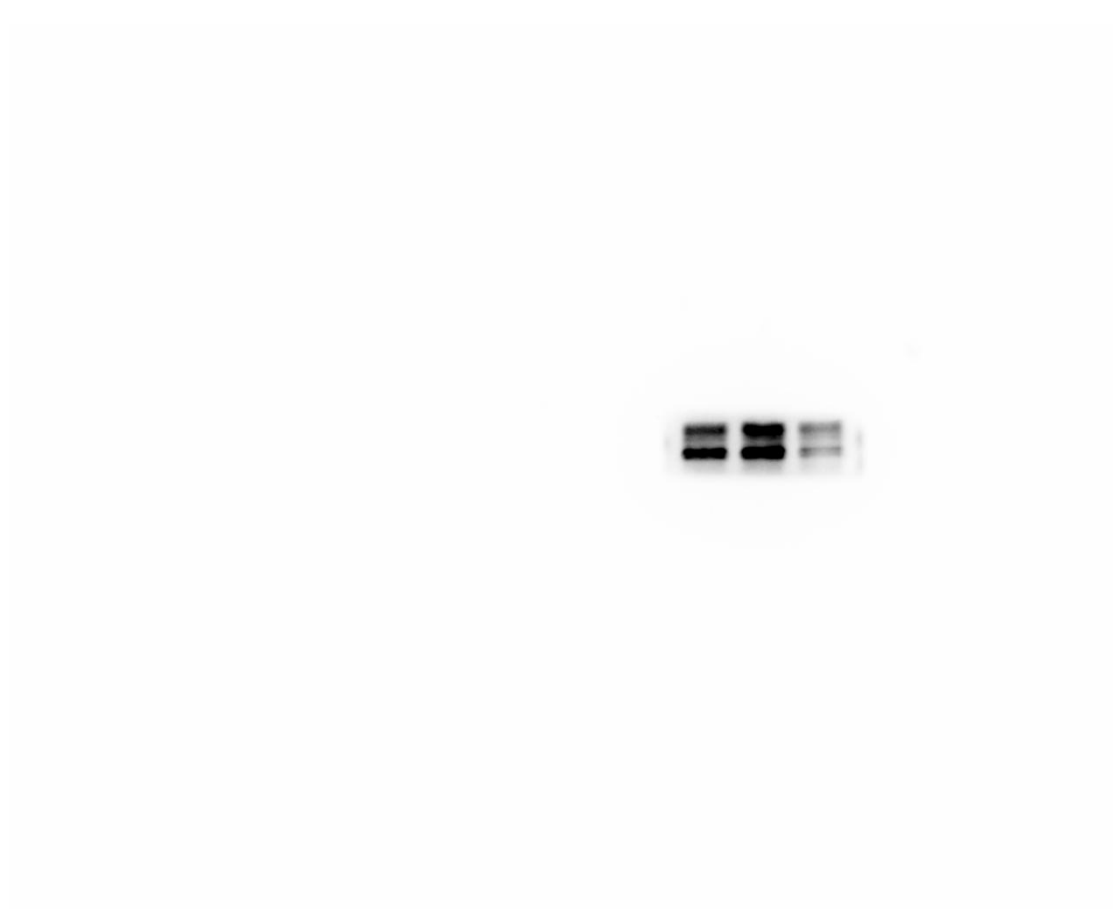

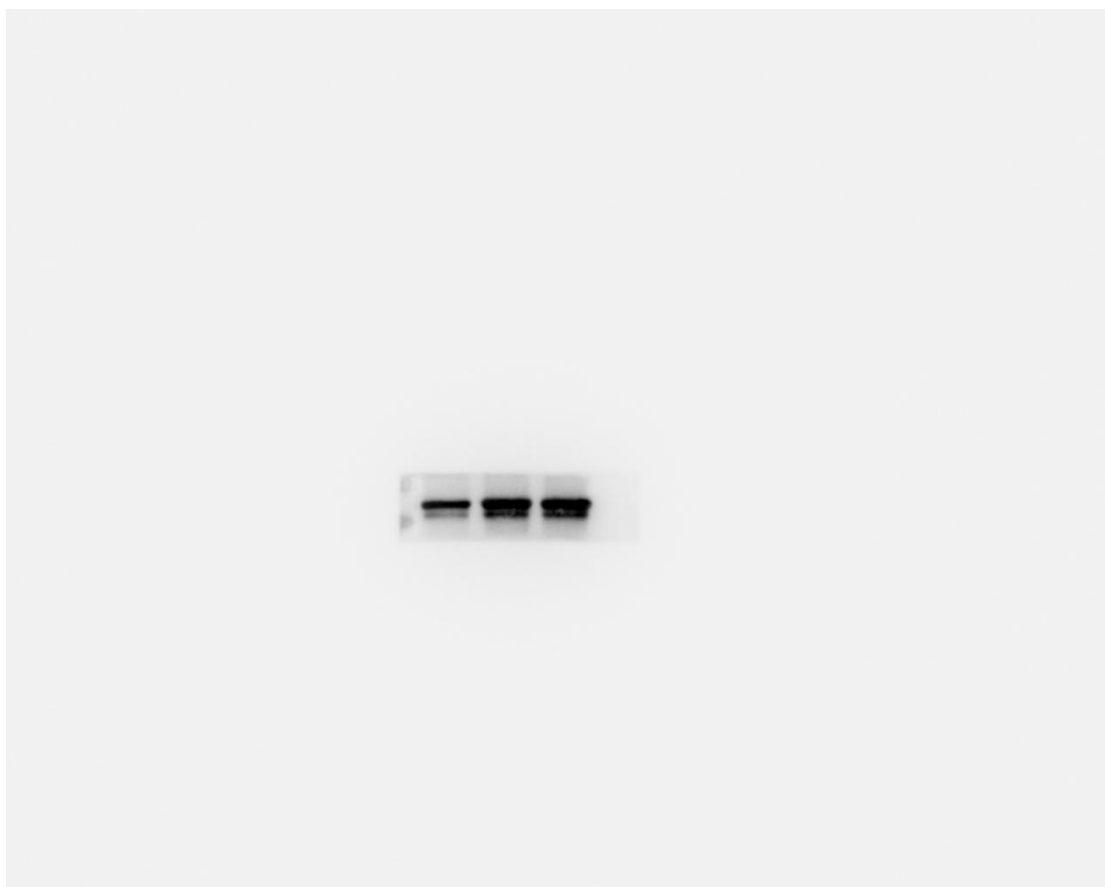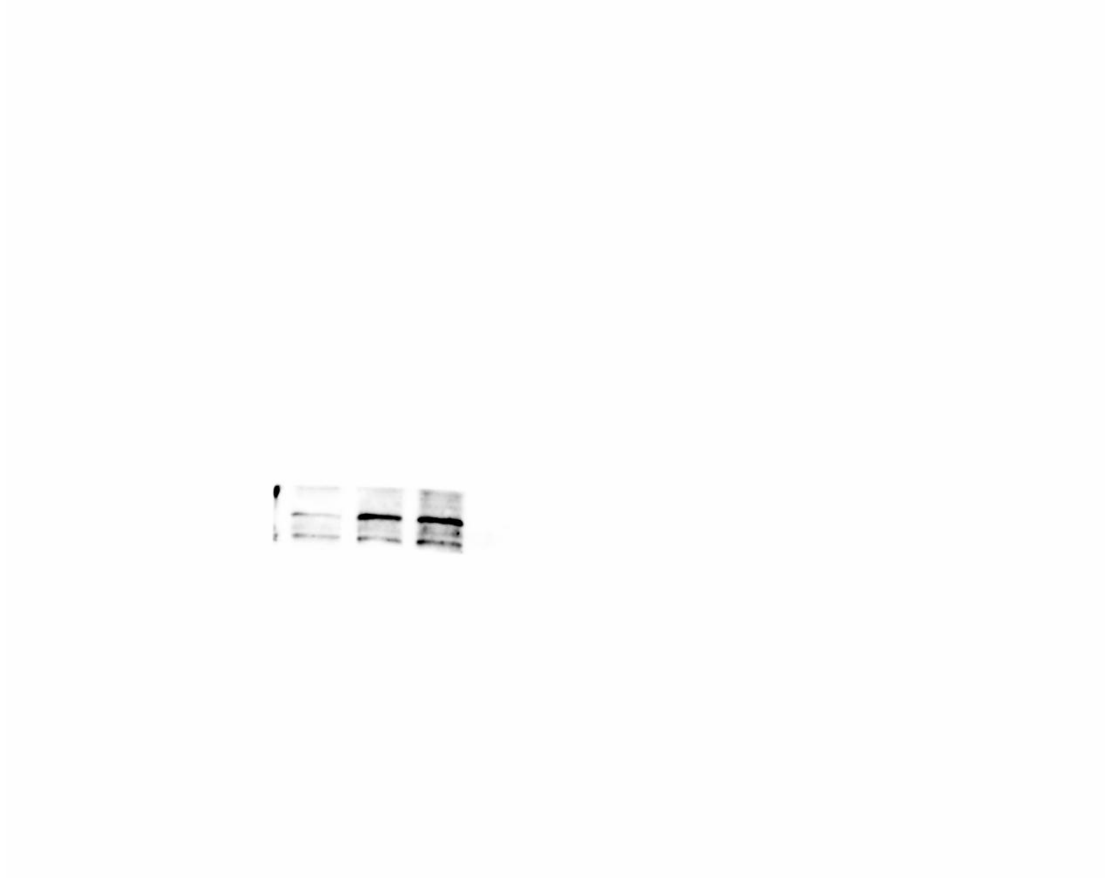

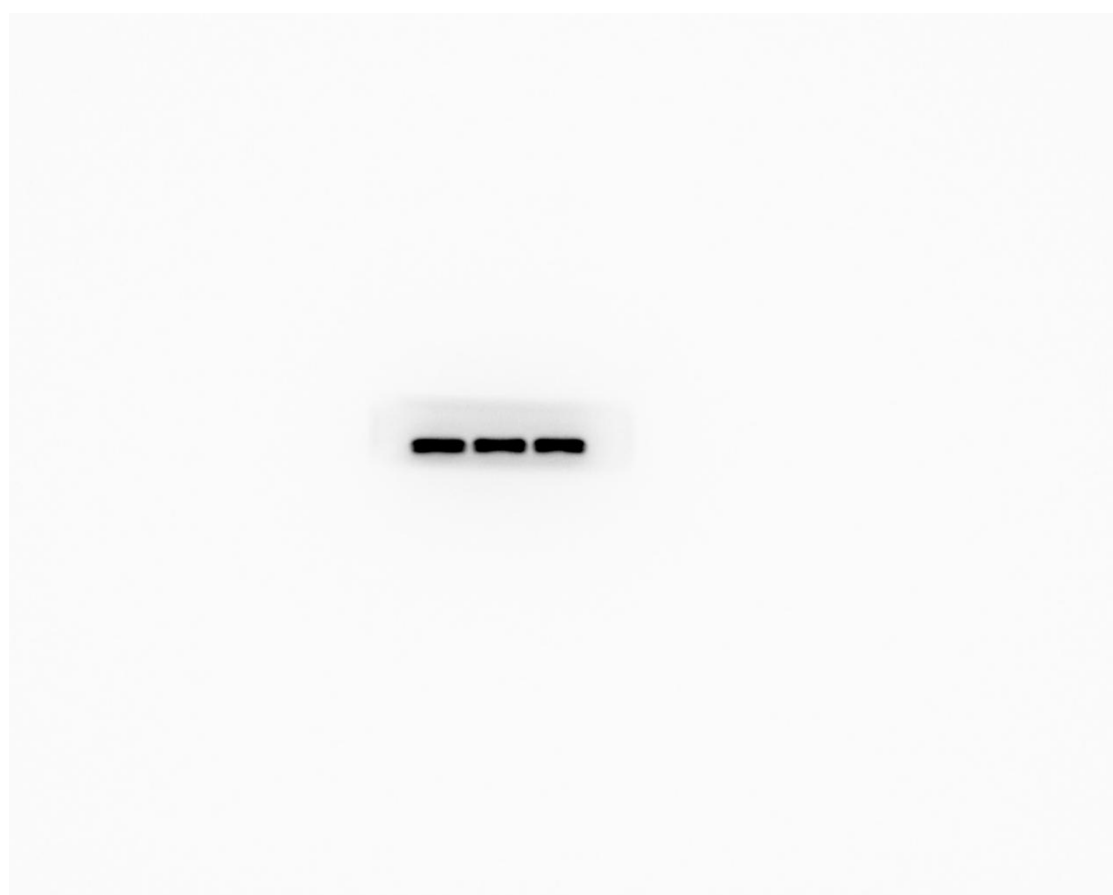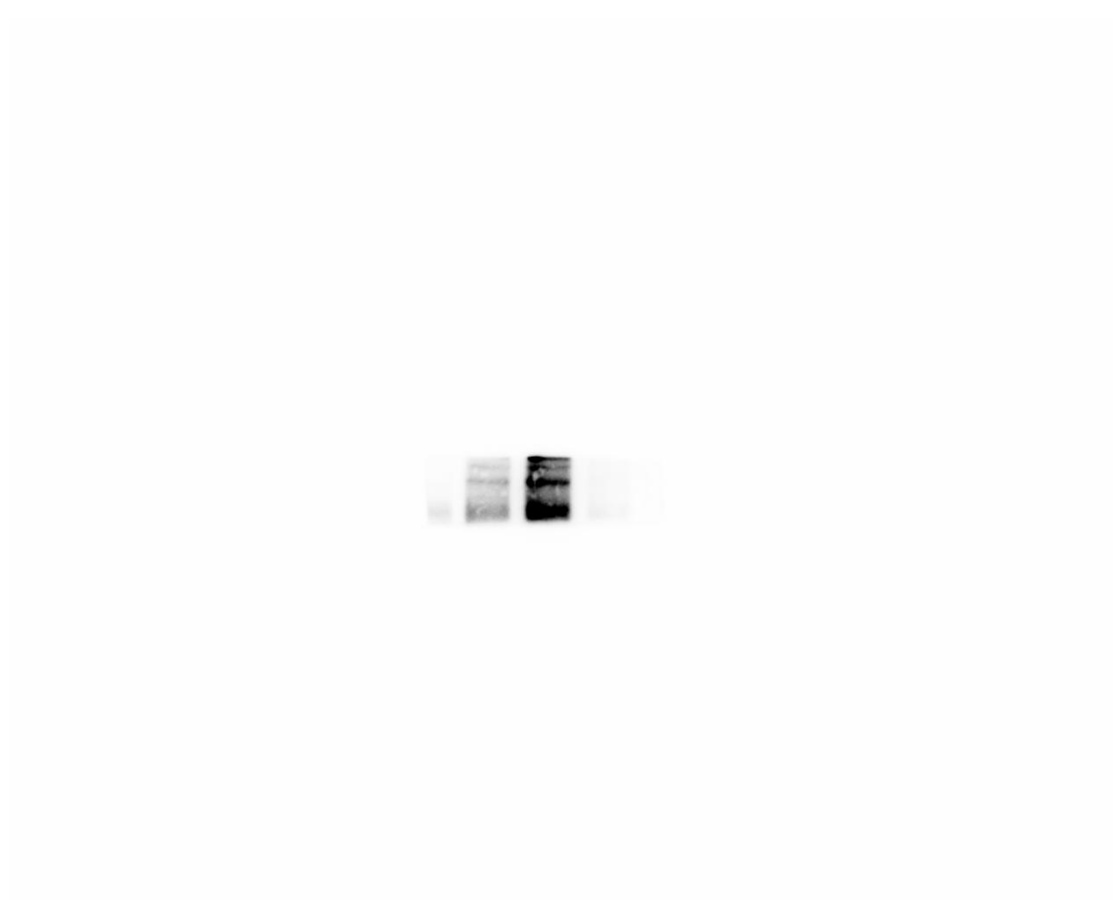

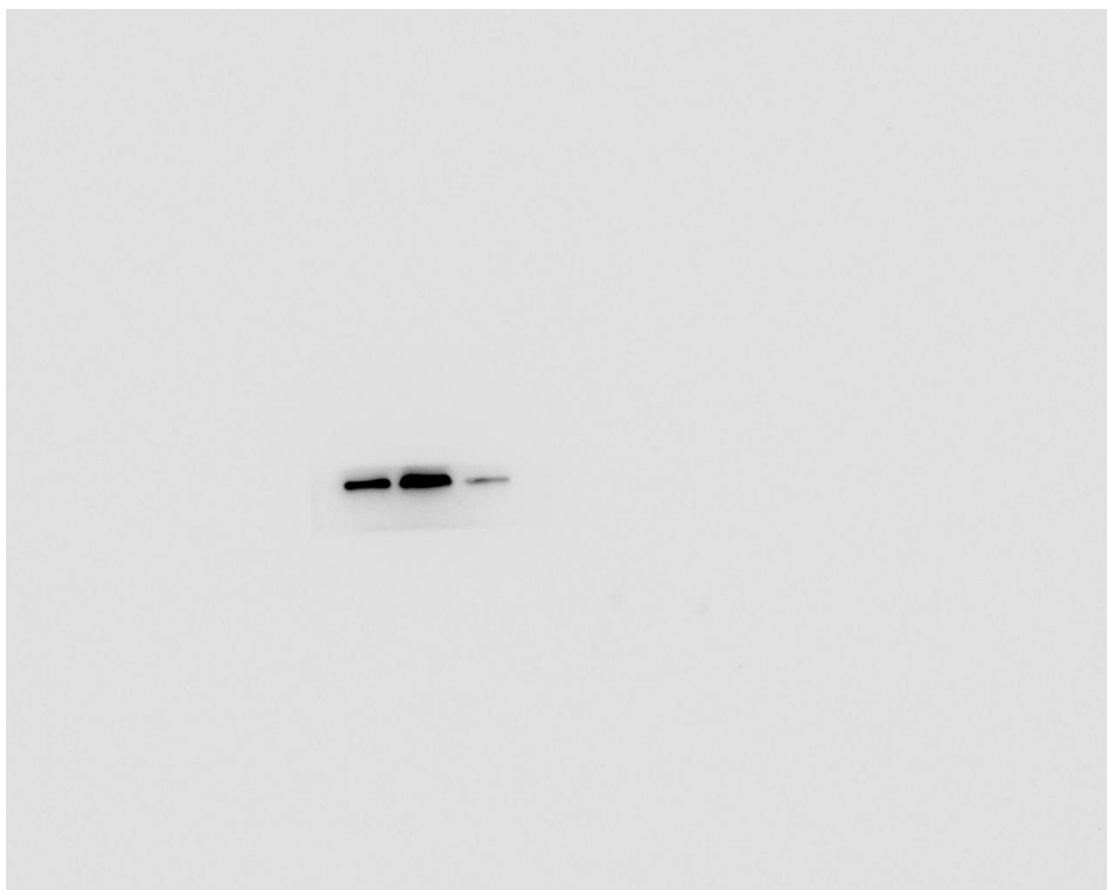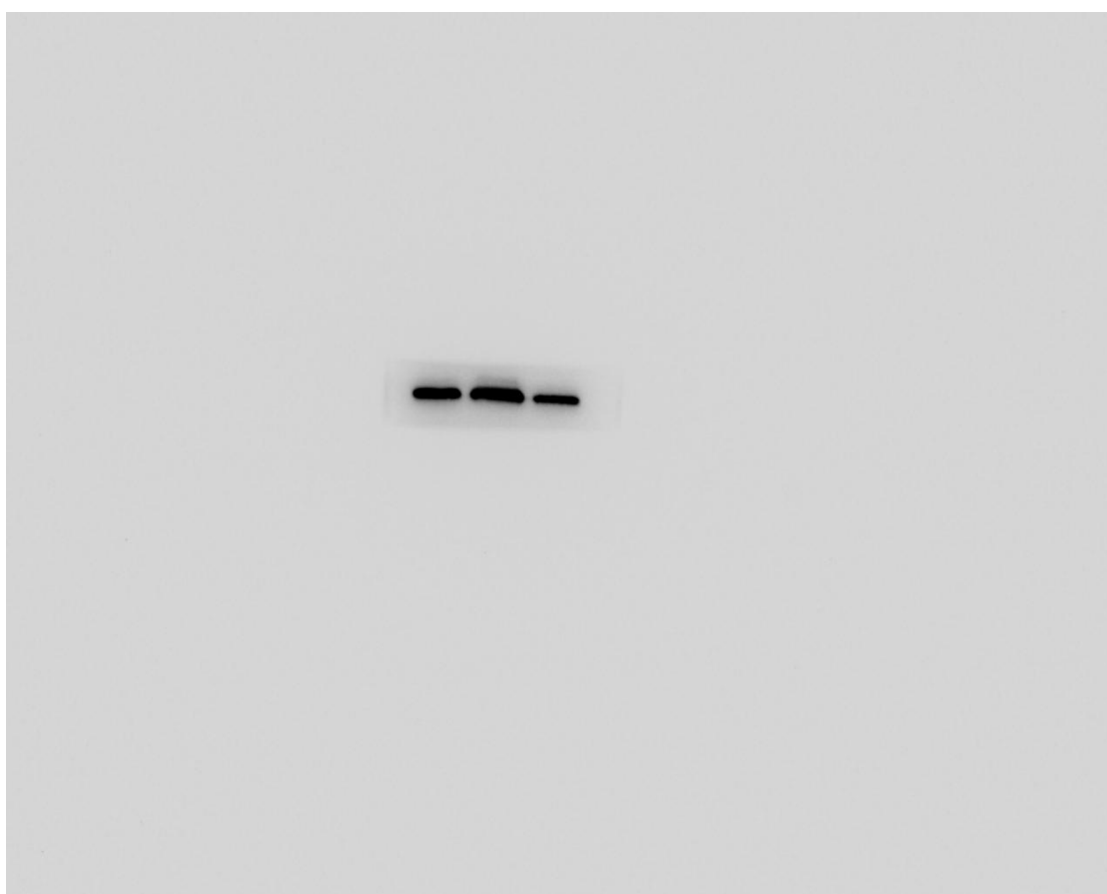

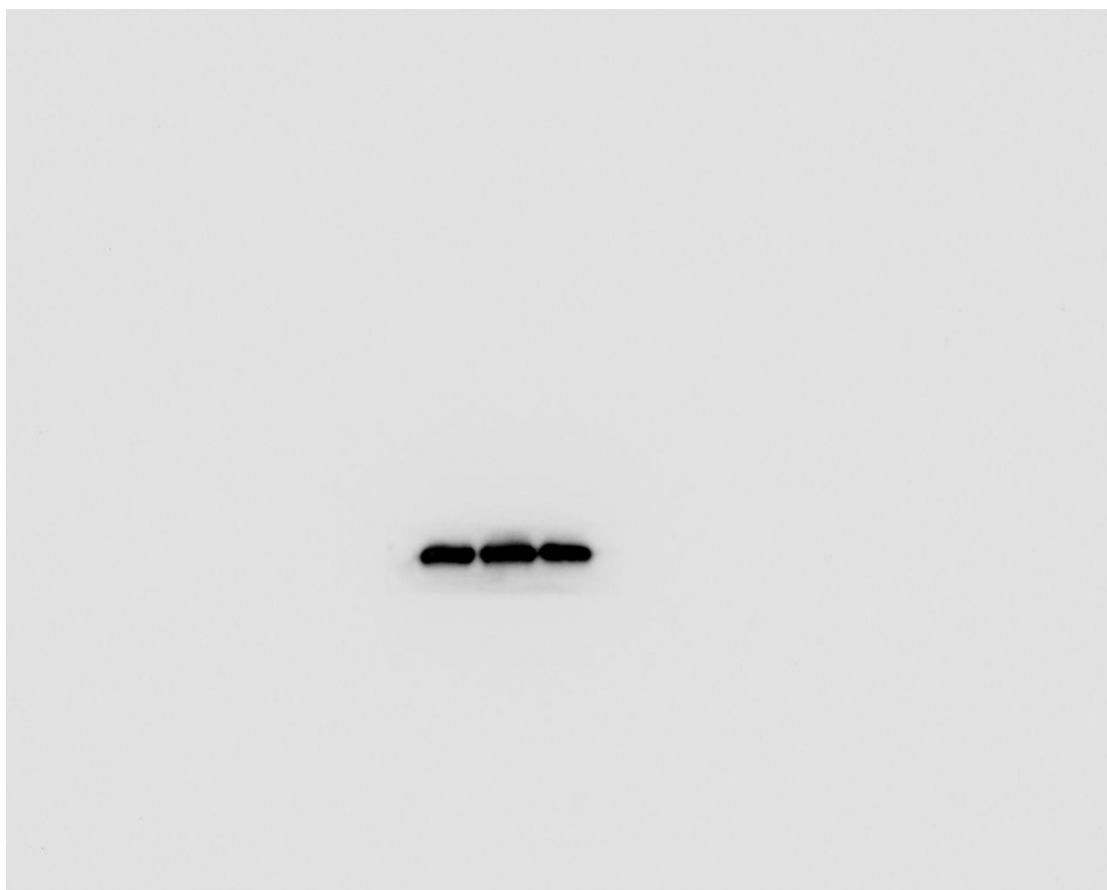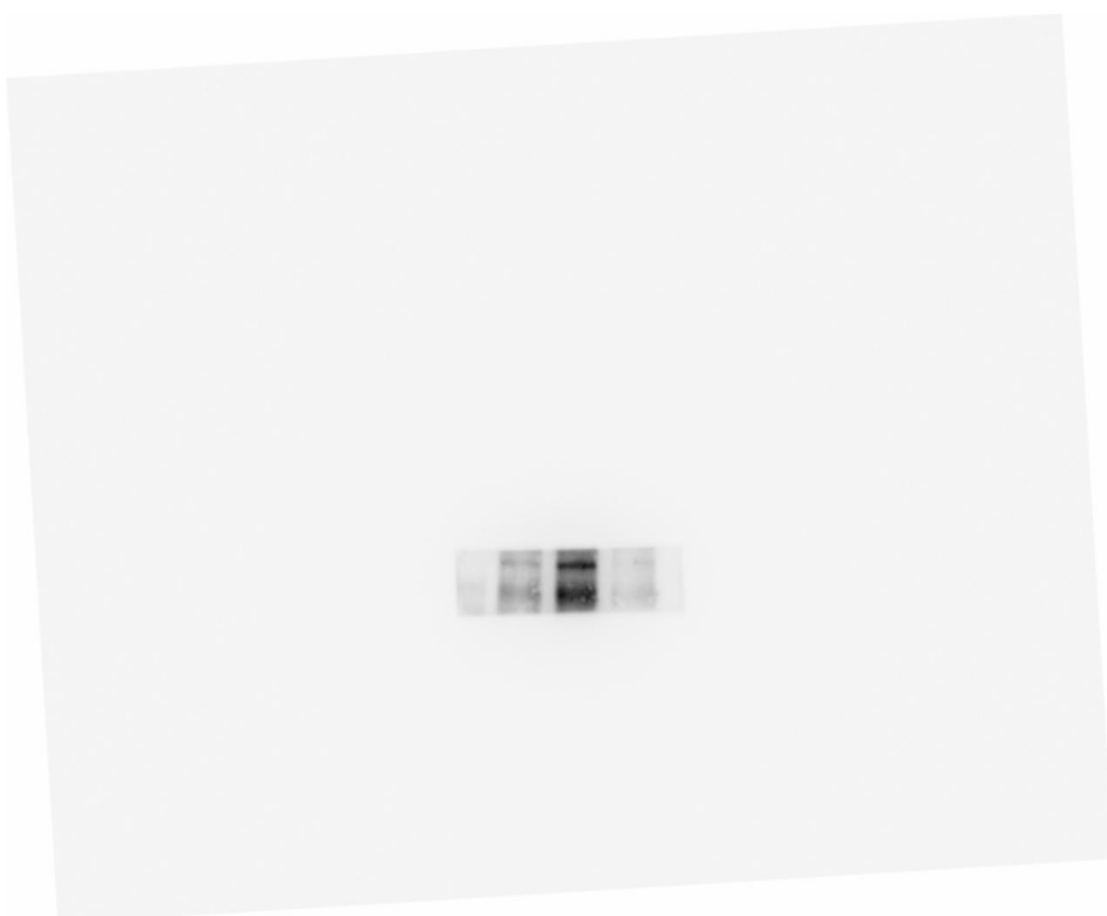

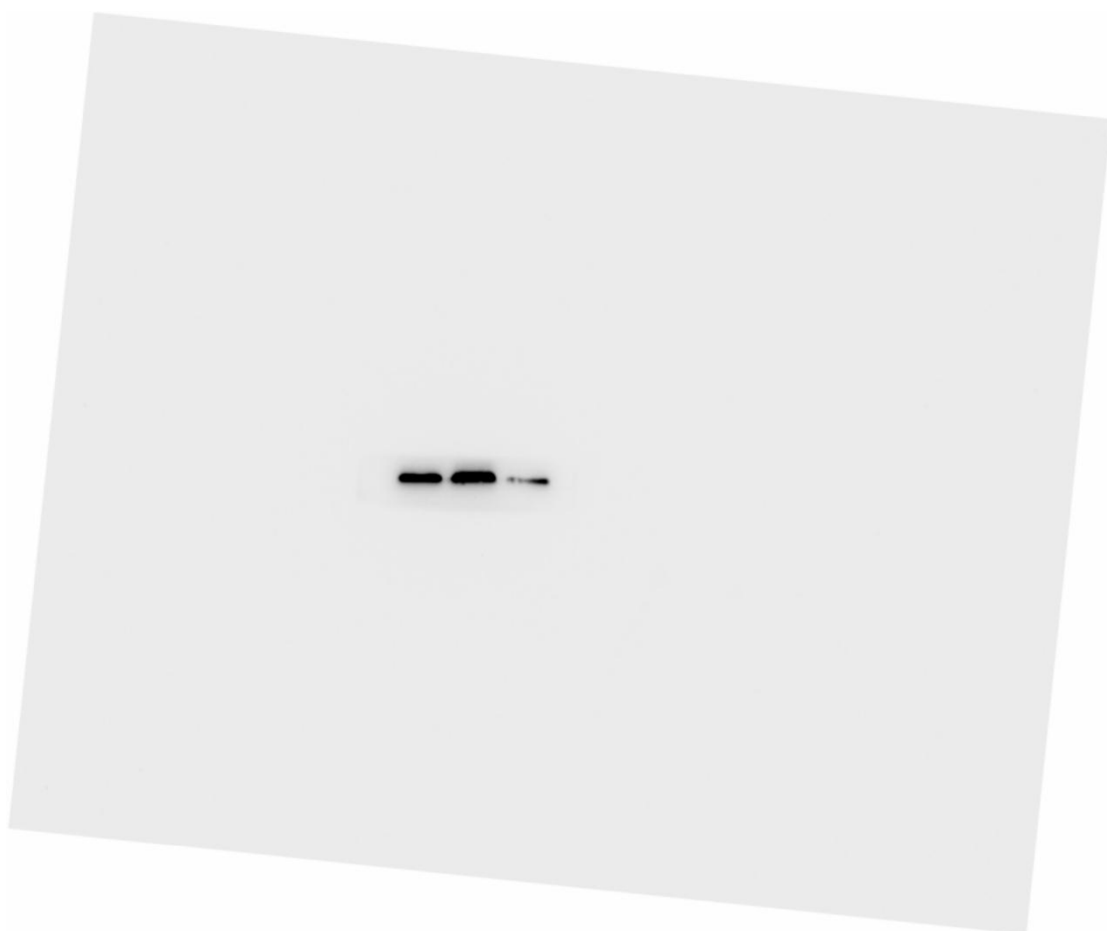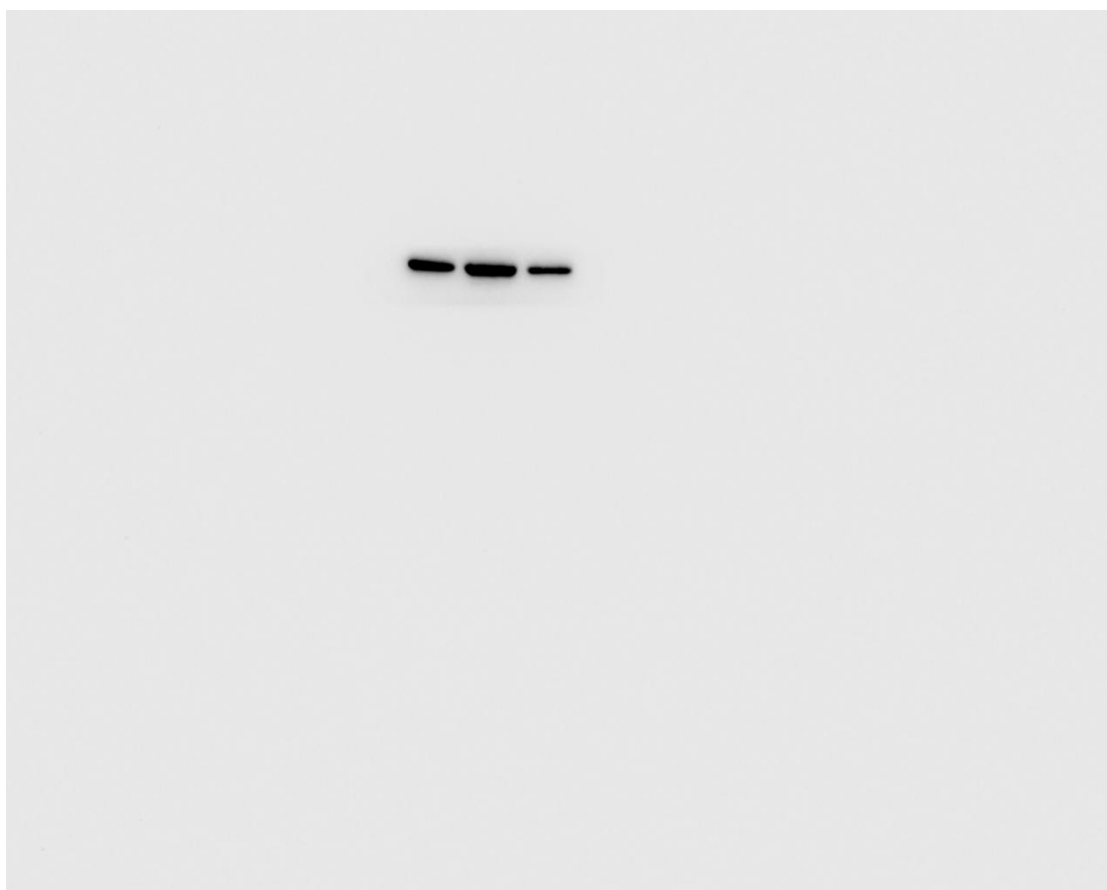

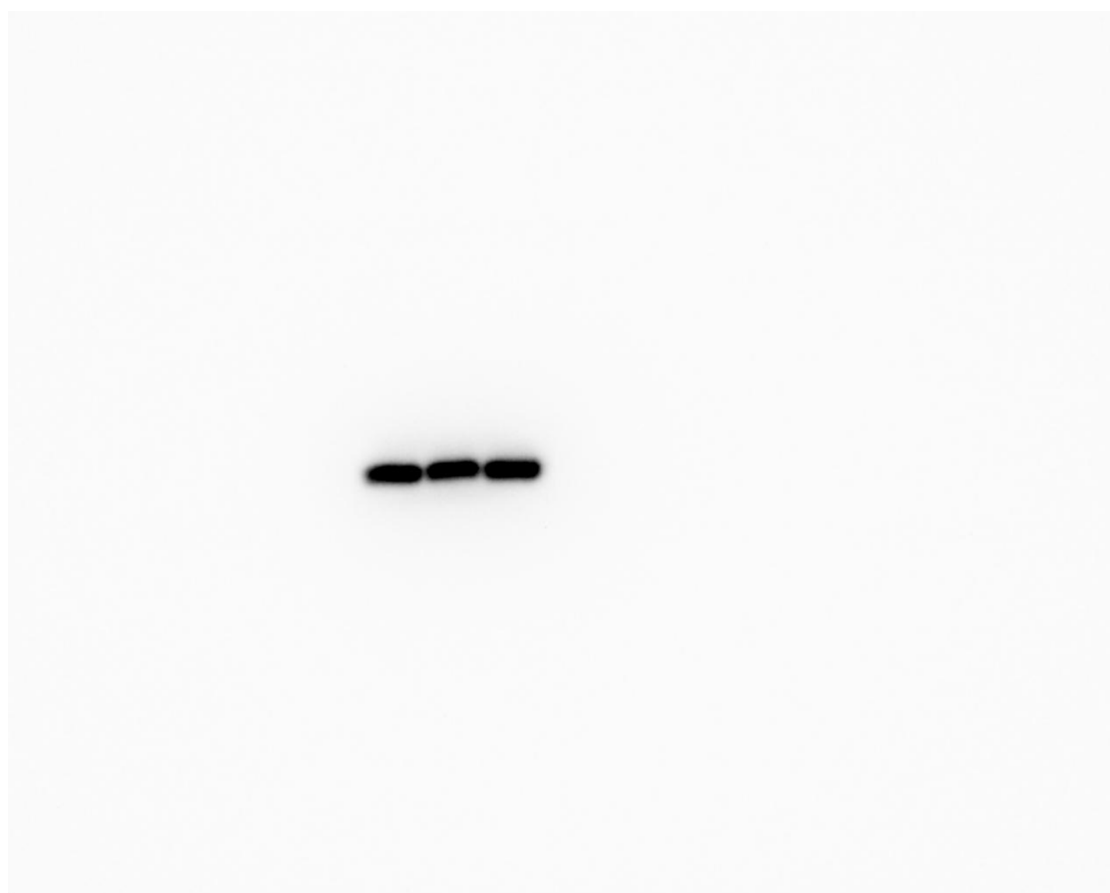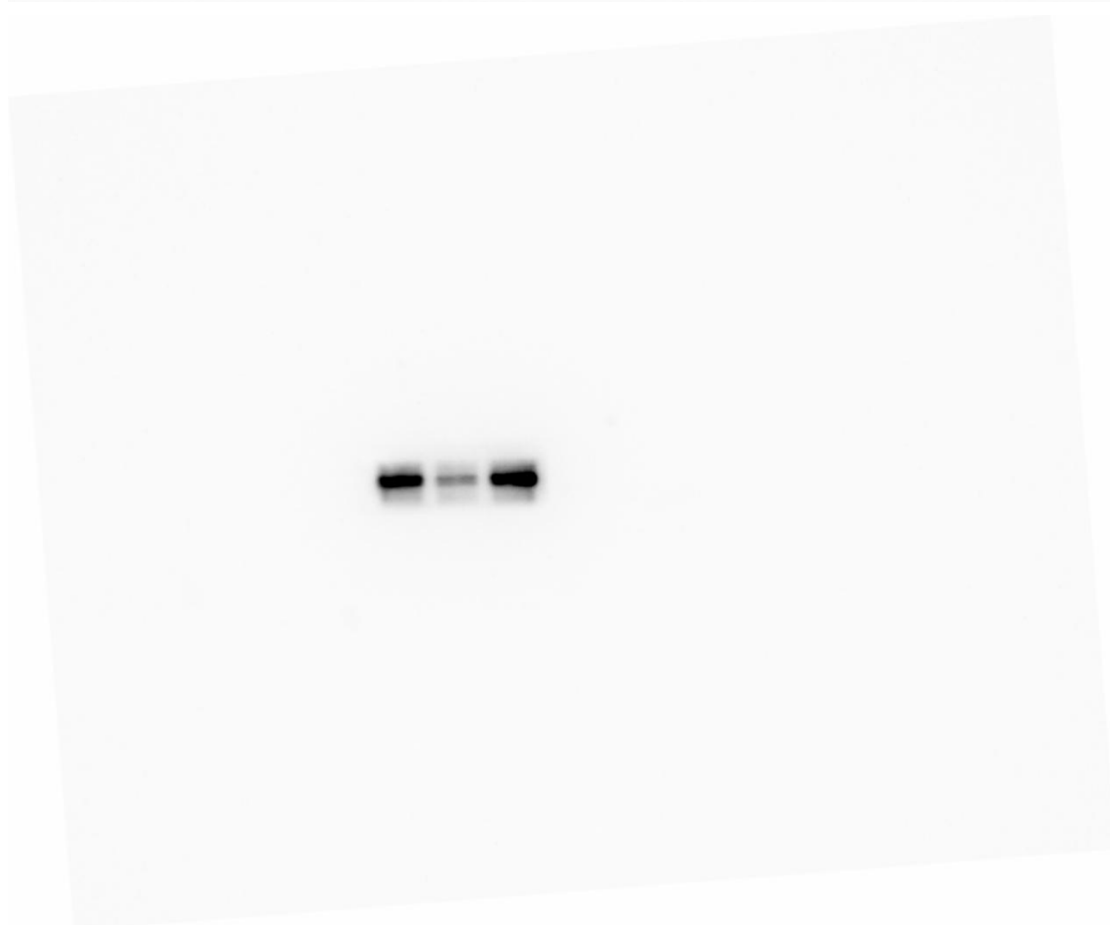

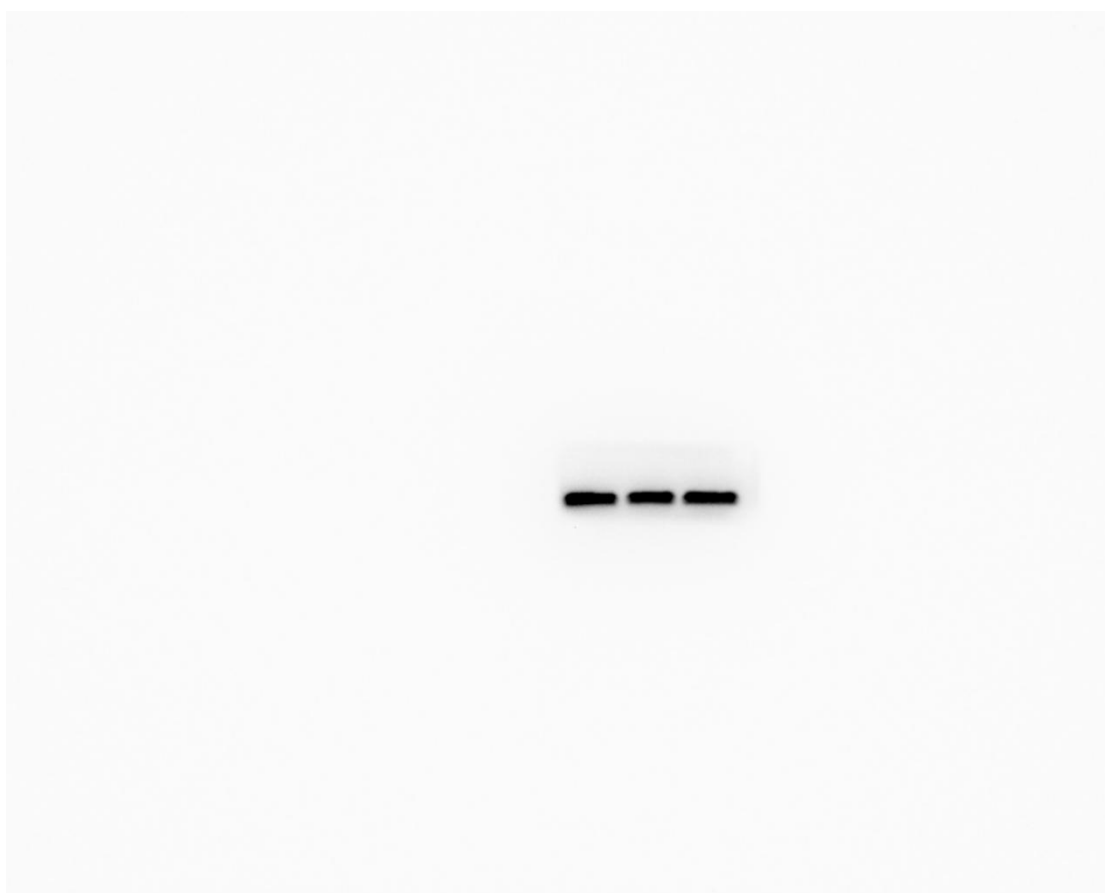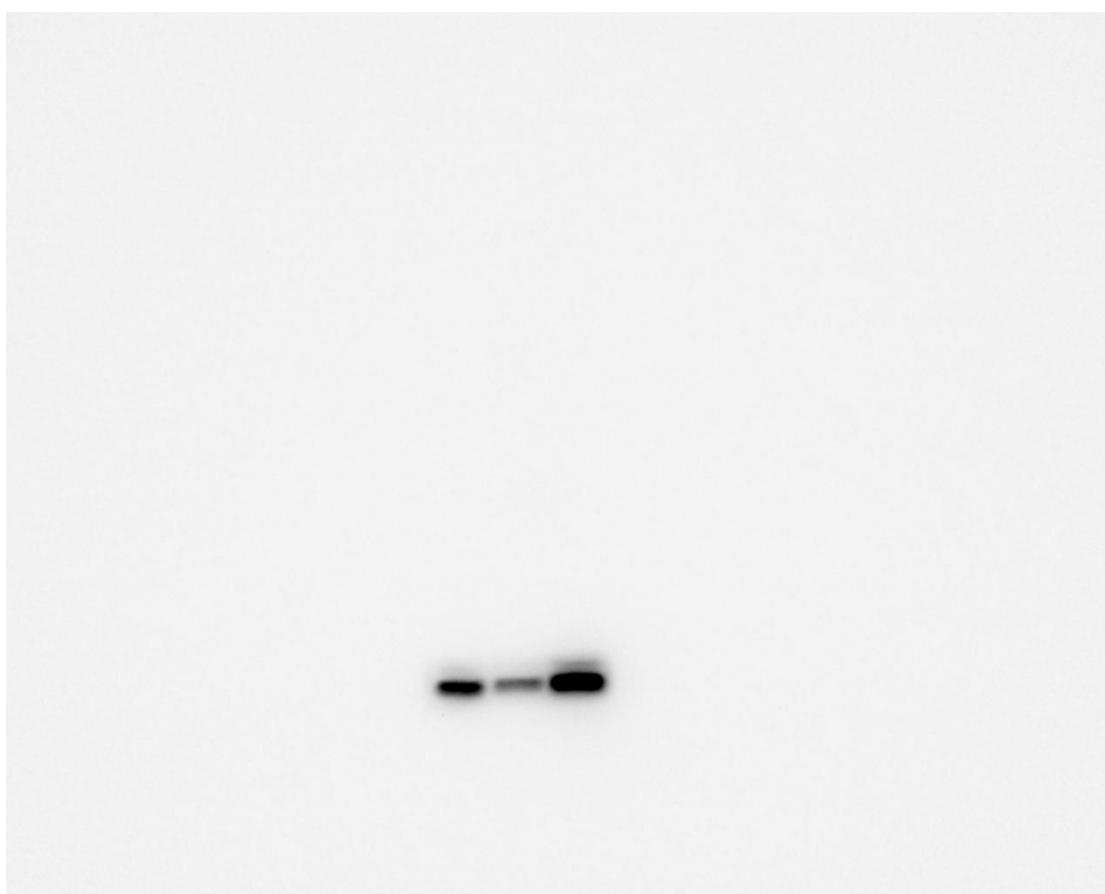

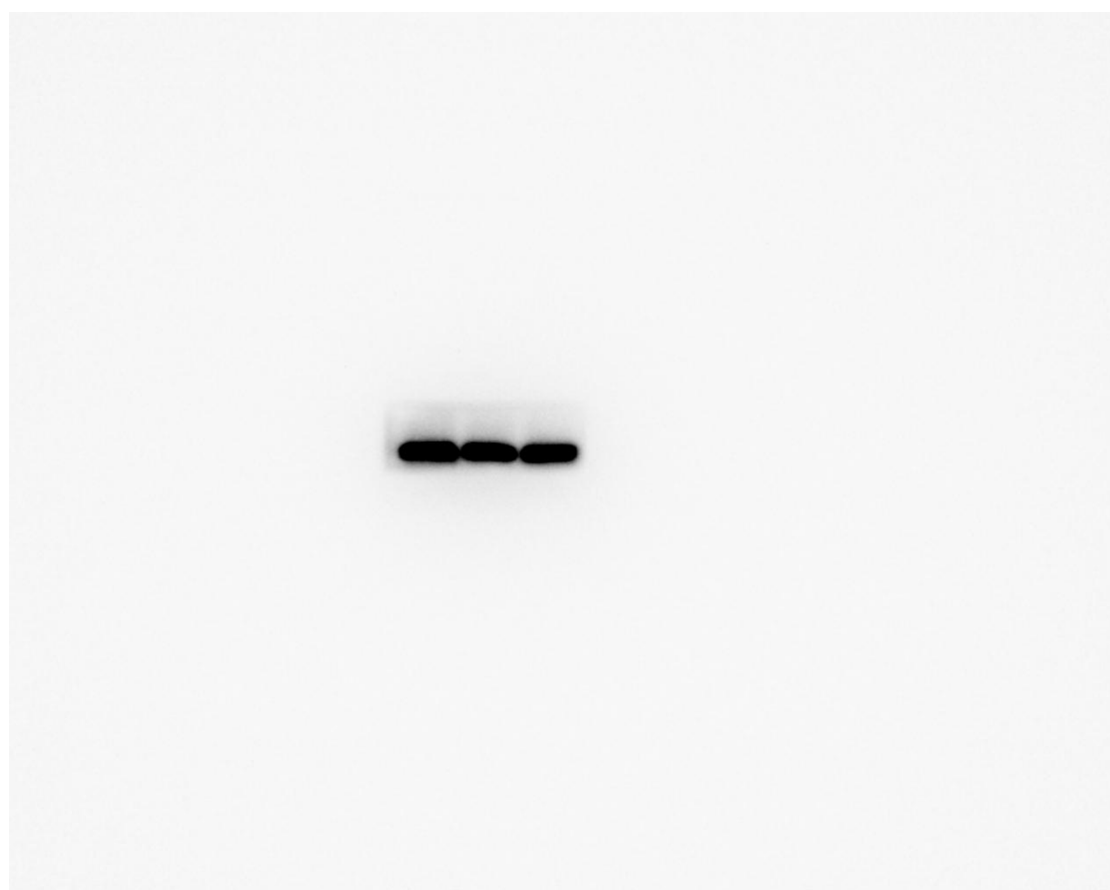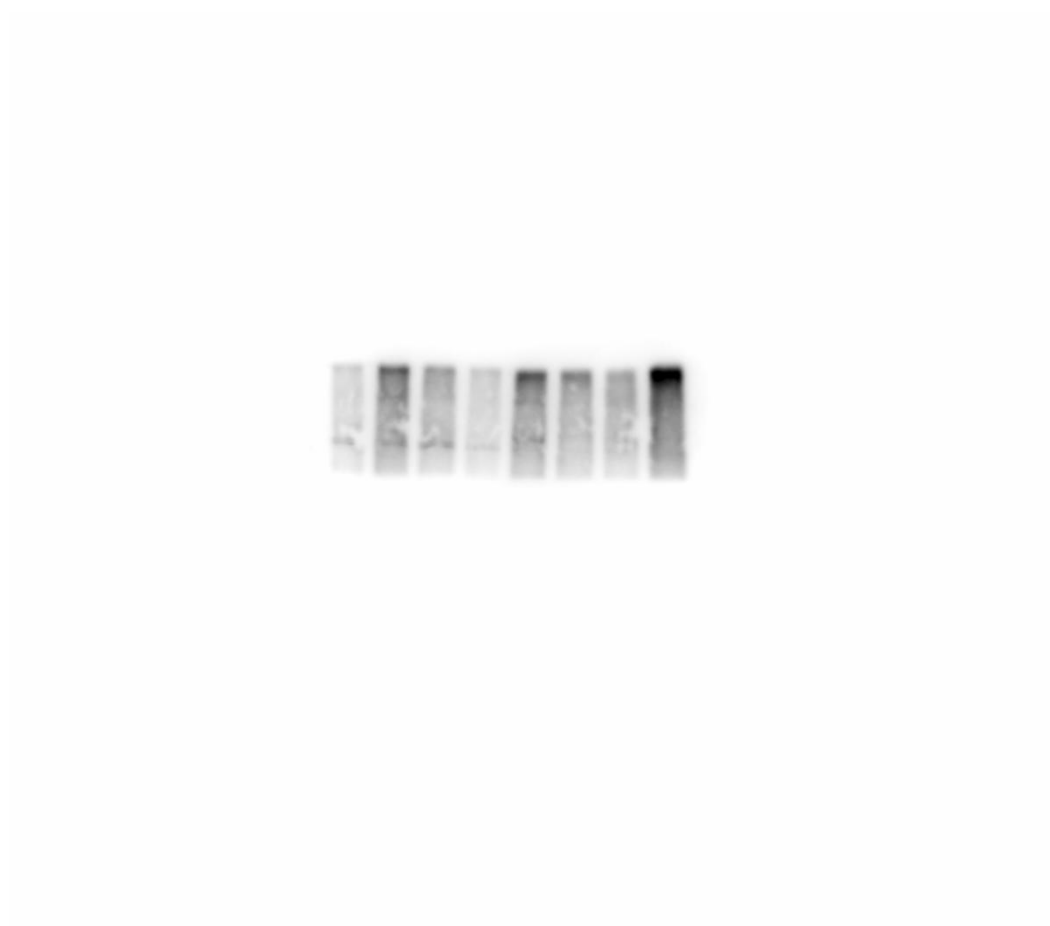

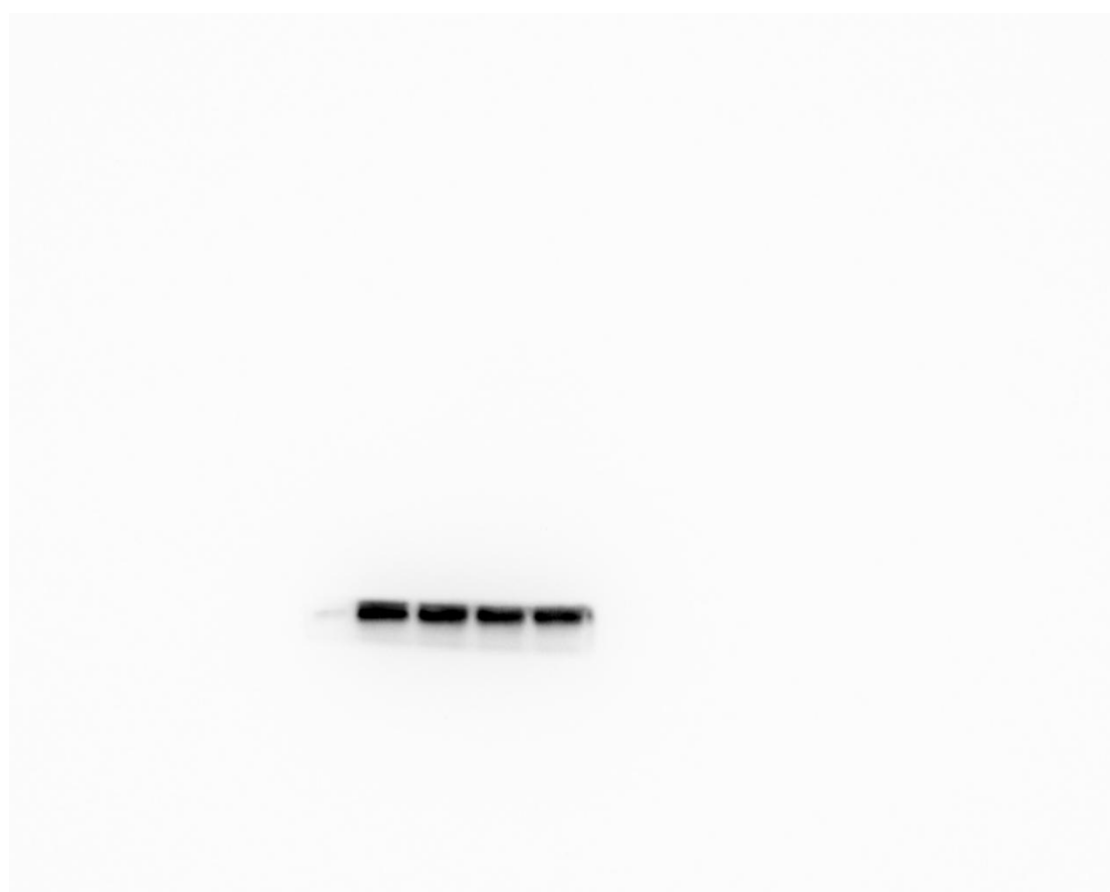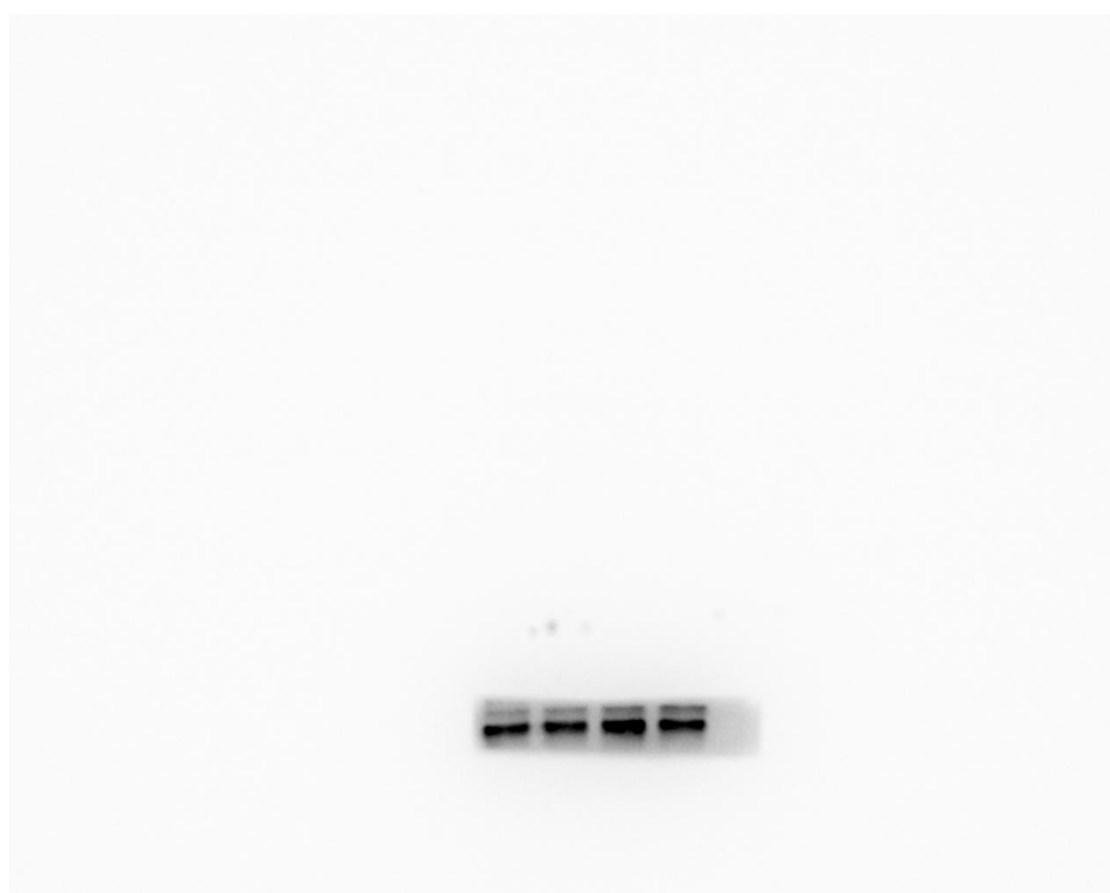

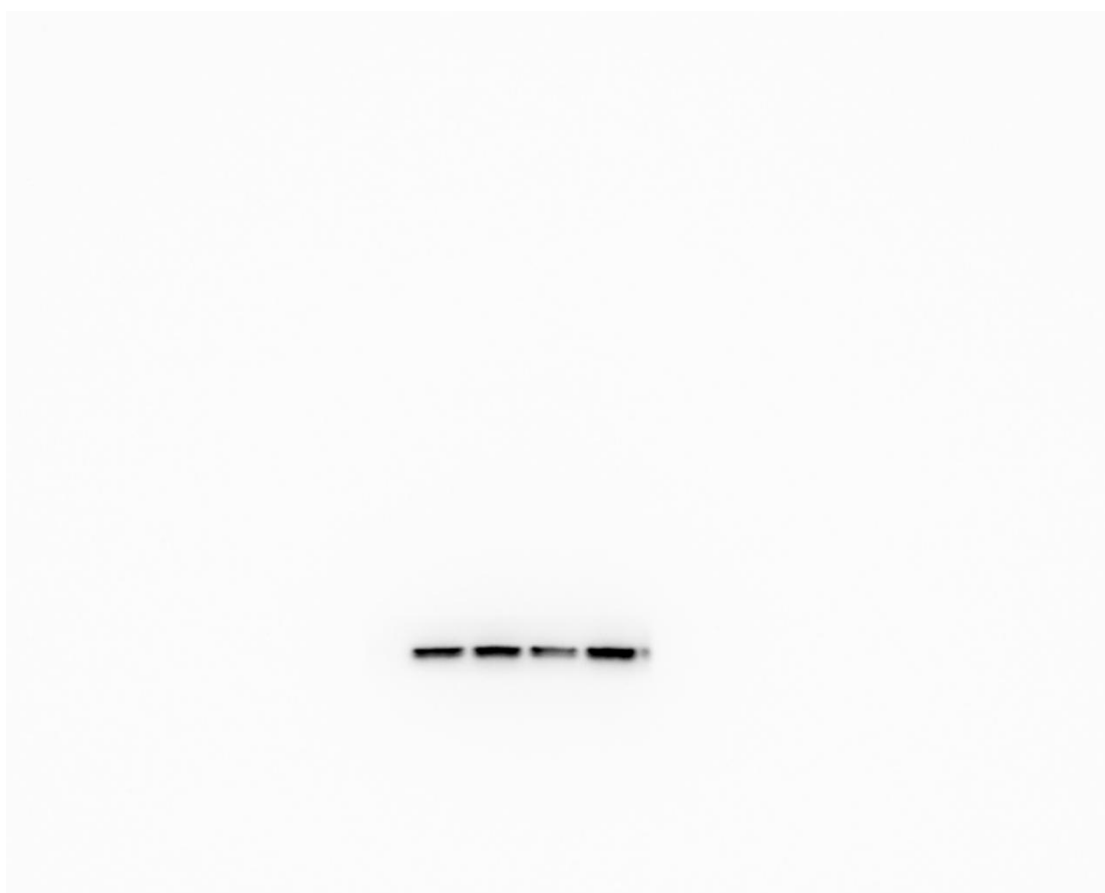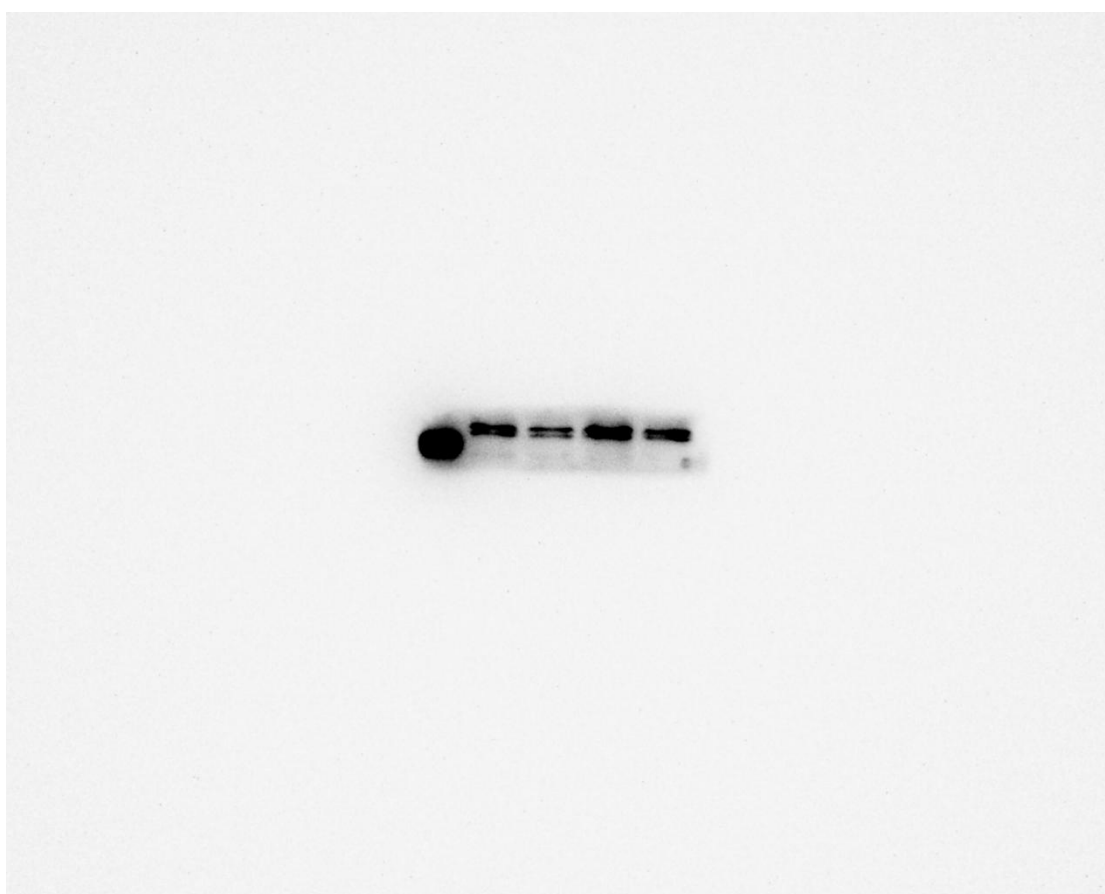

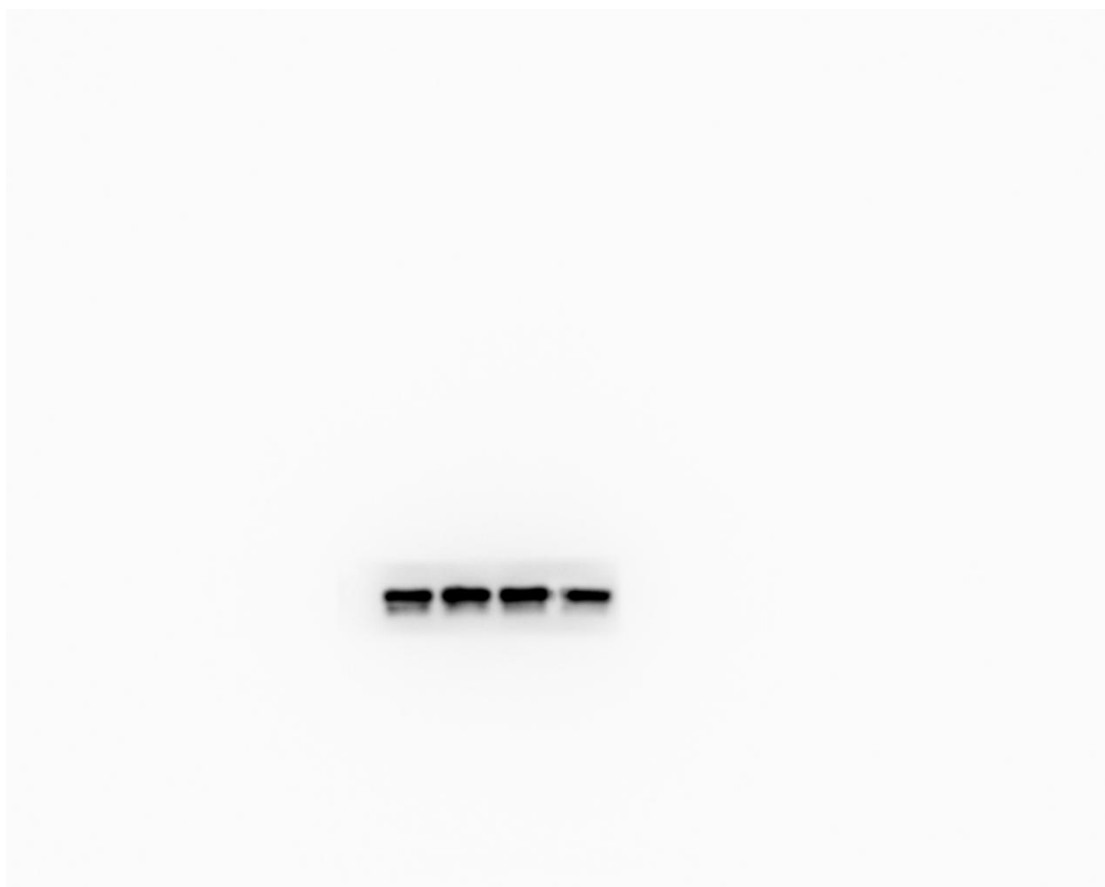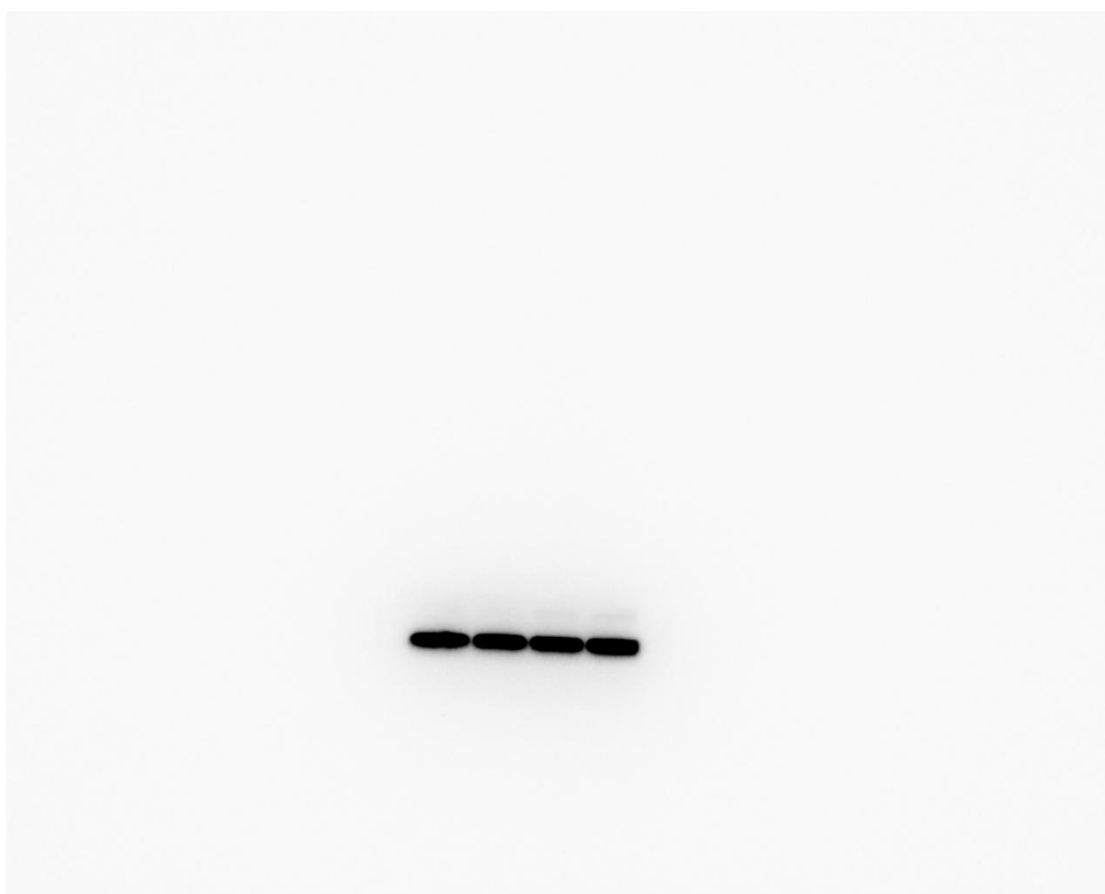

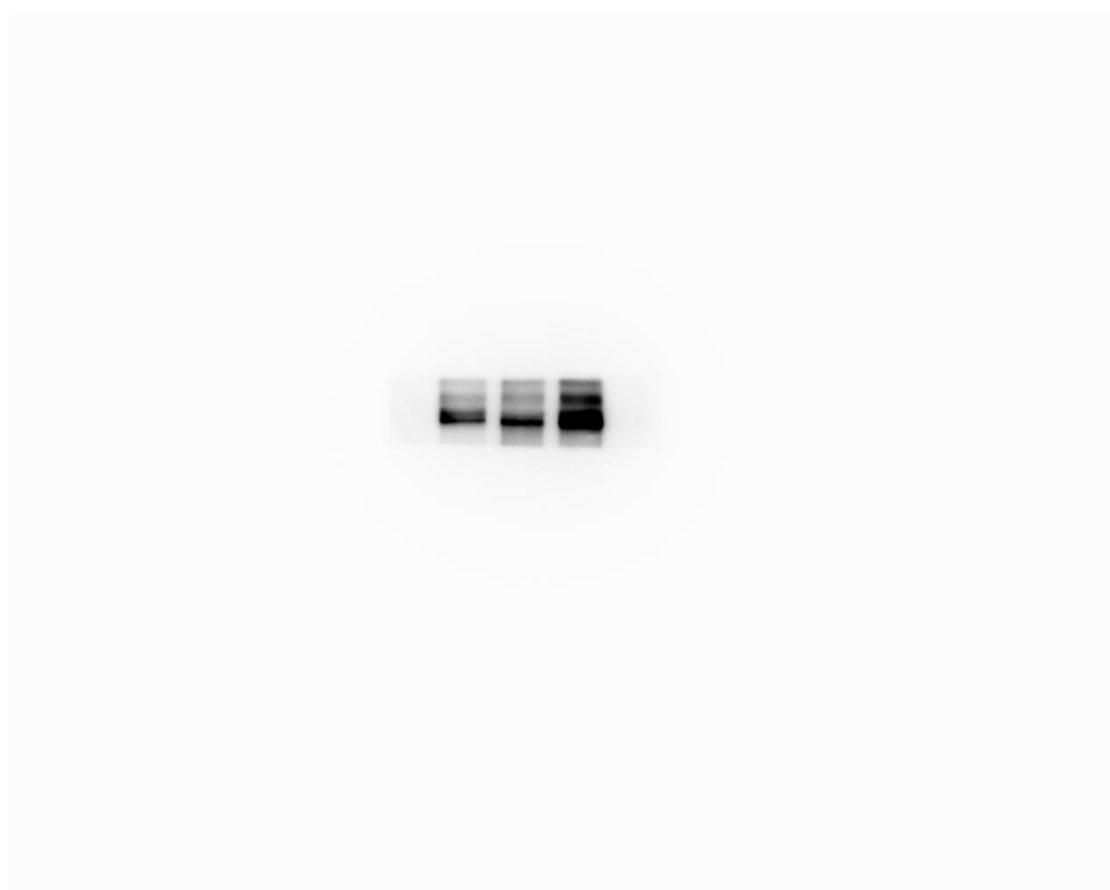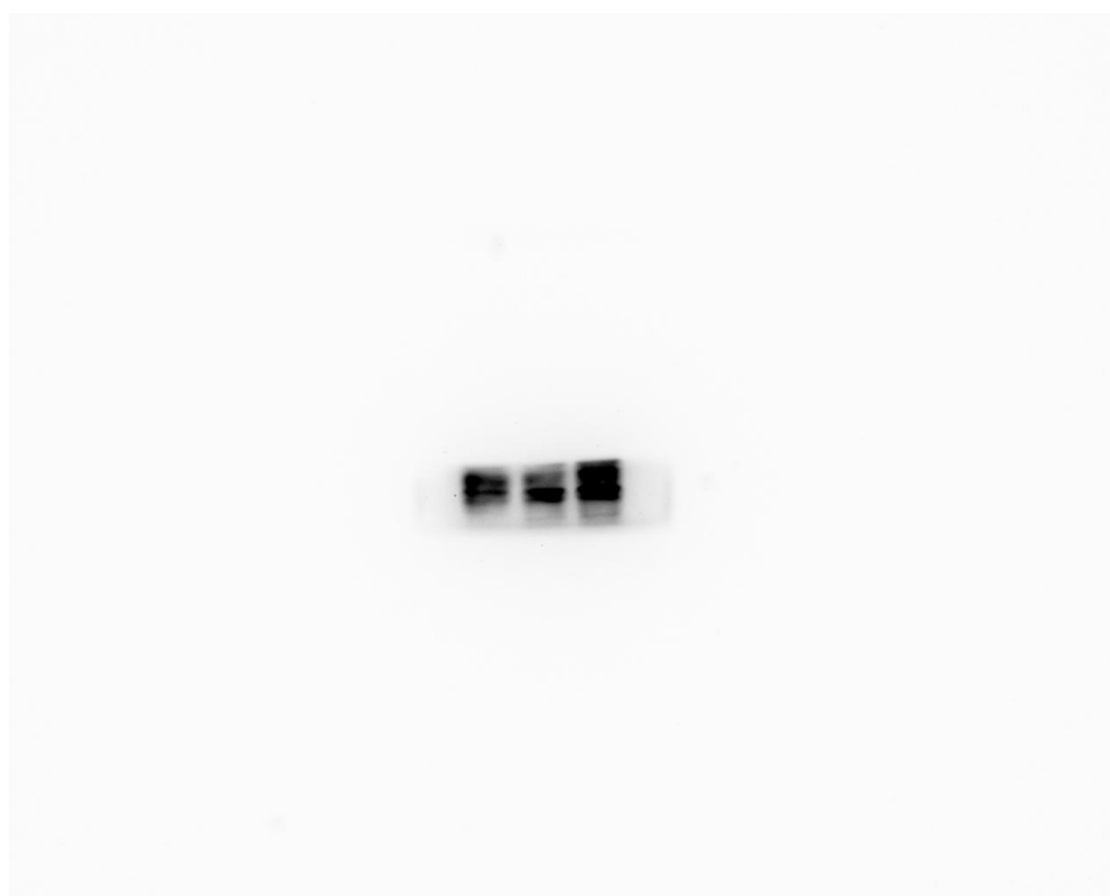

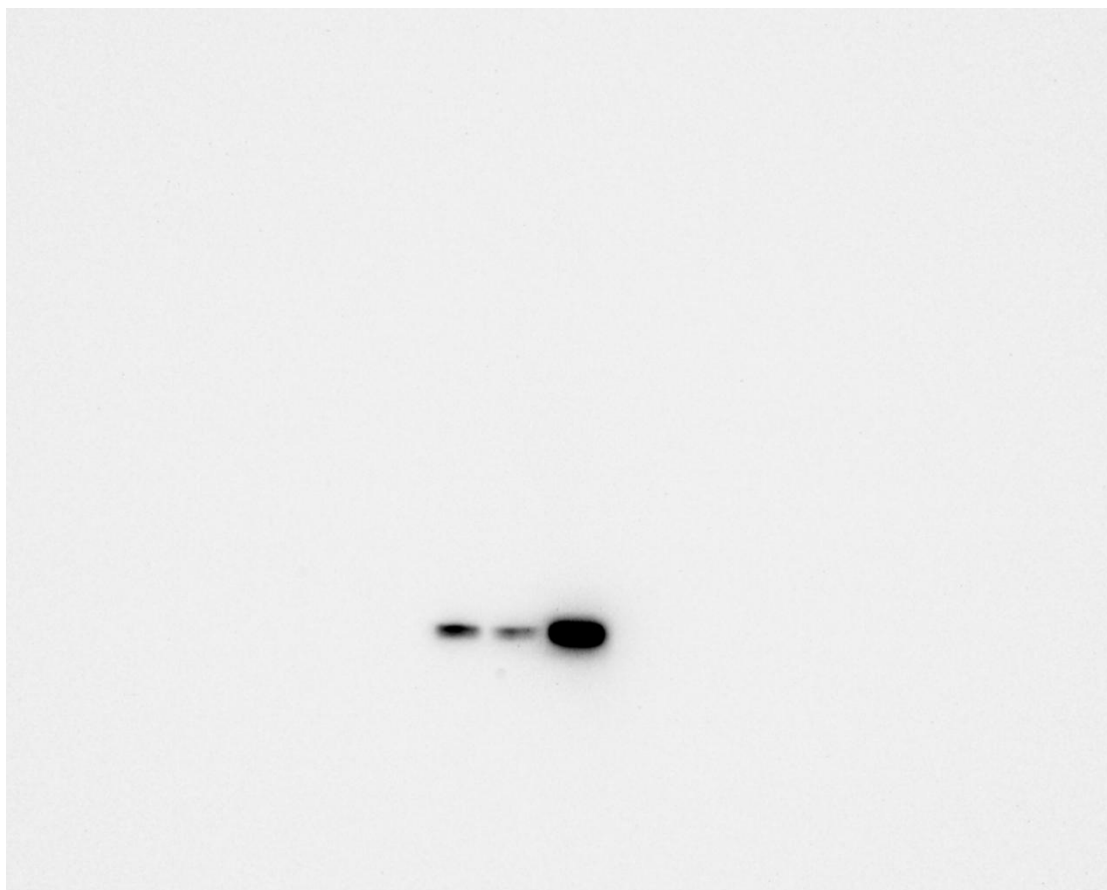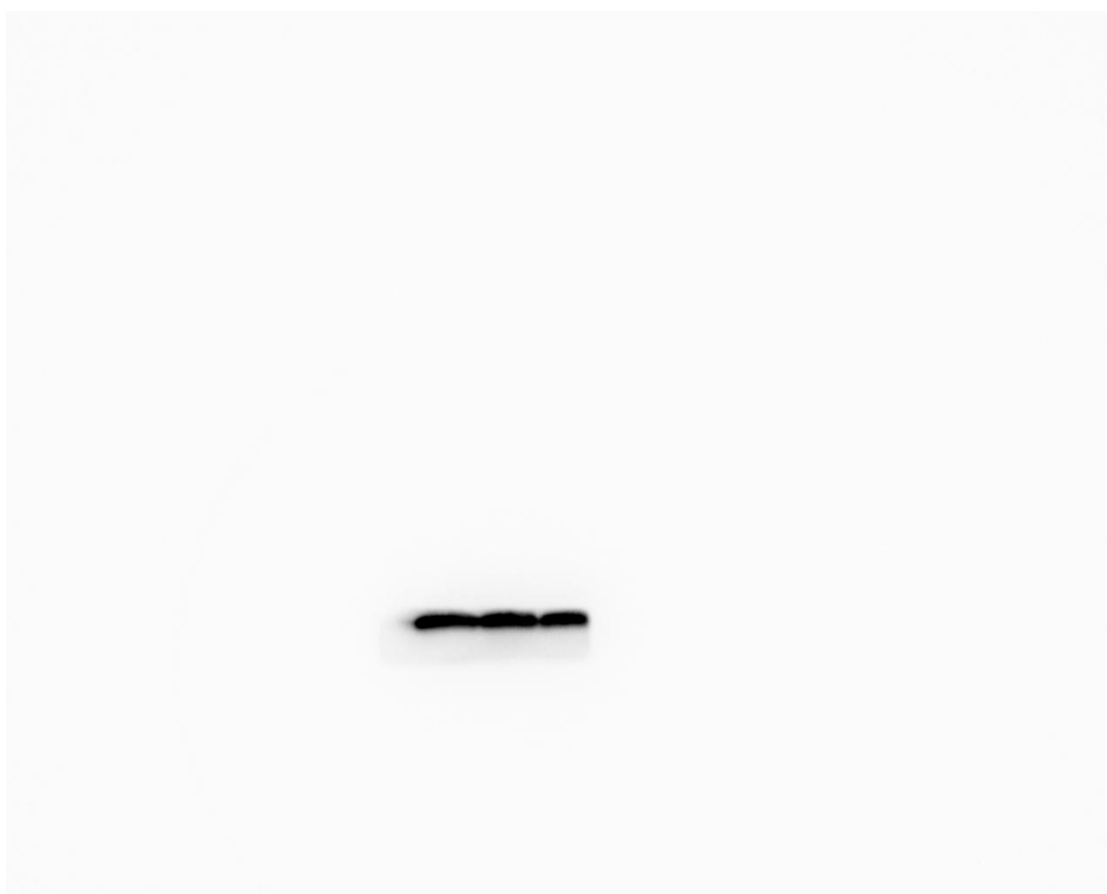

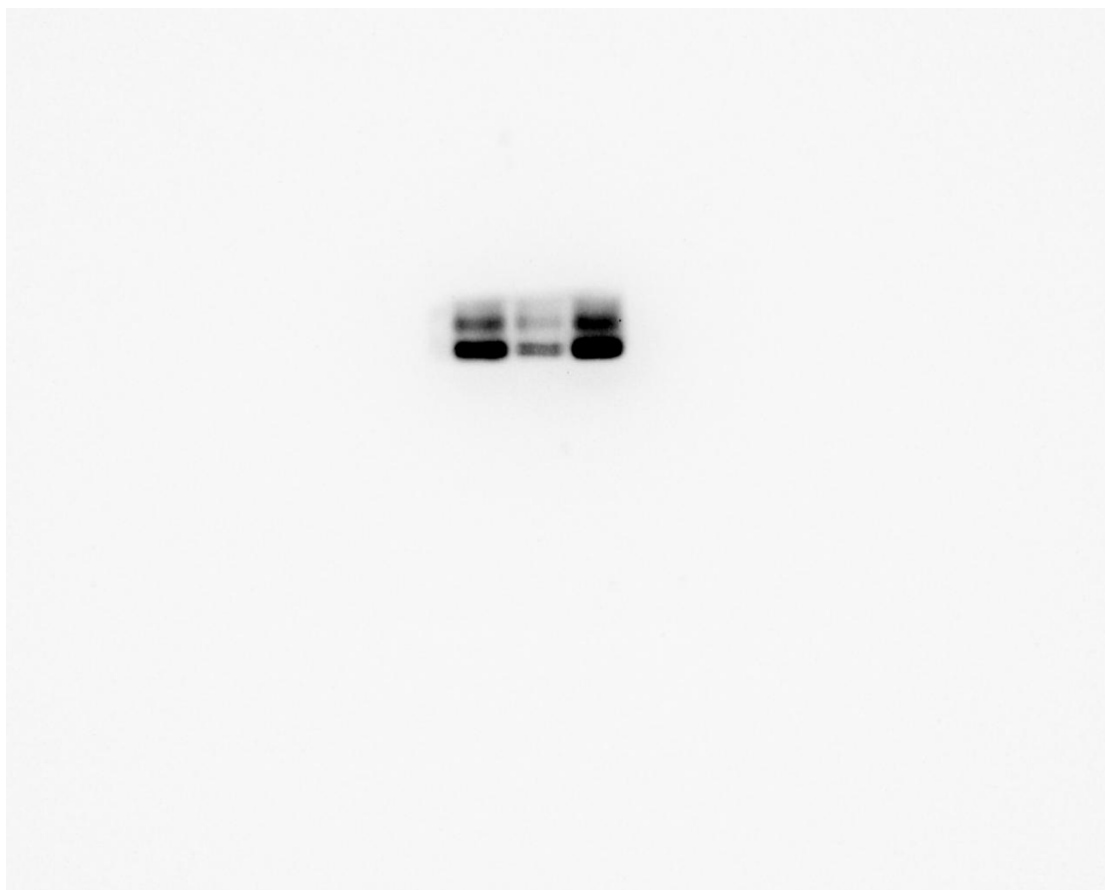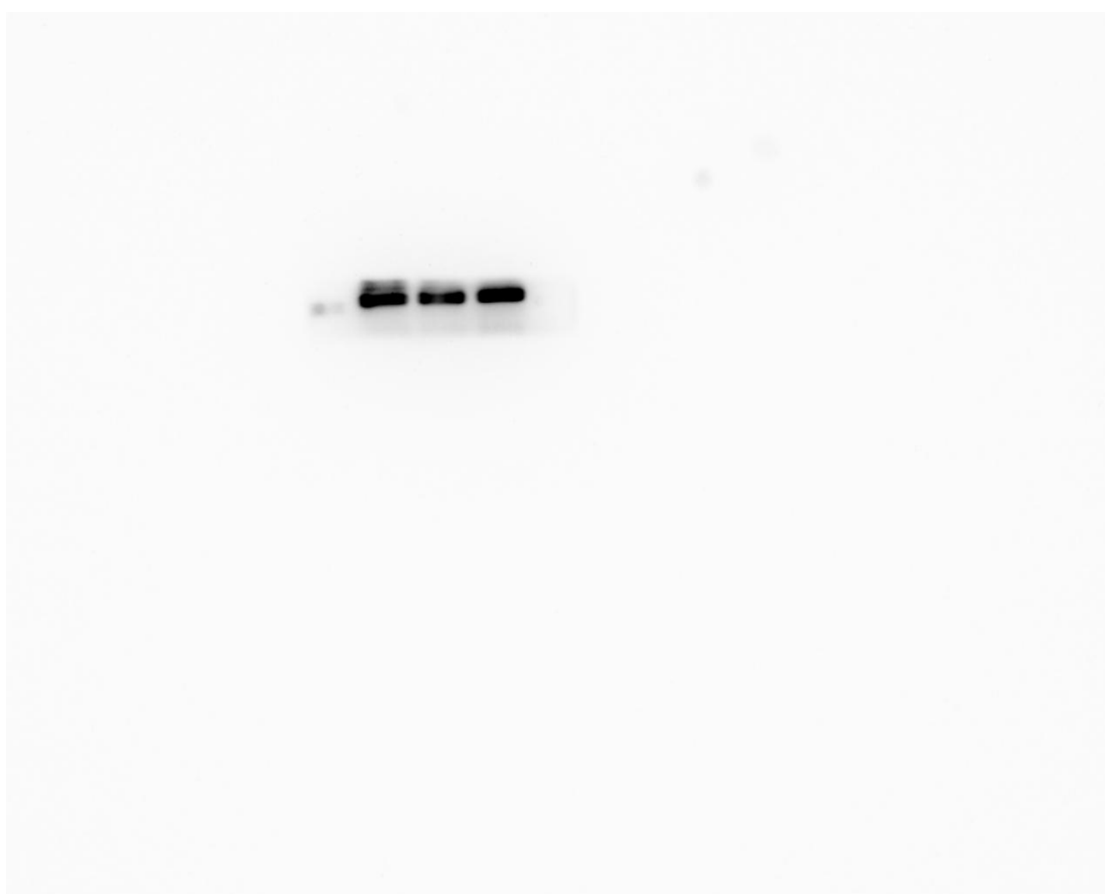

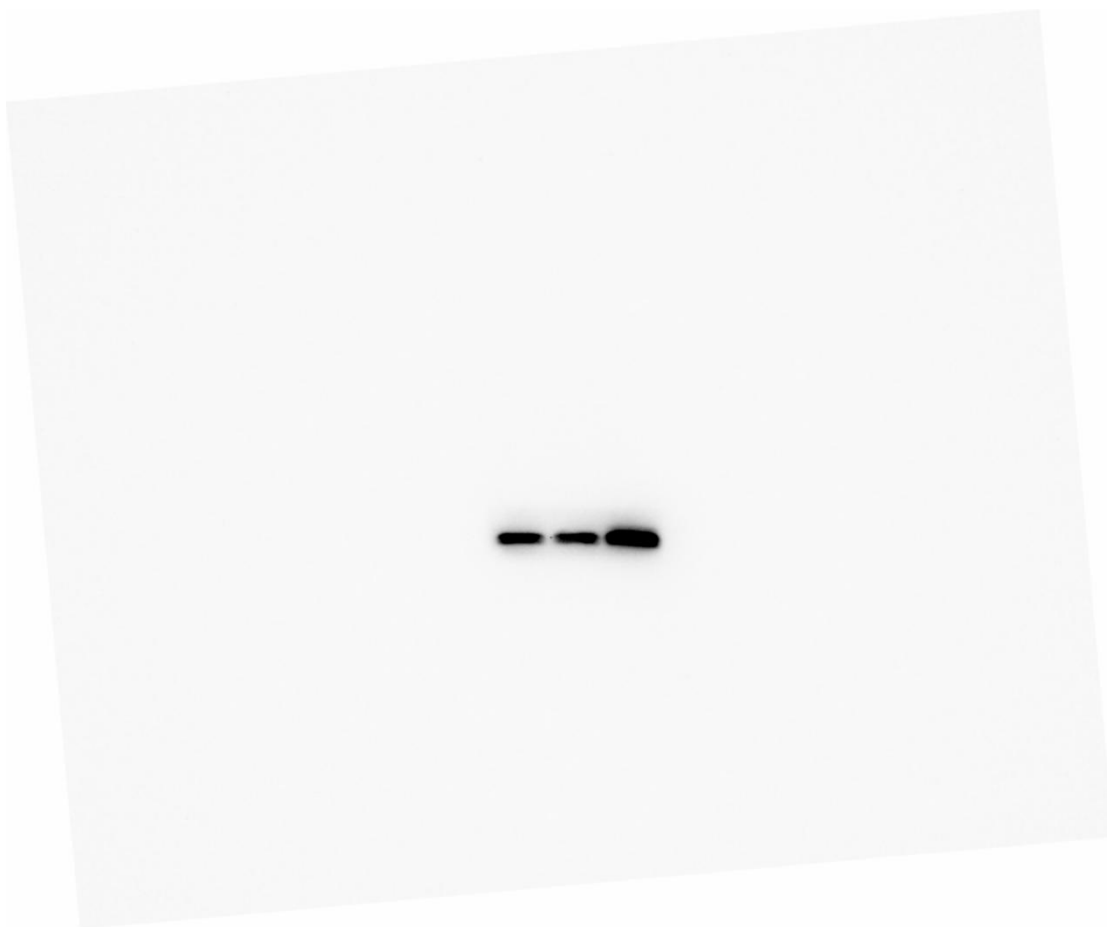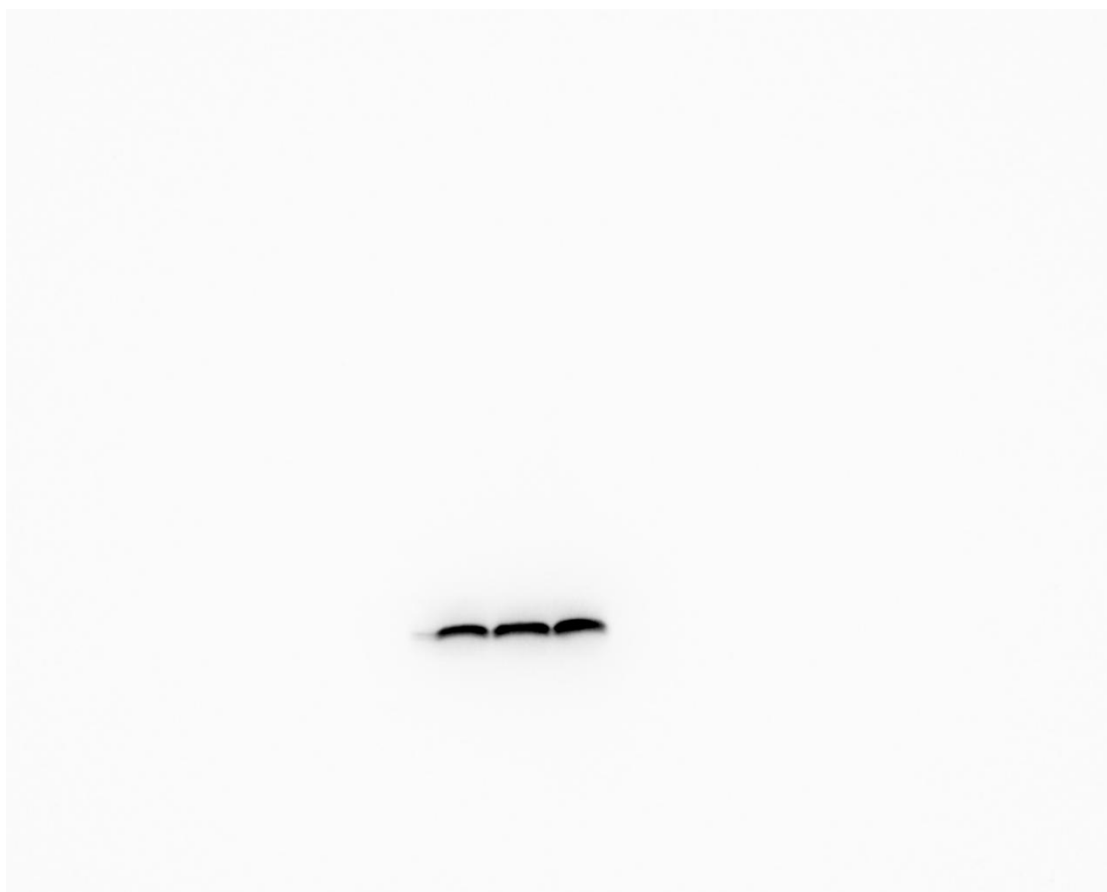

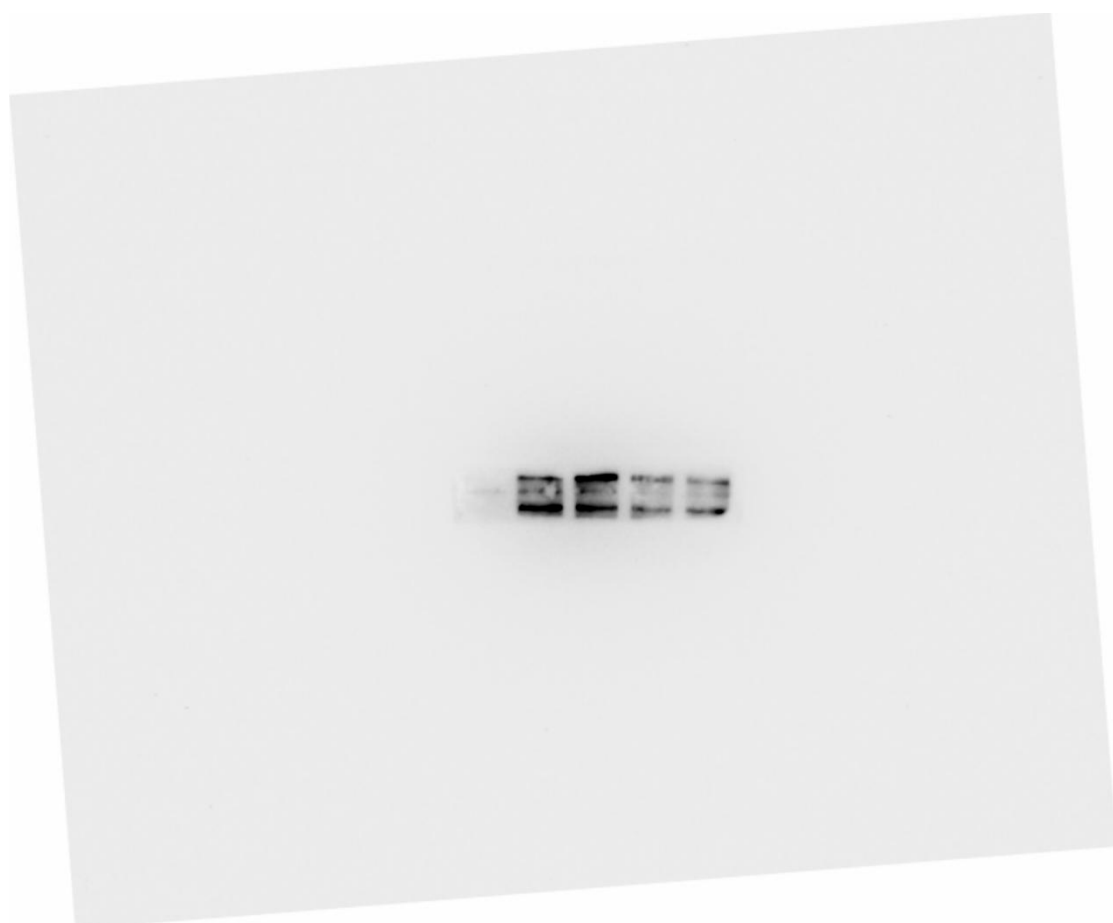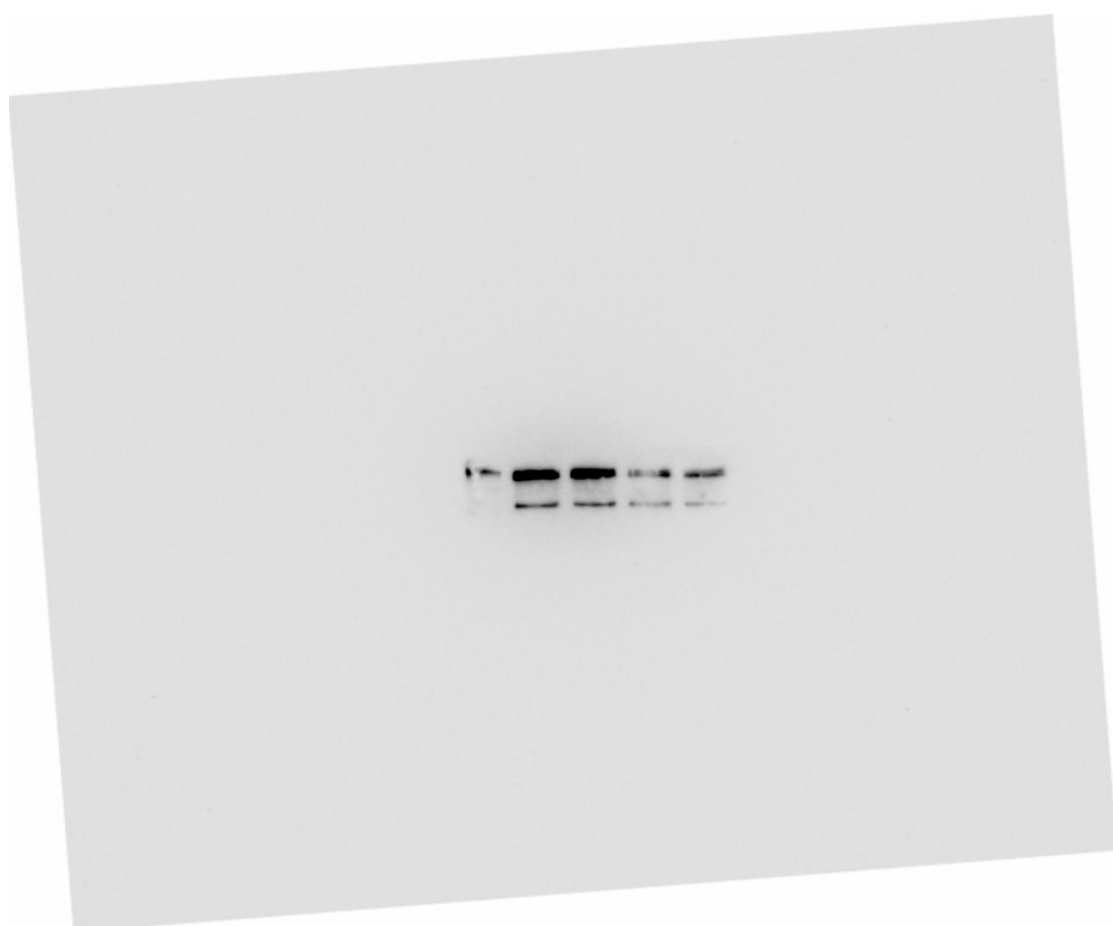

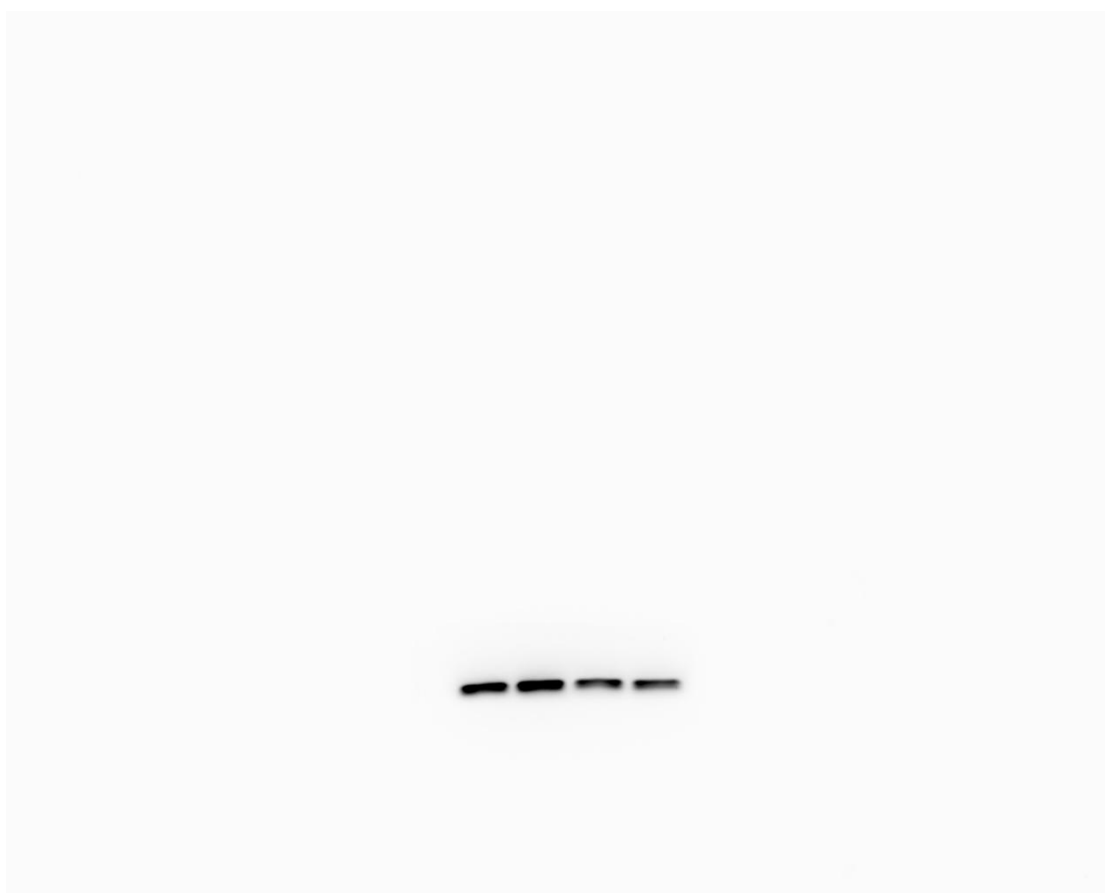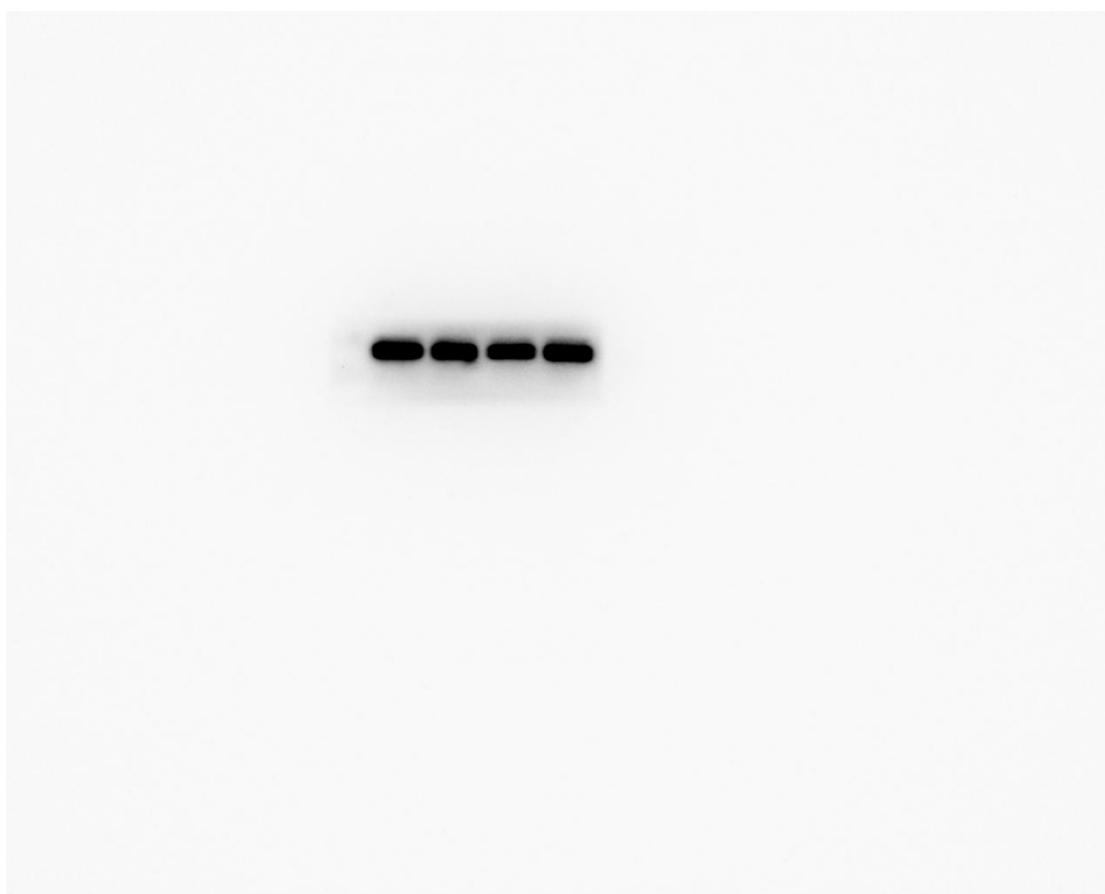

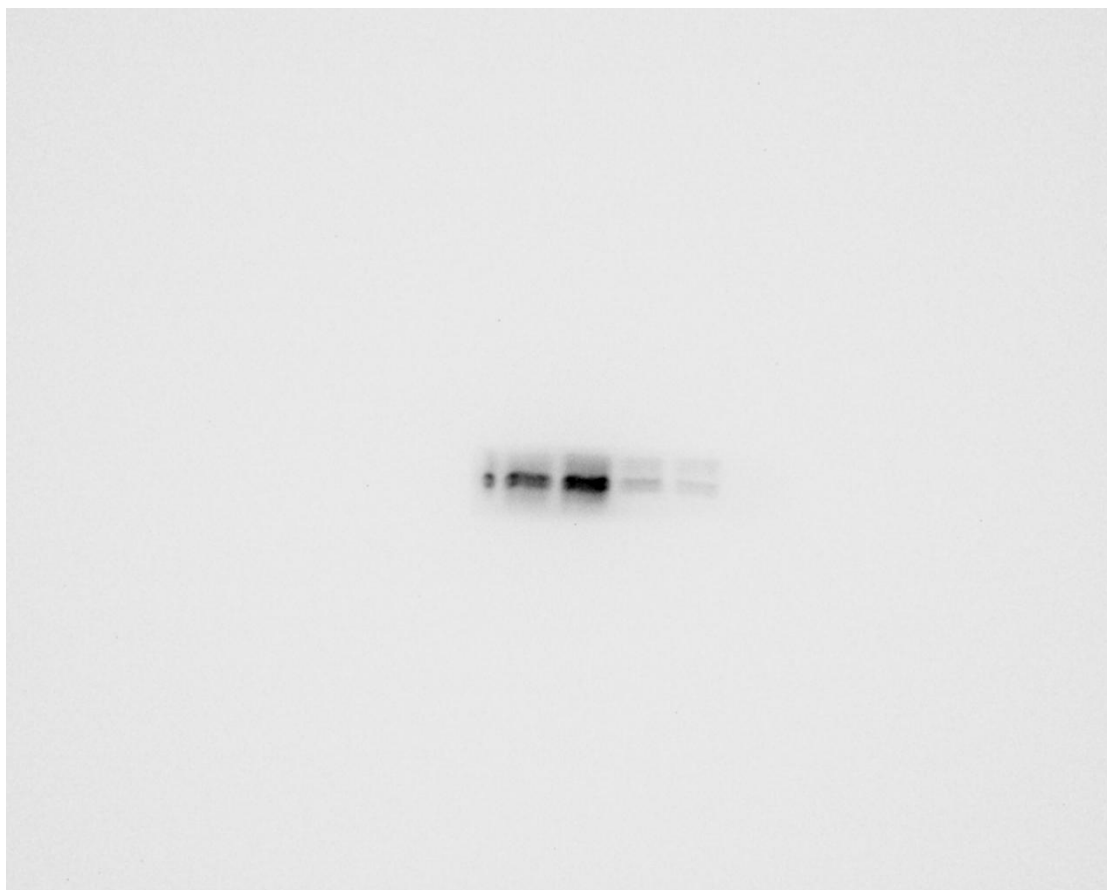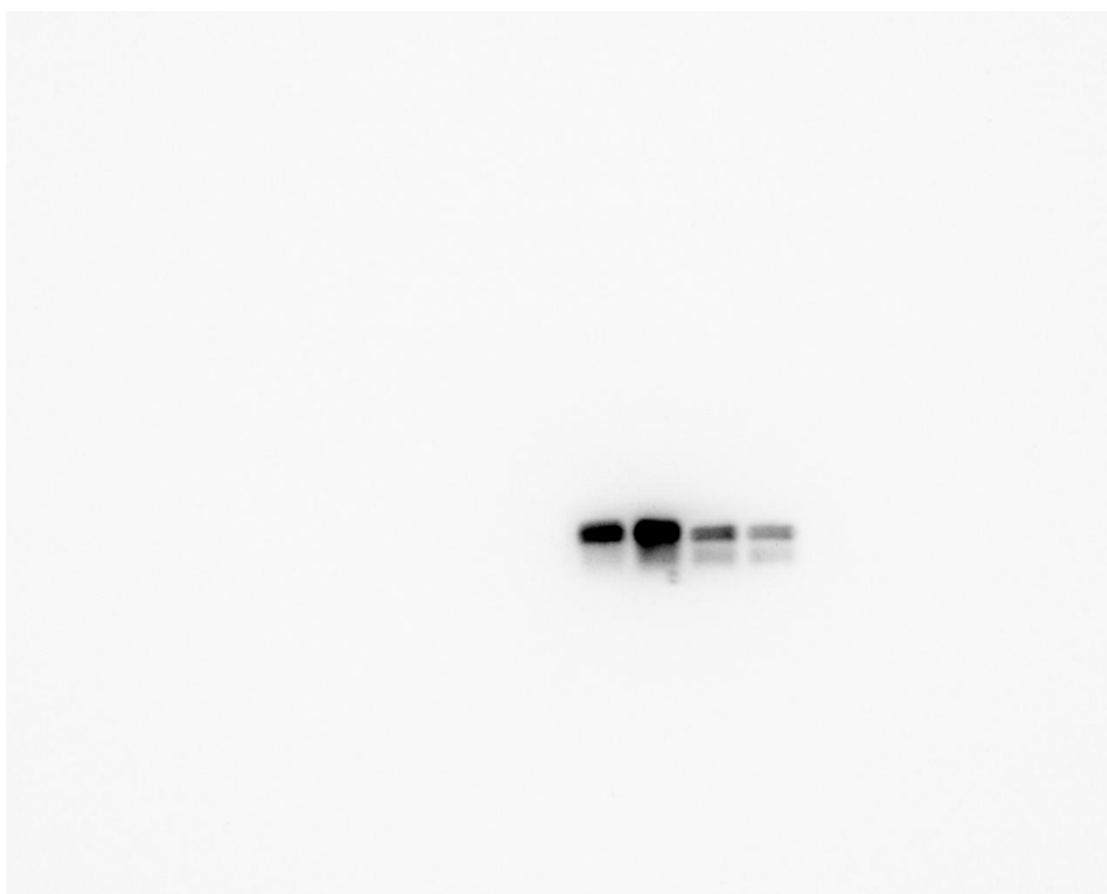

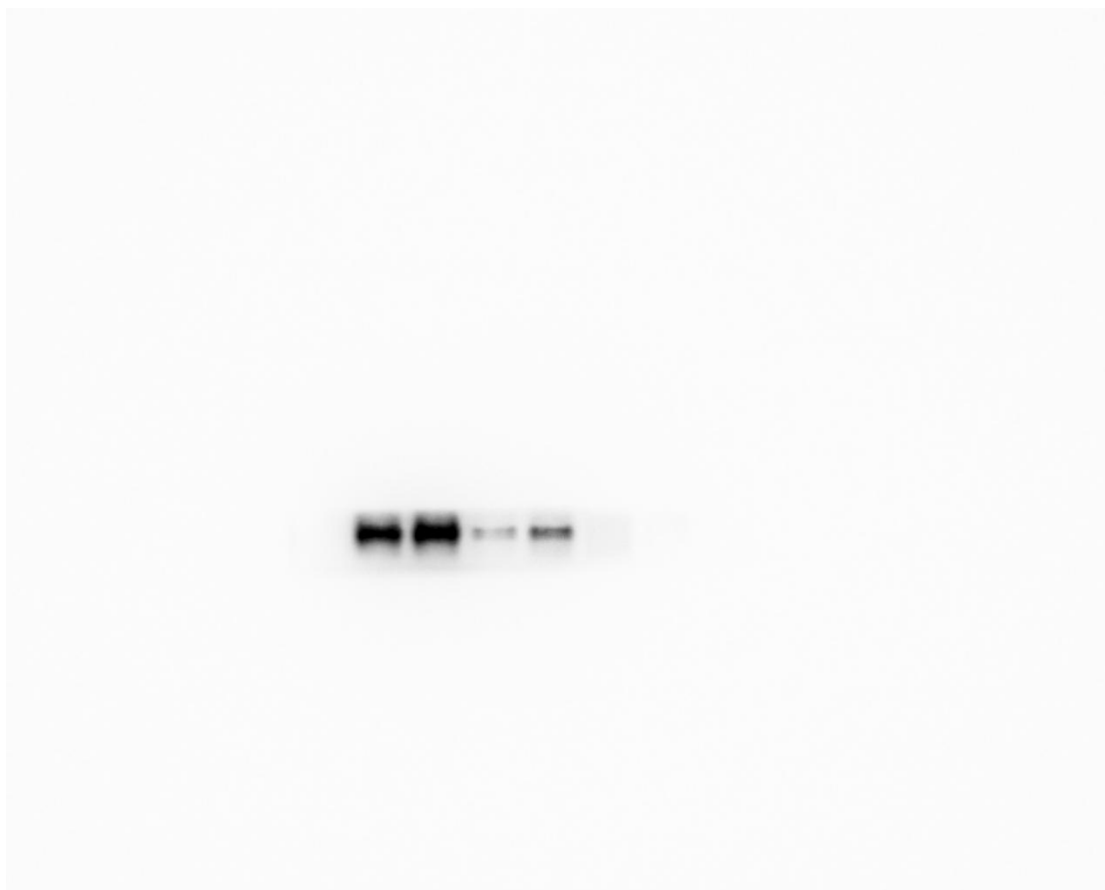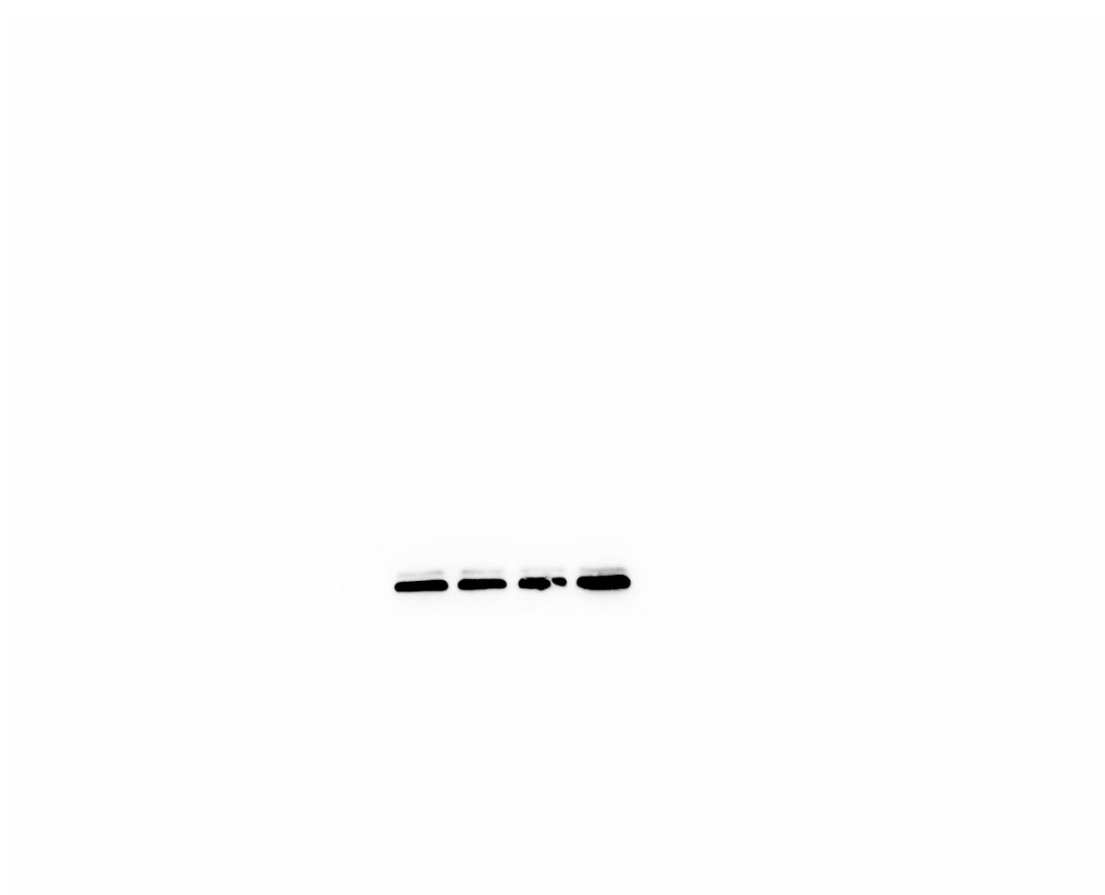

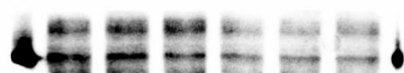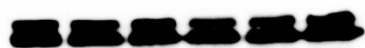

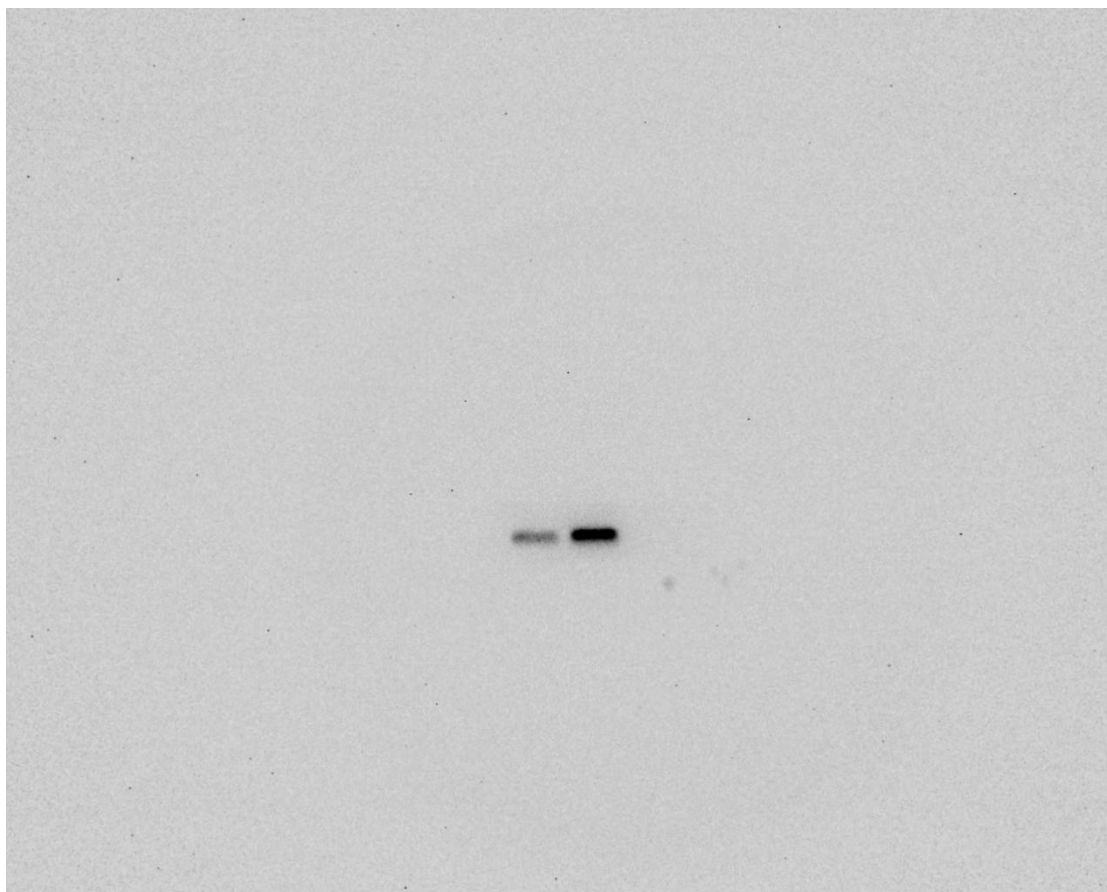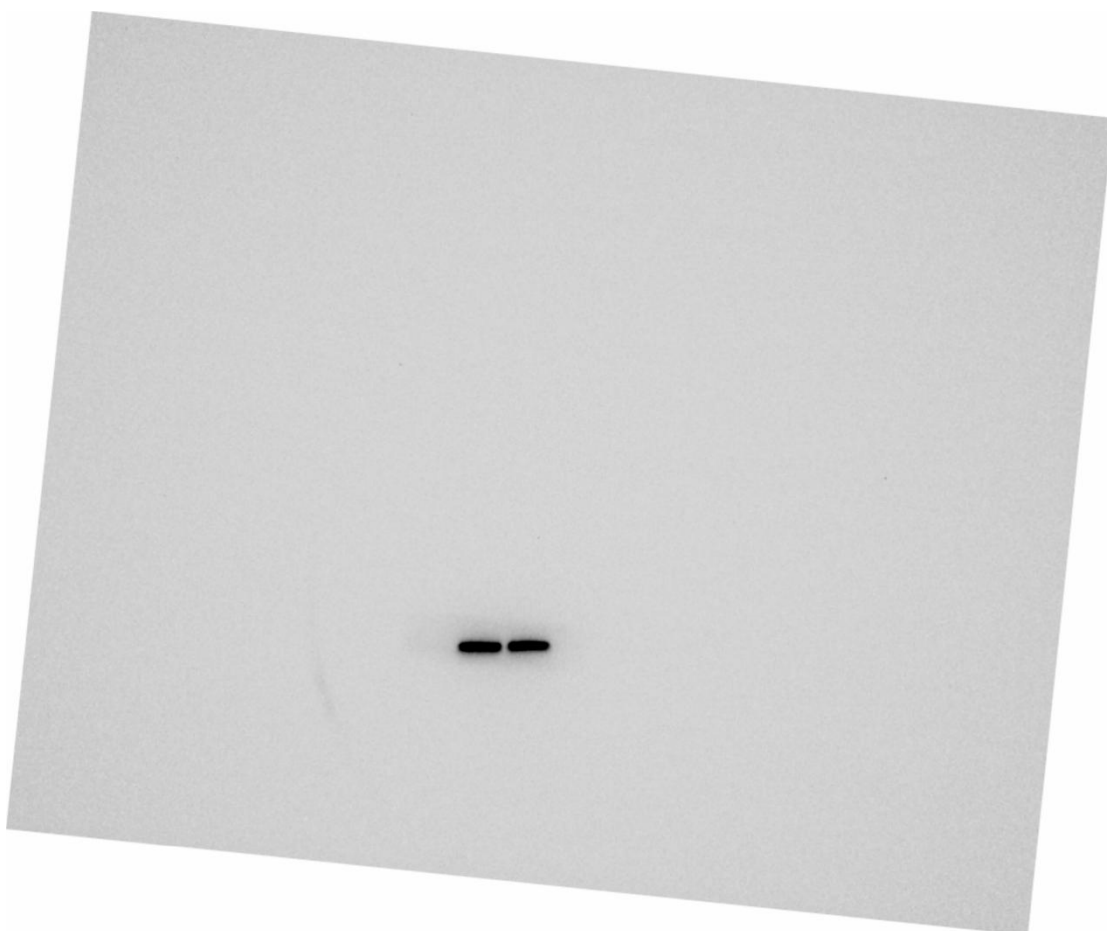

Supplement: Supplementary file 11 — Original uncropped western blot strips [file 41420_2023_1323_MOESM11_ESM.pdf]
